# An extended EDAS model for sustainable supplier selection in food supply chain management using interval-valued Pythagorean fuzzy soft set

**DOI:** 10.1038/s41598-026-45503-3

**Published:** 2026-04-27

**Authors:** Rana Muhammad Zulqarnain, Hamza Naveed, Hassan Naseer, Rifaqat Ali, Abdullatif Ghallab, Imran Siddique, Sohaib Abdal

**Affiliations:** 1 School of Business, Xian International University, Xian, 710077 Shaanxi China; 2 Department of Computer Engineering, Biruni University, Istanbul, 34010 Turkey; 3 Department of Mass Communication, Virtual University of Pakistan, Lahore, 54000 Pakistan; 4 Department of Mathematics, College of Science, King Khalid University, Abha, Saudi Arabia; 5 Faculty of Computing and Information Technology, University of Science and Technology, Sana’a, Yemen; 6 Department of Mathematics, Faculty of Science, Islamic University of Madinah, 42351 Madinah, Saudi Arabia; 7 Department of Mathematics, University of Sargodha, Sargodha, 40100 Pakistan; 8 Research Center of Applied Mathematics, Khazar University, Baku, Azerbaijan; 9 Department of Mathematics, Saveetha School of Engineering, SIMATS Thandalam, Chennai, 602105 Tamilnadu India

**Keywords:** Interval-valued Pythagorean fuzzy soft set, Einstein ordered AOs, Einstein hybrid AOs, EDAS method, Multi-attribute group decision-making, Sustainable food supplier selection, Engineering, Environmental sciences, Environmental social sciences, Mathematics and computing

## Abstract

Determining a sustainable food supplier is a decision-making problem with inherent uncertainty. The interval-valued Pythagorean fuzzy soft set (IVPFSS) is a type of fuzzy set that provides a wider range of solutions for presenting fuzzy and unreliable data. Therefore, every alternative is determined by evaluating multiple factors, including community involvement, staff health, financial stability, fostering imaginative thinking, business growth, minimizing carbon footprints, eliminating waste, and composting. This research mainly describes the interval-valued Pythagorean fuzzy soft Einstein-ordered weighted and Einstein hybrid weighted aggregation operators (AOs). The Evaluation based on the Distance from the Average Solution (EDAS) is a unique decision-making method that uses proposed aggregation operators to address multi-attribute group decision-making (MAGDM) problems. Implementing the proposed strategy substantially influences the selection of the most economical food supply chain management supplier. The practicability of our recommended approach is demonstrated through an empirical investigation focused on identifying a particularly productive vendor in the organic food sector. Comparative and sensitivity analysis reflect the predictability and efficacy of this approach and determine that the planned methodology is more realistic and feasible than conventional methods. The outcomes indicate that the proposed strategy offers a viable solution to address the challenges of sustainable food supplier selection (SFSS) with unclear facts.

## Introduction

Industries and economies worldwide struggle to meet evolving demands for client retention without centralised supply chain management (SCM) systems. SCM can be summarized as developing a connected network of organizations, vendors, modes of transport, logistics hubs, and industrial sites. The most crucial goal of supply chain executives is to balance all factors within the supply chain structure. This integration is a state in which it is secure, reliable, stable, sustainable, and supportive between multiple components over the product’s whole life cycle. As the primary inspiration, suppliers can significantly impact the network’s performance in various ways. Maintaining global competitiveness and maximizing benefits from supplier interactions is continually influenced by supplier connections, growth initiatives, and an experienced supplier network. By recognizing the importance of transparent manufacturing operations and the careful development of producer–consumer interactions, food supply chain management (FSCM) supports a nation’s socioeconomic development. End users of agricultural and food items are increasingly eager to understand more about their food journey from farm to fork. That is to say, customers are eager to learn about regulations on hygiene, packaging, and manufacturing practices, as well as the safety and quality of products and the acquisition process^1^. Wognum et al.^2^ suggested that food and agricultural businesses consider these criteria in their supply chain activities to meet customer needs and ensure customer satisfaction. In addition to social factors, a well-designed FSCM requires effective, beneficial supply chain operations.

The global food sector has been significantly impacted by high and volatile food costs, which are attributed to regional conflicts such as the Russian-Ukrainian war, unpredictable climate, and variations in the fiscal policies of prosperous nations. The United Nations Food and Agriculture Organization has released its latest grain import report. Initial estimates for the 2023/24 period suggest a potential 1.0% increase in worldwide grain cultivation, bringing the total to 2.803 billion tons^3^. Still, the uncertainty surrounding conflicts and the factors contributing to violent problems has resulted in a worldwide malnutrition disaster. Moreover, food scarcity exacerbates environmental challenges and causes natural disasters. Tragically, approximately 29.6% of the global population, or nearly 2.4 billion people, lack consistent access to food. This became apparent by the frequency of profound or moderate food insecurity. A severe food shortage affected almost 900 million people. A novel TOPSIS method using the Pythagorean fuzzy set was presented by Hajiaghaei-Keshteli et al.^4^ and is intended for use in the packing processes of food enterprises. Yazdani et al.^5^ developed a two-phase, multi-tier sustainable supplier model and integrated decision evaluation with sustainability standards to rate sub-suppliers and suppliers. Wang et al.^6^ proposed a structure based on the Pythagorean fuzzy choice strategy to address the problem of selecting sustainable food suppliers arbitrarily. A multi-stage Best Worst Method for PFS, based on sustainability and diffuse environmental norms, was proposed by Kazançoglu et al.^7^ to efficiently execute multi-tier supply chains in the food industry.

Suppliers are important in SCM networks, and their ability to operate effectively has an immediate or subsequent impact on the overall functioning of the process. This extends to multiple sectors, including manufacturing goods, services, and food^8^. Transport sector enterprises, logistics firms, and ingredient suppliers are essential components of a supply chain network for every mainstream business. This approach reduces the risk of supply chain loopholes and encourages the development of a more capable manufacturing structure^9^. Previous studies have confirmed that supplier performance evaluation plays an essential role in improving the productivity of SCM systems^10^. Yilmaz & Karadayi-Usta^11^ examined how participant organizations in the sports tourism supply chain can support resilience using a spherical fuzzy MACTOR method.

Experts design several studies to strengthen the conceptual foundations of traditional fuzzy sets (FS). Among those are: the interval-valued fuzzy set (IVFS)^12^, the intuitionistic fuzzy set (IFS)^13^, the interval-valued IFS (IVIFS)^14^, the Pythagorean fuzzy set (PFS)^15^, and the interval-valued PFS (IVPFS)^16^. Their parametric representational alternatives need to be simplified so that the previous structures can be grasped. A unified logical structure that can negotiate to handle these challenges is the soft set (SS)^17^. Multiple studies have hybridized FS with SS, notably intuitionistic fuzzy soft sets (IFSS)^18^, interval-valued IFSS (IVIFSS)^19^, Pythagorean fuzzy soft sets (PFSS)^20^, and interval-valued PFSS (IVPFSS)^21^. The concept of IVPFSS is a valuable framework that employs membership $\left(\lambda \right)$ and non-membership $\left(\eta \right)$ grades within intervals that the condition can identify ${\left({\lambda }^{\mathpzc{u}}\right)}^{2}+{\left({\eta }^{\mathpzc{u}}\right)}^{2}\le 1$.

However, contemporary research rarely prioritizes selecting food providers with an ecological perspective, especially in an intricate and volatile context. Therefore, it is necessary to develop a comprehensive DM framework based on enlarged fuzzy sets to manage supply chain operations in the presence of intricate inconsistencies. The existing literature shows that this method of expressing problems via FS is widely used to model exacerbated concerns^4,7^. Implementing membership degree (MD) based FS in real life is limited by its inability to account for the non-membership degree (NMD), which is often convoluted with a professional’s questionable intelligence. As the sum of the squares of both degrees in the PFS cannot exceed 1, it includes more MD and NMD possibilities than the IFS. Several companies used PFS to identify preferred suppliers, including those in new energy^22^, manufacturing^23^, machinery manufacturing^24^, and food^4^.

Although the more beneficial FSCM hasn’t leveraged the IVPFSS to address complex sustainability-related concerns, it has integrated the IVPFSS into organic food supplier selection, using this information. Also, an essential phase in solving the sustainable food supplier selection issue using IVPFSS facts is an overall assessment of individual personal preferences. It indicates that the sustainable food supplier selection process needs to account for these discrepancies. Therefore, to contend with the abnormalities, it is essential to implement Einstein-ordered and hybrid AOs. In this research, we deliver an EDAS method to tackle MAGDM challenges in the IVPFSS context by implementing Einstein AOs.

The complete structure of this research paper is reflected in this summary. In Section "Introduction", the significance of assessing ambiguity and insufficient information in DM is highlighted. A comprehensive review of the literature on supplier selection problems in various fuzzy extensions is presented in Section "Literature review". Section "Preliminaries" discusses in detail the basic concepts and principles that support ongoing research. In section "Interval-valued Pythagorean fuzzy soft Einstein weighted average aggregation operators", the Einstein operational laws for IVPFSS are defined based on the IVPFSEOWA and IVPFSEHWA operators, and their preferred distinctive features are identified. Then, in Section "Interval-valued Pythagorean fuzzy soft Einstein weighted geometric aggregation operators", the IVPFSEOWG and IVPFSEHWG operators, along with their main properties, are presented. A sophisticated EDAS model for addressing MAGDM difficulties, based on our built Einstein AOs, is introduced in Section "The evaluation based on the distance from the average solution (EDAS) model using Einstein-ordered and hybrid AOs". In Section "Application of the proposed EDAS method in sustainable food suppliers", a detailed case study is presented on selecting the best supplier for FSCM. Section "Comparative analysis and discussion" compares our proposed model with existing studies in a meticulous manner to substantiate the model.

## Literature review

This section exhaustively reviews the relevant studies on SSS in FSCM. Then, we briefly review the Einstein AOs and the EDAS method. This research explores the fundamental reasons and accomplishments related to its subject matter.

### Fuzzy MAGDM techniques in sustainable supplier selection

The MAGDM strategy is an efficient method for managing the challenges of SSS in complex and uncertain circumstances, as previously stated. Multiple models that employ fuzzy MAGDM strategies have been reported in the academic literature to address supplier selection challenges across various industries. Samantra et al.^25^ extended the VIKOR method in fuzzy structure to resolve MAGDM problems in supplier selection. Mishra et al.^26^ explored the problems of selecting suppliers in a group DM scenario with competing and non-commensurable factors using the VIKOR technique. Jiang et al.^27^ extended the MABAC approach for picture-fuzzy sets to determine the best vendor among four suppliers. Yu et al.^28^ introduced the TOPSIS technique for IVPFS and applied it to determine sustainable suppliers. Xing et al.^29^ proposed the Choquet Integral-based interval type-2 trapezoidal fuzzy MAGDM approach for SSS. Zhang et al.^30^ presented a spherical fuzzy GRA in MAGDM that integrates the psychological factors apparent by cumulative prospect theory with a spherical fuzzy setting.

Wang^31^ proposed an additive ratio evaluation technique for a linguistic T-spherical fuzzy structure to identify a sustainable supplier in the power battery industry. Wu et al.^32^ proposed a MAGDM strategy to identify reliable retailers by combining the DEMATEL and VIKOR methods. Liu et al.^33^ proposed the Aczel-Alsina operational Heronian mean operators for q-ROFS and developed a MAGDM technique to identify a sustainable healthcare supplier. Wang et al.^34^ presented a MAGDM model for supplier selection in SCM within an IVq-ROFS framework. Zulqarnain et al.^35^ developed the interactional AOs within the IVPFSS and extended a DM technique to choose a supplier capable of maintaining sustainability. Moreover, they proposed the TOPSIS strategy within the IVPFSS environment and implemented their designed method to select an ETL^36^. Kirişci et al.^37^ developed an integrated WASPAS method for interval-valued Fermatean fuzzy sets to compute objective and subjective weights for more realistic outcomes and applied it to green supply chain management. Rashid et al.^38^ resolved the complications of low-carbon supply chains using the AOs in an N-cubic fuzzy structure. Choudhary et al.^39^ introduced a set of key performance indicators for sustainable supply chain management using the MADM technique for IFS. Alkan and Kahraman^40^ extended a novel MADM technique to a continuous Pythagorean fuzzy environment to demonstrate its effectiveness in green supplier selection and waste disposal site selection.

Previous studies on sustainable supplier selection in FSCM faced multiple limitations that hindered their applicability in multifaceted, real-world contexts. Traditional decision-making techniques rely on fuzzy or intuitionistic fuzzy structures to accurately handle uncertain data in both membership and non-membership degrees. Moreover, these structures cannot be used for decision-making because they are focused on crisp or limited fuzzy data, which makes it difficult for them to accommodate the dynamic nature of modern supply chains. On the other hand, advanced fuzzy models, such as IVPFSS, can manage both MD and NMD simultaneously and provide a robust, precise mechanism for sustainable supplier selection. Multiple studies are unable to address MAGDM complications because they neglect interactions among experts’ opinions for the considered attributes.

Meanwhile, our proposed MAGDM technique based on Einstein-ordered and hybrid AOs aggregates the experts’ evaluations more energetically and analytically. Also, traditional models are disposed to implement fixed assessment replicas that cannot account for the evolving nature of supplier performance over time. But our developed MAGDM model addresses these shortcomings by providing a reliable decision-making process that accounts for changes in supplier performance and external factors.

### Einstein aggregation operators for distinct MAGDM problems

Different kinds of AOs are designed and used in different ways to accurately measure various situations, which has generated significant interest and importance in this area of research. Wang and Liu^41,42^ suggested the use of Einstein AOs in IFS to tackle the challenging problems of MAGDM. Liu et al.^43^ promoted the use of Einstein averaging AOs as a sufficient means to solve the issues of MAGDM using IVIFS. Likewise, Wang and Liu^44^ extended the application of Einstein geometric AOs to include IVIFS and developed a DM method to address the issues posed by MAGDM. Garg^45,46^ introduced several AOs using the Einstein operational laws for PFS. Rehman et al.^47,48^ proposed Einstein AOs based on Einstein operational laws for IVPFS and established a DM technique to accommodate the MAGDM challenges.

After a detailed investigation of the relevant studies, a shortage of studies was found regarding the implementation of Einstein’s operational rules to develop advanced operators for IVPFSS. Due to the frequent existence of information in interval form in multiple real-world businesses like finance, hospitals, banking, and engineering, it is necessary to possess the aptitude to use IVPFSS in analysis tasks.

### Evaluation based on the distance from average solution (EDAS) method

Through a detailed assessment of multiple alternatives, the MAGDM technique enables them to identify the most effective alternative. A reliable approach for analytically describing MAGDM challenges that involves interacting aspects is the EDAS model, which integrates positive distance from average (PDA) and negative distance from average (NDA). This technique was originated by Ghorabaee et al.^49^. Ilieva^50^ introduced EDAS to address group DM complications within the IVFS structure. Mishra et al.^51^ used the EDAS approach for IVFS to manage hospital waste. Li and Wang^52^ explored the EDAS model within the IVIFS framework to optimize the performance of wireless sensor networks. Liu et al.^53^ prolonged the EDAS model in the PFS context. Akram et al.^54^ presented the CRITIC-EDAS approach to address industrial waste management issues in linguistic PFS. Yanmaz et al.^55^ developed the EDAS method for IVPFS to address the issues within MAGDM. The strategy with picture fuzzy sets was developed by Zhang et al.^56^. Mishra et al.^57^ applied the EDAS approach to determining the stability of the ranks of third-party reverse logistics suppliers based on attribute weight estimations. Despite the EDAS methodology’s prominence, research investigating its implementation in the framework of the IVPFSS structure has not yet been conducted.

This research presents an EDAS technique in the IVPFSS to determine the sustainable supplier in FSCM. This technique can handle the problems outlined below: How can the most beneficial vendor be selected despite unclear scenarios? How can we collect facts about suppliers’ capabilities? How can the EDAS method be implemented to generate a sustainable SSS procedure that can be managed over time?

### Motivation

The growing complexity of decision-making in sustainable supply chain management (SSCM) requires the development of more advanced and flexible tools that can effectively handle uncertainty and vagueness in real-world data. Einstein ordered hybrid AOs to effectively aggregate group decision-making. A novel EDAS method is developed for IVPFSS using Einstein-ordered and hybrid AOs to enhance the flexibility and accuracy of the decision-making mechanism. The existing Einstein AOs^41,42,45,46^ address MD and NMD, but none of them handle data from interval and parametric evaluations.

Meanwhile, the Einstein AOs developed in^44,47,48^ address only MD and NMD intervals, which are known for their flexibility and adaptability. Furthermore, these operators are not well-suited to the parametric evaluation of alternatives, creating a significant gap. This underscores the need to advance Einstein-ordered and hybrid AOs within the IVPFSS framework to effectively integrate complex, composite data. To date, the concept of Einstein-ordered, hybrid AOs for IVPFSVs has not been introduced, highlighting an important area for further development in MAGDM. The literature demonstrated that the sustainable supplier selection (SSS) approach is most effectively described as a problem integrating MAGDM. Since it involves parameters and alternatives, sustainable food supplier selection (SFSS) challenges are often examined as a MAGDM problem in such circumstances. Therefore, many methods are available for managing the SFSS problem, including TOPSIS, COCoSo, PROMETHEE II, and MACSONT. Compared to those methods, the EDAS approach evaluates alternatives using a novel and widely used methodology. It might be easier to use and take less time to perform the objective, and the findings could be more precise and reliable. Thus, it is refined to address supplier selection challenges in multiple areas^5,58,59^. Compared to previous strategies, the EDAS method uses simpler computations. In this instance, the EDAS model can address these obstacles using hybrid AOs and Einstein-ordered AOs.

Multiple fuzzy sets are used in the FSCM literature to communicate experts’ purpose and intellectual information^4,7^. Even the current selection of suppliers, which faces challenges with FSCM, has failed to consider the IVPFSS. Thus, the SFSS challenge was handled by establishing a decision structure using IVPFSS. An exhaustive review of FSCM techniques demonstrated that the IVPFSS framework needed an EDAS method to choose the best sustainable supplier. This research aims to present a novel EDAS method for solving MAGDM issues in IVPFSS environments. This strategy objectively evaluates sustainable food supply organizations by using allocated attribute weights.

### Contributions of the study

This research presents a selection methodology based on synthetically generated decision algorithms to improve the integrity of SFSS consequences, in light of the reasons mentioned previously. The main contributions of this research are summarized as follows:

1. Einstein operations are implemented into the IVPFSS model to build Einstein-ordered and hybrid AOs for IVPFSS, such as IVPFSEOWA, IVPFSEHWA, IVPFSEOWG, and IVPFSEHWG operators. Moreover, we adequately determined and presented the necessary characteristics of our proposed operators.
2. We develop the EDAS technique based on the proposed AOs to solve MAGDM problems. The developed EDAS method is systematically divided into two phases:
3. Data aggregation, facilitated by the execution of IVPFSS Einstein AOs, is the initial phase.
4. The subsequent step is to identify the most effective choice using the EDAS method in the IVPFSS framework.
5. The proposed EDAS technique is used to solve MAGDM problems in the evaluation of optimal suppliers in FSCM and explores the comparative analysis of the developed technique with prevailing models.

## Preliminaries

This section will explore relevant topics such as IVFS, SS, PFSS, IVIFSS, and IVPFSS to lay a strong foundation for the subsequent topic.

### Definition 3.1

Ref.^12^. Suppose the universe of discourse is $U$. The IVFS $\beta$ over $U$ can be defined as:

$$\beta =\left\{\left({\mathfrak{L}}_{i}, {\lambda }_{{\beta }_{j}}\left({\mathfrak{L}}_{i}\right)\right) {\vert}{\mathfrak{L}}_{i}\in U\right\}$$

where, ${\lambda }_{{\beta }_{j}}\left({\mathfrak{L}}_{i}\right)$ be the MD interval, such as ${\lambda }_{{\beta }_{j}}\left({\mathfrak{L}}_{i}\right)=\left[{\lambda }_{{\beta }_{j}}^{l}\left({\mathfrak{L}}_{i}\right), {\lambda }_{{\beta }_{j}}^{\mathpzc{u}}\left({\mathfrak{L}}_{i}\right)\right]$, also, $\left[{\lambda }_{{\beta }_{j}}^{l}\left({\mathfrak{L}}_{i}\right), {\lambda }_{{\beta }_{j}}^{\mathpzc{u}}\left({\mathfrak{L}}_{i}\right)\right]\subseteq \left[0, 1\right]$ and ${\lambda }_{{\mathcal{A}}_{j}}^{l}\left({\mathfrak{L}}_{i}\right), {\lambda }_{{\mathcal{A}}_{j}}^{\mathpzc{u}}\left({\mathfrak{L}}_{i}\right)\in \left[0, 1\right]$.

The conversion between IVFS and IFS will be executed easily.

### Definition 3.2

Ref.^14^. Let $U$ be the domain of discourse, then IVIFS $\beta$ over $U$ can be defined as:

$$\beta =\left\{\left({\mathfrak{L}}_{i}, \left({\lambda }_{{\beta }_{j}}\left({\mathfrak{L}}_{i}\right), {\eta }_{{\beta }_{j}}\left({\mathfrak{L}}_{i}\right)\right)\right) {\vert}{\mathfrak{L}}_{i}\in U\right\}$$

where, ${\lambda }_{{\beta }_{j}}\left({\mathfrak{L}}_{i}\right)=\left[{\lambda }_{{\beta }_{j}}^{l}\left({\mathfrak{L}}_{i}\right), {\lambda }_{{\beta }_{j}}^{\mathpzc{u}}\left({\mathfrak{L}}_{i}\right)\right]$ and ${\eta }_{{\beta }_{j}}\left({\mathfrak{L}}_{i}\right)=\left[{\eta }_{{\beta }_{j}}^{l}\left({\mathfrak{L}}_{i}\right), {\eta }_{{\beta }_{j}}^{\mathpzc{u}}\left({\mathfrak{L}}_{i}\right)\right]$ be the MD and NMD intervals, respectively.

Also, $$\left[{\lambda }_{{\beta }_{j}}^{l}\left({\mathfrak{L}}_{i}\right), {\lambda }_{{\beta }_{j}}^{\mathpzc{u}}\left({\mathfrak{L}}_{i}\right)\right]\subseteq \left[0, 1\right]$$ and

$$\left[{\eta }_{{\beta }_{j}}^{l}\left({\mathfrak{L}}_{i}\right), {\eta }_{{\beta }_{j}}^{\mathpzc{u}}\left({\mathfrak{L}}_{i}\right)\right]\subseteq \left[0, 1\right],$$

$$0\le {\lambda }_{{\beta }_{j}}^{l}\left({\mathfrak{L}}_{i}\right), {\lambda }_{{\beta }_{j}}^{\mathpzc{u}}\left({\mathfrak{L}}_{i}\right), {\eta }_{{\beta }_{j}}^{l}\left({\mathfrak{L}}_{i}\right), {\eta }_{{\beta }_{j}}^{\mathpzc{u}}\left({\mathfrak{L}}_{i}\right)\le 1,$$

such as $$0\le {\lambda }_{{\beta }_{j}}^{\mathpzc{u}}\left({\mathfrak{L}}_{i}\right)+{\eta }_{{\beta }_{j}}^{\mathpzc{u}}\left({\mathfrak{L}}_{i}\right)\le 1$$.

### Definition 3.3

Ref.^16^. Suppose $U$ is the universe of discourse, then IVPFS $\beta$ over $U$ can be defined as:

$$\beta =\left\{\left({\mathfrak{L}}_{i}, \left({\lambda }_{{\beta }_{j}}\left({\mathfrak{L}}_{i}\right), {\eta }_{{\beta }_{j}}\left({\mathfrak{L}}_{i}\right)\right)\right) {\vert}{\mathfrak{L}}_{i}\in U\right\}$$

where, ${\lambda }_{{\beta }_{j}}\left({\mathfrak{L}}_{i}\right)=\left[{\lambda }_{{\beta }_{j}}^{l}\left({\mathfrak{L}}_{i}\right), {\lambda }_{{\beta }_{j}}^{\mathpzc{u}}\left({\mathfrak{L}}_{i}\right)\right]$ and ${\eta }_{{\beta }_{j}}\left({\mathfrak{L}}_{i}\right)=\left[{\eta }_{{\beta }_{j}}^{l}\left({\mathfrak{L}}_{i}\right), {\eta }_{{\beta }_{j}}^{\mathpzc{u}}\left({\mathfrak{L}}_{i}\right)\right]$ be the MD and NMD intervals.

Also, $$\left[{\lambda }_{{\beta }_{j}}^{l}\left({\mathfrak{L}}_{i}\right), {\lambda }_{{\beta }_{j}}^{\mathpzc{u}}\left({\mathfrak{L}}_{i}\right)\right]\subseteq \left[0, 1\right]$$ and

$$\left[{\eta }_{{\beta }_{j}}^{l}\left({\mathfrak{L}}_{i}\right), {\eta }_{{\beta }_{j}}^{\mathpzc{u}}\left({\mathfrak{L}}_{i}\right)\right]\subseteq \left[0, 1\right],$$

$$0\le {\lambda }_{{\beta }_{j}}^{l}\left({\mathfrak{L}}_{i}\right), {\lambda }_{{\beta }_{j}}^{\mathpzc{u}}\left({\mathfrak{L}}_{i}\right), {\eta }_{{\beta }_{j}}^{l}\left({\mathfrak{L}}_{i}\right), {\eta }_{{\beta }_{j}}^{\mathpzc{u}}\left({\mathfrak{L}}_{i}\right)\le 1,$$

such as $$0\le {\left({\lambda }_{{\beta }_{j}}^{\mathpzc{u}}\left({\mathfrak{L}}_{i}\right)\right)}^{2}+{\left({\eta }_{{\beta }_{j}}^{\mathpzc{u}}\left({\mathfrak{L}}_{i}\right)\right)}^{2}\le 1$$.

### Definition 3.4

Ref.^17^. Suppose the universe of discourse is $U,$ and $\mathfrak{R}$ is the set of attributes. A pair $\left(\mathcal{G},\beta \right)$ is a soft set on $U$, where $\mathcal{G}$ is a function defined over $\beta \subseteq \mathfrak{R}$ to $\mathcal{P}(U)$ such as:

$$\mathcal{G}:\beta \to \mathcal{P}(U)$$

Alternatively, it can be expressed as:

$$\left(\mathcal{G},\beta \right)=\left\{\mathcal{G}\left(r\right)\in \mathcal{P}\left(\mathsf{U}\right):r\in \mathfrak{R},\mathcal{G}\left(r\right)= \varnothing if r\notin \beta \right\}$$

### Definition 3.5

Ref.^19^. Suppose the universe of discourse is $U,$ and $\mathfrak{R}$ is the set of attributes. A pair $\left(\mathcal{G},\beta \right)$ is an IVIFSS on $U$, where $\mathcal{G}$ is a function defined over $\beta \subseteq \mathfrak{R}$ to $\mathcal{P}(U)$ such as:

$$\mathcal{G}:\beta \to \mathcal{P}(U)$$

Also, it may be stated as follows:

$$\left(\mathcal{G},\beta \right)=\left\{\left({\mathfrak{L}}_{i}, \left({\lambda }_{{\beta }_{j}}\left({\mathfrak{L}}_{i}\right), {\eta }_{{\beta }_{j}}\left({\mathfrak{L}}_{i}\right)\right)\right) {\vert}{\mathfrak{L}}_{i}\in U\right\}$$

where, ${\lambda }_{{\beta }_{j}}\left({\mathfrak{L}}_{i}\right)=\left[{\lambda }_{{\beta }_{j}}^{l}\left({\mathfrak{L}}_{i}\right), {\lambda }_{{\beta }_{j}}^{\mathpzc{u}}\left({\mathfrak{L}}_{i}\right)\right]$ and ${\eta }_{{\beta }_{j}}\left({\mathfrak{L}}_{i}\right)=\left[{\eta }_{{\beta }_{j}}^{l}\left({\mathfrak{L}}_{i}\right), {\eta }_{{\beta }_{j}}^{\mathpzc{u}}\left({\mathfrak{L}}_{i}\right)\right]$ be the MD and NMD intervals.

Also, $$\left[{\lambda }_{{\beta }_{j}}^{l}\left({\mathfrak{L}}_{i}\right), {\lambda }_{{\beta }_{j}}^{\mathpzc{u}}\left({\mathfrak{L}}_{i}\right)\right]\subseteq \left[0, 1\right]$$ and

$$\left[{\eta }_{{\beta }_{j}}^{l}\left({\mathfrak{L}}_{i}\right), {\eta }_{{\beta }_{j}}^{\mathpzc{u}}\left({\mathfrak{L}}_{i}\right)\right]\subseteq \left[0, 1\right],$$

$$0\le {\lambda }_{{\beta }_{j}}^{l}\left({\mathfrak{L}}_{i}\right), {\lambda }_{{\beta }_{j}}^{\mathpzc{u}}\left({\mathfrak{L}}_{i}\right), {\eta }_{{\beta }_{j}}^{l}\left({\mathfrak{L}}_{i}\right), {\eta }_{{\beta }_{j}}^{\mathpzc{u}}\left({\mathfrak{L}}_{i}\right)\le 1,$$

such as $$0\le {\lambda }_{{\beta }_{j}}^{\mathpzc{u}}\left({\mathfrak{L}}_{i}\right)+{\eta }_{{\beta }_{j}}^{\mathpzc{u}}\left({\mathfrak{L}}_{i}\right)\le 1$$.

Structures like IVIFSS^19^ might not be able to capture this data in cases where the expression is fully ${\lambda }_{{\beta }_{j}}^{\mathpzc{u}}\left({\mathfrak{L}}_{i}\right)+{\eta }_{{\beta }_{j}}^{\mathpzc{u}}\left({\mathfrak{L}}_{i}\right)>1$ is applicable. As a result, an IVPFSS is born, an improved version of IVIFSS that increases productivity by combining the features of IVPFS and SS. An improved, powerful variant of IVPFS, IVPFSS, offers more features and is more efficient overall. This technique facilitates the effective consideration of dispersion and uncertainties, which in turn supports an adequate and secure analysis of challenging data sets. While it discusses DM and analytical estimation, IVPFSS is feasible due to its reliable potential.

### Definition 3.7

Ref.^21^. Assume the universe of discourse is $U$ and $\mathfrak{R}$ is a set of attributes. A pair $\left(\mathcal{G},\beta \right)$ is an IVPFSS on $U$, with $\mathcal{G}$ is a function defined over $\beta \subseteq \mathfrak{R}$ to $\mathcal{P}(U)$ such that:

$$\mathcal{G}:\beta \to \mathcal{P}(U)$$

Also, it may be stated as follows:

$$\left(\mathcal{G},\beta \right)=\left\{\left({\mathfrak{L}}_{i}, \left({\lambda }_{{\beta }_{j}}\left({\mathfrak{L}}_{i}\right), {\eta }_{{\beta }_{j}}\left({\mathfrak{L}}_{i}\right)\right)\right) {\vert}{\mathfrak{L}}_{i}\in U\right\}$$

where, ${\lambda }_{{\beta }_{j}}\left({\mathfrak{L}}_{i}\right)=\left[{\lambda }_{{\beta }_{j}}^{l}\left({\mathfrak{L}}_{i}\right), {\lambda }_{{\beta }_{j}}^{\mathpzc{u}}\left({\mathfrak{L}}_{i}\right)\right]$ and ${\eta }_{{\beta }_{j}}\left({\mathfrak{L}}_{i}\right)=\left[{\eta }_{{\beta }_{j}}^{l}\left({\mathfrak{L}}_{i}\right), {\eta }_{{\beta }_{j}}^{\mathpzc{u}}\left({\mathfrak{L}}_{i}\right)\right]$ be the MD and NMD intervals.

Also, $$\left[{\lambda }_{{\beta }_{j}}^{l}\left({\mathfrak{L}}_{i}\right), {\lambda }_{{\beta }_{j}}^{\mathpzc{u}}\left({\mathfrak{L}}_{i}\right)\right]\subseteq \left[0, 1\right]$$ and

$$\left[{\eta }_{{\beta }_{j}}^{l}\left({\mathfrak{L}}_{i}\right), {\eta }_{{\beta }_{j}}^{\mathpzc{u}}\left({\mathfrak{L}}_{i}\right)\right]\subseteq \left[0, 1\right],$$

$$0\le {\lambda }_{{\beta }_{j}}^{l}\left({\mathfrak{L}}_{i}\right), {\lambda }_{{\beta }_{j}}^{\mathpzc{u}}\left({\mathfrak{L}}_{i}\right), {\eta }_{{\beta }_{j}}^{l}\left({\mathfrak{L}}_{i}\right), {\eta }_{{\beta }_{j}}^{\mathpzc{u}}\left({\mathfrak{L}}_{i}\right)\le 1,$$

such as $$0\le {\left({\lambda }_{{\beta }_{j}}^{\mathpzc{u}}\left({\mathfrak{L}}_{i}\right)\right)}^{2}+{\left({\eta }_{{\beta }_{j}}^{\mathpzc{u}}\left({\mathfrak{L}}_{i}\right)\right)}^{2}\le 1$$.

The score function is given in the following Eq. 1 to evaluate the rank.

1 $$S\left({\mathcal{G}}_{\beta }\right)=\frac{{\left({\lambda }_{{\beta }_{j}}^{l}\left({\mathfrak{L}}_{i}\right)\right)}^{2}+{\left({\lambda }_{{\beta }_{j}}^{\mathpzc{u}}\left({\mathfrak{L}}_{i}\right)\right)}^{2}-{\left({\eta }_{{\beta }_{j}}^{l}\left({\mathfrak{L}}_{i}\right)\right)}^{2}-{\left({\eta }_{{\beta }_{j}}^{\mathpzc{u}}\left({\mathfrak{L}}_{i}\right)\right)}^{2}}{2}$$

When MD and NMD intervals are equal, a score will no longer be effective in choosing the most beneficial option as defined above. For this, we suggest a more extensive scoring mechanism for IVPFSNs, known as an accuracy function, given by the following Eq. 2.

2 $$H\left({\mathcal{G}}_{\beta }\right)=\frac{{\left({\lambda }_{{\beta }_{j}}^{l}\left({\mathfrak{L}}_{i}\right)\right)}^{2}+{\left({\lambda }_{{\beta }_{j}}^{\mathpzc{u}}\left({\mathfrak{L}}_{i}\right)\right)}^{2}+{\left({\eta }_{{\beta }_{j}}^{l}\left({\mathfrak{L}}_{i}\right)\right)}^{2}+{\left({\eta }_{{\beta }_{j}}^{\mathpzc{u}}\left({\mathfrak{L}}_{i}\right)\right)}^{2}}{2}$$

For the reader’s comfort, the IVPFSN ${\mathcal{G}}_{{\beta }_{ij}}=\left\{\left({\lambda }_{{\beta }_{ij}}\left({\mathfrak{L}}_{i}\right), {\eta }_{{\beta }_{ij}}\left({\mathfrak{L}}_{i}\right)\right)|{\mathfrak{L}}_{i}\in U\right\}$ can be described as ${\mathcal{G}}_{{\beta }_{ij}}=\left({\lambda }_{{\beta }_{ij}}, {\eta }_{{\beta }_{ij}}\right)$.

We will now present the operational laws that are identified below, taking into consideration this context:

### Definition 3.9

Ref.^21^. Let ${\Delta }_{a}=\left(\left[{\lambda }_{a}^{l}, {\lambda }_{a}^{\mathpzc{u}}\right], \left[{\eta }_{a}^{l}, {\eta }_{a}^{\mathpzc{u}}\right]\right)$, ${\Delta }_{{a}_{11}}=\left(\left[{\lambda }_{{a}_{11}}^{l}, {\lambda }_{{a}_{11}}^{\mathpzc{u}}\right], \left[{\eta }_{{a}_{11}}^{l}, {\eta }_{{a}_{11}}^{\mathpzc{u}}\right]\right)$, and ${\Delta }_{{a}_{12}}=\left(\left[{\lambda }_{{a}_{12}}^{l}, {\lambda }_{{a}_{12}}^{\mathpzc{u}}\right], \left[{\eta }_{{a}_{12}}^{l}, {\eta }_{{a}_{12}}^{\mathpzc{u}}\right]\right)$ are IVPFSNs and $\theta >0$. Thus, the operational laws for the IVPFSNs defined in^21^ are presented as follows:

$${\Delta }_{{a}_{11}}\oplus {\Delta }_{{a}_{12}}$$

$$=\left(\begin{array}{c}\left[\sqrt{{\left({\lambda }_{{a}_{11}}^{l}\right)}^{2}+{\left({\lambda }_{{a}_{12}}^{l}\right)}^{2}-{\left({\lambda }_{{a}_{11}}^{l}\right)}^{2}{\left({\lambda }_{{a}_{12}}^{l}\right)}^{2}},\sqrt{{\left({\lambda }_{{a}_{11}}^{\mathpzc{u}}\right)}^{2}+{\left({\lambda }_{{a}_{12}}^{\mathpzc{u}}\right)}^{2}-{\left({\lambda }_{{a}_{11}}^{\mathpzc{u}}\right)}^{2}{\left({\lambda }_{{a}_{12}}^{\mathpzc{u}}\right)}^{2}}\right]\\ ,\left[{\eta }_{{a}_{11}}^{l}{\eta }_{{a}_{12}}^{l}, {\eta }_{{a}_{11}}^{\mathpzc{u}}{\eta }_{{a}_{12}}^{\mathpzc{u}}\right]\end{array}\right)$$

$${\Delta }_{{a}_{11}}\otimes {\Delta }_{{a}_{12}}$$

$$=\left(\left[{\lambda }_{{a}_{11}}^{l}{\lambda }_{{a}_{12}}^{l},{\lambda }_{{a}_{11}}^{\mathpzc{u}}{\lambda }_{{a}_{12}}^{\mathpzc{u}}\right],\left[\begin{array}{c}\sqrt{{\left({\eta }_{{a}_{11}}^{l}\right)}^{2}+{\left({\eta }_{{a}_{12}}^{l}\right)}^{2}-{\left({\eta }_{{a}_{11}}^{l}\right)}^{2}{\left({\eta }_{{a}_{12}}^{l}\right)}^{2}},\\ \sqrt{{\left({\eta }_{{a}_{11}}^{\mathpzc{u}}\right)}^{2}+{\left({\eta }_{{a}_{12}}^{\mathpzc{u}}\right)}^{2}-{\left({\eta }_{{a}_{11}}^{\mathpzc{u}}\right)}^{2}{\left({\eta }_{{a}_{12}}^{\mathpzc{u}}\right)}^{2}}\end{array}\right]\right)$$

$$\theta {\Delta }_{a}=\left(\left[\sqrt{1-{\left(1-{\left({\lambda }_{a}^{l}\right)}^{2}\right)}^{\theta }},\sqrt{1-{\left(1-{\left({\lambda }_{a}^{\mathpzc{u}}\right)}^{2}\right)}^{\theta }} \right],\left[{\left({\eta }_{a}^{l}\right)}^{\theta },{\left({\eta }_{a}^{\mathpzc{u}}\right)}^{\theta }\right]\right)$$

$$=\left(\sqrt{1-{\left(1-{\left[{\lambda }_{a}^{l},{\lambda }_{a}^{\mathpzc{u}}\right]}^{2}\right)}^{\theta }},\left[{\left({\eta }_{a}^{l}\right)}^{\theta },{\left({\eta }_{a}^{\mathpzc{u}}\right)}^{\theta }\right]\right)$$

$${\Delta }_{a}^{\theta }=\left(\left[{\left({\lambda }_{a}^{l}\right)}^{\theta },{\left({\lambda }_{a}^{\mathpzc{u}}\right)}^{\theta }\right],\left[\sqrt{1-{\left(1-{\left({\eta }_{a}^{l}\right)}^{2}\right)}^{\theta }}, \sqrt{1-{\left(1-{\left({\eta }_{a}^{\mathpzc{u}}\right)}^{2}\right)}^{\theta }}\right]\right)$$

$$=\left(\left[{\left({\lambda }_{a}^{l}\right)}^{\theta },{\left({\lambda }_{a}^{\mathpzc{u}}\right)}^{\theta }\right],\sqrt{1-{\left(1-{\left[{\eta }_{a}^{l},{\eta }_{a}^{\mathpzc{u}}\right]}^{2}\right)}^{\theta }}\right)$$

Zulqarnain et al.^21^ presented the AOs with their basic properties for IVPFSS using the above-stated operational laws.

$$IVPFSWA\left({\Delta }_{{a}_{11}},{\Delta }_{{a}_{12}},\dots \dots \dots ,{\Delta }_{{a}_{nm}}\right)$$

$$=\left(\left[\sqrt{1-\prod_{j=1}^{m}{\left(\prod_{i=1}^{n}{\left(1-{\left({\lambda }_{{a}_{ij}}^{l}\right)}^{2}\right)}^{{\partial }_{i}}\right)}^{{\Upsilon}_{j}}}, \sqrt{1-\prod_{j=1}^{m}{\left(\prod_{i=1}^{n}{\left(1-{\left({\lambda }_{{a}_{ij}}^{\mathpzc{u}}\right)}^{2}\right)}^{{\partial }_{i}}\right)}^{{\Upsilon}_{j}}}\right],\left[\begin{array}{c}\prod_{j=1}^{m}{\left(\prod_{i=1}^{n}{\left({\left({\eta }_{{a}_{ij}}^{l}\right)}^{2}\right)}^{{\partial }_{i}}\right)}^{{\Upsilon}_{j}}\\ , \prod_{j=1}^{m}{\left(\prod_{i=1}^{n}{\left({\left({\eta }_{{a}_{ij}}^{\mathpzc{u}}\right)}^{2}\right)}^{{\partial }_{i}}\right)}^{{\Upsilon}_{j}}\end{array}\right]\right)$$

$$IVPFSWG\left({\Delta }_{{a}_{11}},{\Delta }_{{a}_{12}},\dots \dots \dots ,{\Delta }_{{a}_{nm}}\right)$$

$$=\left(\left[\prod_{j=1}^{m}{\left(\prod_{i=1}^{n}{\left({\left({\lambda }_{{a}_{ij}}^{l}\right)}^{2}\right)}^{{\partial }_{i}}\right)}^{{\Upsilon }_{j}}, \prod_{j=1}^{m}{\left(\prod_{i=1}^{n}{\left({\left({\lambda }_{{a}_{ij}}^{\mathpzc{u}}\right)}^{2}\right)}^{{\partial }_{i}}\right)}^{{\Upsilon}_{j}}\right],\left[\begin{array}{c}\sqrt{1-\prod_{j=1}^{m}{\left(\prod_{i=1}^{n}{\left(1-{\left({\eta }_{{a}_{ij}}^{l}\right)}^{2}\right)}^{{\partial }_{i}}\right)}^{{\Upsilon}_{j}}}\\ , \sqrt{1-\prod_{j=1}^{m}{\left(\prod_{i=1}^{n}{\left(1-{\left({\eta }_{{a}_{ij}}^{\mathpzc{u}}\right)}^{2}\right)}^{{\partial }_{i}}\right)}^{{\Upsilon}_{j}}}\end{array}\right]\right)$$

## Interval-valued Pythagorean fuzzy soft Einstein weighted average aggregation operators

We thoroughly explore the IVPFSS by integrating Einstein’s operational rules into this topic. We have a framework for handling the vast amount of IVPFS information generated by these rules. We formulate and outline Einstein averaging AOs for IVPFSVs based on these fundamental notions. These operators are beneficial in boosting and accelerating the aggregation method.

### Definition 4.1.

Let $${\Delta }_{{a}_{{\varsigma}}}=\left(\left[{\lambda }_{{a}_{{\varsigma}}}^{l},{\uplambda }_{{a}_{{\varsigma}}}^{\mathpzc{u}}\right],\left[{\eta }_{{a}_{{\varsigma}}}^{l},{\eta }_{{a}_{{\varsigma}}}^{\mathpzc{u}}\right]\right),{\Delta }_{{\mathrm{a}}_{11}}=\left(\left[{\lambda }_{{a}_{11}}^{l},{\lambda }_{{a}_{11}}^{\mathpzc{u}}\right],\left[{\eta }_{{a}_{11}}^{l},{\eta }_{{a}_{11}}^{\mathpzc{u}}\right]\right)$$    

and $${\Delta }_{{\mathrm{a}}_{12}}=\left(\left[{\lambda }_{{a}_{12}}^{l},{\eta }_{{a}_{12}}^{\mathpzc{u}}\right],\left[{\lambda }_{{a}_{12}}^{l},{\eta }_{{a}_{12}}^{\mathpzc{u}}\right]\right)$$

 be three IVPFSVs, and $\theta >0$ be any real number. Then,

  

$${\Delta }_{{a}_{11}}{\oplus }_{\varepsilon }{\Delta }_{{a}_{12}}$$

$$=\left(\begin{array}{c}\left[\begin{array}{c}\frac{\sqrt{{\left({\lambda }_{{a}_{11}}^{l}\right)}^{2}+{\left({\lambda }_{{a}_{12}}^{l}\right)}^{2}}}{\sqrt{1+{\left({\lambda }_{{a}_{11}}^{l}\right)}^{2}{\left({\lambda }_{{a}_{12}}^{l}\right)}^{2}}},\frac{\sqrt{{\left({\lambda }_{{a}_{11}}^{\mathpzc{u}}\right)}^{2}+{\left({\lambda }_{{a}_{12}}^{\mathpzc{u}}\right)}^{2}}}{\sqrt{1+{\left({\lambda }_{{a}_{11}}^{\mathpzc{u}}\right)}^{2}{\left({\lambda }_{{a}_{12}}^{\mathpzc{u}}\right)}^{2}}}\end{array}\right]\\ ,\left[\begin{array}{c}\frac{\left({\eta }_{{a}_{11}}^{l}\right)\left({\eta }_{{a}_{12}}^{l}\right)}{\sqrt{1+\left(1-{\left({\eta }_{{a}_{11}}^{l}\right)}^{2}\right)\left(1-{\left({\eta }_{{a}_{12}}^{l}\right)}^{2}\right)}},\frac{\left({\eta }_{{a}_{11}}^{\mathpzc{u}}\right)\left({\eta }_{{a}_{12}}^{\mathpzc{u}}\right)}{\sqrt{1+\left(1-{\left({\eta }_{{a}_{11}}^{\mathpzc{u}}\right)}^{2}\right)\left(1-{\left({\eta }_{{a}_{12}}^{\mathpzc{u}}\right)}^{2}\right)}}\end{array}\right]\end{array}\right)$$

$${\Delta }_{{a}_{11}}{{\otimes}}_{\varepsilon }{\Delta }_{{a}_{12}}$$

$$=\left(\begin{array}{c}\left[\begin{array}{c}\frac{\left({\lambda }_{{a}_{11}}^{l}\right)\left({\lambda }_{{a}_{12}}^{l}\right)}{\sqrt{1+\left(1-{\left({\lambda }_{{a}_{11}}^{l}\right)}^{2}\right)\left(1-{\left({\lambda }_{{a}_{12}}^{l}\right)}^{2}\right)}},\frac{\left({\lambda }_{{a}_{11}}^{\mathpzc{u}}\right)\left({\lambda }_{{a}_{12}}^{\mathpzc{u}}\right)}{\sqrt{1+\left(1-{\left({\lambda }_{{a}_{11}}^{\mathpzc{u}}\right)}^{2}\right)\left(1-{\left({\lambda }_{{a}_{12}}^{\mathpzc{u}}\right)}^{2}\right)}}\end{array}\right]\\ ,\left[\begin{array}{c}\frac{\sqrt{{\left({\eta }_{{a}_{11}}^{l}\right)}^{2}+{\left({\eta }_{{a}_{12}}^{l}\right)}^{2}}}{\sqrt{1+{\left({\eta }_{{a}_{11}}^{l}\right)}^{2}{\left({\eta }_{{a}_{12}}^{l}\right)}^{2}}},\frac{\sqrt{{\left({\eta }_{{a}_{11}}^{\mathpzc{u}}\right)}^{2}+{\left({\eta }_{{a}_{12}}^{\mathpzc{u}}\right)}^{2}}}{\sqrt{1+{\left({\eta }_{{a}_{11}}^{\mathpzc{u}}\right)}^{2}{\left({\eta }_{{a}_{12}}^{\mathpzc{u}}\right)}^{2}}}\end{array}\right]\end{array}\right)$$

$${\Delta }_{{a}_{{\varsigma}}}^{\theta }$$

$$=\left(\begin{array}{c}\left[\begin{array}{c}\frac{\sqrt{2{\left({\left({\lambda }_{{a}_{{\varsigma}}}^{l}\right)}^{2}\right)}^{\theta }}}{\sqrt{{\left(2-{\left({\lambda }_{{a}_{{\varsigma}}}^{l}\right)}^{2}\right)}^{\theta }+{\left({\left({\lambda }_{{a}_{{\varsigma}}}^{l}\right)}^{2}\right)}^{\theta }}},\frac{\sqrt{2{\left({\left({\eta }_{{a}_{{\varsigma}}}^{\mathpzc{u}}\right)}^{2}\right)}^{\theta }}}{\sqrt{{\left(2-{\left({\eta }_{{a}_{{\varsigma}}}^{\mathpzc{u}}\right)}^{2}\right)}^{\theta }+{\left({\left({\eta }_{{a}_{{\varsigma}}}^{\mathpzc{u}}\right)}^{2}\right)}^{\theta }}}\end{array}\right]\\ ,\left[\begin{array}{c}\frac{\sqrt{{\left(1+{\left({\eta }_{{a}_{{\varsigma}}}^{l}\right)}^{2}\right)}^{\theta }-{\left(1-{\left({\eta }_{{a}_{{\varsigma}}}^{l}\right)}^{2}\right)}^{\theta }}}{\sqrt{{\left(1+{\left({\eta }_{{a}_{{\varsigma}}}^{l}\right)}^{2}\right)}^{\theta }+{\left(1-{\left({\eta }_{{a}_{{\varsigma}}}^{l}\right)}^{2}\right)}^{\theta }}},\frac{\sqrt{{\left(1+{\left({\eta }_{{a}_{{\varsigma}}}^{\mathpzc{u}}\right)}^{2}\right)}^{\theta }-{\left(1-{\left({\eta }_{{a}_{{\varsigma}}}^{\mathpzc{u}}\right)}^{2}\right)}^{\theta }}}{\sqrt{{\left(1+{\left({\eta }_{{a}_{{\varsigma}}}^{\mathpzc{u}}\right)}^{2}\right)}^{\theta }+{\left(1-{\left({\eta }_{{a}_{{\varsigma}}}^{\mathpzc{u}}\right)}^{2}\right)}^{\theta }}}\end{array}\right]\end{array}\right)$$

$$\theta {\Delta }_{{a}_{{\varsigma}}}$$

$$=\left(\begin{array}{c}\left[\begin{array}{c}\frac{\sqrt{{\left(1+{\left({\lambda }_{{a}_{{\varsigma}}}^{l}\right)}^{2}\right)}^{\theta }-{\left(1-{\left({\lambda }_{{a}_{{\varsigma}}}^{l}\right)}^{2}\right)}^{\theta }}}{\sqrt{{\left(1+{\left({\lambda }_{{a}_{{\varsigma}}}^{l}\right)}^{2}\right)}^{\theta }+{\left(1-{\left({\lambda }_{{a}_{{\varsigma}}}^{l}\right)}^{2}\right)}^{\theta }}},\frac{\sqrt{{\left(1+{\left({\lambda }_{{a}_{{\varsigma}}}^{\mathpzc{u}}\right)}^{2}\right)}^{\theta }-{\left(1-{\left({\lambda }_{{a}_{{\varsigma}}}^{\mathpzc{u}}\right)}^{2}\right)}^{\theta }}}{\sqrt{{\left(1+{\left({\lambda }_{{a}_{{\varsigma}}}^{\mathpzc{u}}\right)}^{2}\right)}^{\theta }+{\left(1-{\left({\lambda }_{{a}_{{\varsigma}}}^{\mathpzc{u}}\right)}^{2}\right)}^{\theta }}}\end{array}\right]\\ ,\left[\begin{array}{c}\frac{\sqrt{{\left({\left({\eta }_{{a}_{{\varsigma}}}^{l}\right)}^{2}\right)}^{\theta }}}{\sqrt{{\left(2-{\left({\eta }_{{a}_{{\varsigma}}}^{l}\right)}^{2}\right)}^{\theta }+{\left({\left({\eta }_{{a}_{{\varsigma}}}^{l}\right)}^{2}\right)}^{\theta }}},\frac{\sqrt{{\left({\left({\eta }_{{a}_{{\varsigma}}}^{\mathpzc{u}}\right)}^{2}\right)}^{\theta }}}{\sqrt{{\left(2-{\left({\eta }_{{a}_{{\varsigma}}}^{\mathpzc{u}}\right)}^{2}\right)}^{\theta }+{\left({\left({\lambda }_{{a}_{{\varsigma}}}^{\mathpzc{u}}\right)}^{2}\right)}^{\theta }}}\end{array}\right]\end{array}\right)$$

### Definition 4.2.

Let ${\Delta }_{{a}_{ij}}=\left(\left[{\lambda }_{{a}_{ij}}^{l},{\lambda }_{{a}_{ij}}^{\mathpzc{u}}\right],\left[{\eta }_{{a}_{ij}}^{l},{\eta }_{{a}_{ij}}^{\mathpzc{u}}\right]\right)$ be a collection of IVPFSVs. Then, IVPFSEOWA is defined as:

$$IVPFSEOWA\left({\Delta }_{{a}_{11}},{\Delta }_{{a}_{12}},\dots \dots ,{\Delta }_{{a}_{nm}}\right)$$

$$={\Upsilon}_{1}{{\partial }_{1}\Delta }_{p\left({a}_{{t^{\prime}}\left(1\right)z\left(1\right)}\right)}{\oplus }_{\varepsilon }{\Upsilon}_{1}{{\partial }_{2}\Delta }_{p\left({a}_{{t^{\prime}}\left(2\right)z\left(1\right)}\right)}{\oplus }_{\varepsilon }\dots \dots {\oplus }_{\varepsilon }{\Upsilon}_{m}{\partial }_{n}{\Delta }_{p\left({a}_{{t^{\prime}}\left(n\right)z\left(m\right)}\right)}\left(\stackrel{m}{\underset{j=1}{{\oplus }_{\varepsilon }}}{\Upsilon}_{j}\left(\stackrel{n}{\underset{i=1}{{\oplus }_{\varepsilon }}}{\partial }_{i}{\Delta }_{p\left({a}_{{t^{\prime}}\left(i\right) z\left(j\right)}\right)}\right)\right)$$

where, ${\partial }_{i}$ be the weight of experts and ${\Upsilon}_{j}$ be the criteria weights such as ${\partial }_{i}>0,\sum_{i=1}^{n}{\partial }_{i}>0$ and ${\Upsilon}_{j}>0,\sum_{j=1}^{m}{\Upsilon}_{j}=1$. Also, $p\left({a}_{{t^{\prime}}\left(i\right)z\left(j\right)}\right)$ be the permutation, such as ${\Delta }_{p\left({a}_{{t^{\prime}}\left(i\right) z\left(j\right)}\right)}\le {\Delta }_{p\left({a}_{{t^{\prime}}\left(i-1\right) z\left(j\right)}\right)}$ and ${\Delta }_{p\left({a}_{{t^{\prime}}\left(i\right) z\left(j\right)}\right)}\le {\Delta }_{p\left({a}_{{t^{\prime}}\left(i\right) z\left(j-1\right)}\right)}$ $\forall$ $i, j$.

### Theorem 4.3.

Let $\Delta { }_{{a}_{ij}}=\left(\left[{\lambda }_{{a}_{ij}}^{l},{\lambda }_{{a}_{ij}}^{\mathpzc{u}}\right],\left[{\eta }_{{a}_{ij}}^{l},{\eta }_{{a}_{ij}}^{\mathpzc{u}}\right]\right)$ be a collection of IVPFSVs. Then, the aggregated outcome of the IVPFSEOWA operator is also an IVPFSV.

$$IVPFSEOWA\left({\Delta }_{{a}_{11}},{\Delta }_{{a}_{12}},\dots \dots {\Delta }_{{a}_{nm}}\right)$$

3 $$=\left(\begin{array}{c}\left[\begin{array}{c}\frac{\sqrt{\prod_{j=1}^{m}{\left(\prod_{i=1}^{n}{\left(1+{\left({\lambda }_{p\left({a}_{{t^{\prime}}\left(i\right)z\left(j\right)}\right)}^{l}\right)}^{2}\right)}^{{\partial }_{i}}\right)}^{{\Upsilon}_{j}}-\prod_{j=1}^{m}{\left(\prod_{i=1}^{n}{\left(1-{\left({\lambda }_{p\left({a}_{{t^{\prime}}\left(i\right)z\left(j\right)}\right)}^{l}\right)}^{2}\right)}^{{\partial }_{i}}\right)}^{{\Upsilon}_{j}}}}{\sqrt{\prod_{j=1}^{m}{\left(\prod_{i=1}^{n}{\left(1+{\left({\lambda }_{p\left({a}_{{t^{\prime}}\left(i\right)z\left(j\right)}\right)}^{l}\right)}^{2}\right)}^{{\partial }_{i}}\right)}^{{\Upsilon}_{j}}+\prod_{j=1}^{m}{\left(\prod_{i=1}^{n}{\left(1-{\left({\lambda }_{p\left({a}_{{t^{\prime}}\left(i\right)z\left(j\right)}\right)}^{l}\right)}^{2}\right)}^{{\partial }_{i}}\right)}^{{\Upsilon}_{j}}}},\\ \frac{\sqrt{\prod_{j=1}^{m}{\left(\prod_{i=1}^{n}{\left(1+{\left({\lambda }_{p\left({a}_{{t^{\prime}}\left(i\right)z\left(j\right)}\right)}^{\mathpzc{u}}\right)}^{2}\right)}^{{\partial }_{i}}\right)}^{{\Upsilon}_{j}}-\prod_{j=1}^{m}{\left(\prod_{i=1}^{n}{\left(1+{\left({\lambda }_{p\left({a}_{{t^{\prime}}\left(i\right)z\left(j\right)}\right)}^{\mathpzc{u}}\right)}^{2}\right)}^{{\partial }_{i}}\right)}^{{\Upsilon}_{j}}}}{\sqrt{\prod_{j=1}^{m}{\left(\prod_{i=1}^{n}{\left(1+{\left({\lambda }_{p\left({a}_{{t^{\prime}}\left(i\right)z\left(j\right)}\right)}^{\mathpzc{u}}\right)}^{2}\right)}^{{\partial }_{i}}\right)}^{{\Upsilon}_{j}}+\prod_{j=1}^{m}{\left(\prod_{i=1}^{n}{\left(1+{\left({\lambda }_{p\left({a}_{{t^{\prime}}\left(i\right)z\left(j\right)}\right)}^{\mathpzc{u}}\right)}^{2}\right)}^{{\partial }_{i}}\right)}^{{\Upsilon}_{j}}}}\end{array}\right]\\ , \left[\begin{array}{c}\frac{\sqrt{2\prod_{j=1}^{m}{\left(\prod_{i=1}^{n}{\left({\left({\eta }_{p\left({a}_{{t^{\prime}}\left(i\right)z\left(j\right)}\right)}^{l}\right)}^{2}\right)}^{{\partial }_{i}}\right)}^{{\Upsilon}_{j}}}}{\sqrt{\prod_{j=1}^{m}{\left(\prod_{i=1}^{n}{\left(2-{\left({\eta }_{p\left({a}_{{t^{\prime}}\left(i\right)z\left(j\right)}\right)}^{l}\right)}^{2}\right)}^{{\partial }_{i}}\right)}^{{\Upsilon}_{j}}+\prod_{j=1}^{m}{\left(\prod_{i=1}^{n}{\left({\left({\eta }_{p\left({a}_{{t^{\prime}}\left(i\right)z\left(j\right)}\right)}^{l}\right)}^{2}\right)}^{{\partial }_{i}}\right)}^{{\Upsilon}_{j}}}},\\ \frac{\sqrt{2\prod_{j=1}^{m}{\left(\prod_{i=1}^{n}{\left({\left({\eta }_{p\left({a}_{{t^{\prime}}\left(i\right)z\left(j\right)}\right)}^{\mathpzc{u}}\right)}^{2}\right)}^{{\partial }_{i}}\right)}^{{\Upsilon}_{j}}}}{\sqrt{\prod_{j=1}^{m}{\left(\prod_{i=1}^{n}{\left(2-{\left({\eta }_{p\left({a}_{{t^{\prime}}\left(i\right)z\left(j\right)}\right)}^{\mathpzc{u}}\right)}^{2}\right)}^{{\partial }_{i}}\right)}^{{\Upsilon}_{j}}+\prod_{j=1}^{m}{\left(\prod_{i=1}^{n}{\left({\left({\eta }_{p\left({a}_{{t^{\prime}}\left(i\right)z\left(j\right)}\right)}^{\mathpzc{u}}\right)}^{2}\right)}^{{\partial }_{i}}\right)}^{{\Upsilon}_{j}}}}\end{array}\right]\end{array}\right)$$

where ${\partial }_{i}={\left\{{\partial }_{1}, {\partial }_{2}, \dots \dots ,{\partial }_{n}\right\}}^{T}$ are the weights of professionals and ${\Upsilon}_{j}={\left\{{\Upsilon}_{1}, {\Upsilon}_{2}, \dots \dots ,{\Upsilon}_{j}\right\}}^{T}$ are the weights of the criteria with ${\partial }_{i}>0 \mathrm{and}\sum_{i=1}^{n}{\partial }_{i}=1$, and ${\Upsilon}_{j}>0 \mathrm{and} \sum_{j=1}^{m}{\Upsilon}_{j}=1$. Also, ${\Delta }_{\sigma \left({a}_{{t^{\prime}}\left(i\right)z\left(j\right)}\right)}$ be the leading component of ${i}^{th}$ row and ${j}^{th}$ column in $\left({\Delta }_{{a}_{11}},{\Delta }_{{a}_{12}},\dots \dots ,{\Delta }_{{a}_{nm}}\right)$, such as ${\Delta }_{p\left({a}_{{t^{\prime}}\left(i\right)z\left(j\right)}\right)}\le {\Delta }_{p\left({a}_{{t^{\prime}}\left(i-1\right)z\left(j\right)}\right)}$ and ${\Delta }_{p\left({a}_{{t^{\prime}}\left(i\right)z\left(j\right)}\right)}\le {\Delta }_{p\left({a}_{{t^{\prime}}\left(i\right)z\left(j-1\right)}\right)}$ $\forall i,j$.

### Proof:

See Appendix 1.

### Proposition 4.4.

If $\Delta { }_{{a}_{ij}}=\left(\left[{\lambda }_{{a}_{ij}}^{l},{\lambda }_{{a}_{ij}}^{\mathpzc{u}}\right],\left[{\eta }_{{a}_{ij}}^{l},{\eta }_{{a}_{ij}}^{\mathpzc{u}}\right]\right)$ consists of IVPFSVs. Also, ${\partial }_{i}$ be the weights of specialists and ${\Upsilon}_{j}$ are the weights of the criteria, with ${\partial }_{i}>0,\sum_{i=1}^{n}{\partial }_{i}=1$ and ${\Upsilon}_{j}>0,\sum_{j=1}^{m}{\Upsilon}_{j}=1$.

### Idempotency

Let $\Delta { }_{{a}_{ij}}=\Delta { }_{{a}_{o}}=\left(\left[{{\lambda }_{{a}_{o}}}_{ }^{l},{\lambda }_{{a}_{o}}^{\mathpzc{u}}\right],\left[{\eta }_{{a}_{o}}^{l},{\eta }_{{a}_{o}}^{\mathpzc{u}}\right]\right)$ holds. Then,

$$IVPFSEOWA\left({\Delta }_{{a}_{11}},{\Delta }_{{a}_{12}},\dots \dots {\Delta }_{{a}_{nm}}\right)=\Delta { }_{{a}_{o}}$$

### Boundedness

$${\Delta }_{{a}_{ij}}^{-}=\left(\left[min\left({\lambda }_{{a}_{ij} }^{l}\right), min\left({\lambda }_{{a}_{ij}}^{\mathpzc{u}}\right)\right], \left[max\left({\eta }_{{a}_{ij}}^{l}\right), max\left({\eta }_{{a}_{ij}}^{\mathpzc{u}}\right)\right]\right)$$

and $${\Delta }_{{a}_{ij}}^{+}=\left(\left[max\left({\lambda }_{{a}_{ij} }^{l}\right), max\left({\lambda }_{{a}_{ij}}^{\mathpzc{u}}\right)\right], \left[min\left({\eta }_{{a}_{ij}}^{l}\right), min\left({\eta }_{{a}_{ij}}^{\mathpzc{u}}\right)\right]\right)$$. Then

$${\Delta }_{{a}_{ij}}^{-}\le IVPFSEOWA\left({\Delta }_{{a}_{11}},{\Delta }_{{a}_{12}},\dots \dots {\Delta }_{{a}_{nm}}\right)\le {\Delta }_{{a}_{ij}}^{+}$$

### Monotonicity

Let $\Delta { }_{{a}_{ij}}=\left(\left[{\lambda }_{{a}_{ij} }^{l},{\lambda }_{{a}_{ij}}^{\mathpzc{u}}\right],\left[{\eta }_{{a}_{ij}}^{l},{\eta }_{{a}_{ij}}^{\mathpzc{u}}\right]\right) and {\Delta }_{{a}_{ij}}^{*} =\left(\left[{{\lambda }^{*}}_{{a}_{ij} }^{l},{{\lambda }^{*}}_{{a}_{ij}}^{\mathpzc{u}}\right],\left[{{\eta }^{*}}_{{a}_{ij}}^{l},{{\eta }^{*}}_{{a}_{ij}}^{\mathpzc{u}}\right]\right)$ be the families of IVPFSVs. Then,

$$IVPFSEOWA\left({\Delta }_{{a}_{11}},{\Delta }_{{a}_{12}},\dots \dots {\Delta }_{{a}_{nm}}\right)\le IVPFSEOWA \left({\Delta }_{{a}_{11}}^{*},{\Delta }_{{a}_{12}}^{*},\dots \dots \dots , {\Delta }_{{a}_{nm}}^{*}\right)$$

if $${\Delta }_{{a}_{nm}}\le {\Delta }_{{a}_{nm}}^{*}$$ $$\forall i, j$$.

### Homogeneity

Let $\Delta { }_{{a}_{ij}}=\left(\left[{\lambda }_{{a}_{ij} }^{l},{\lambda }_{{a}_{ij}}^{\mathpzc{u}}\right],\left[{\eta }_{{a}_{ij}}^{l},{\eta }_{{a}_{ij}}^{\mathpzc{u}}\right]\right)$ be a family of IVPFSVs. Then,

$$IVPFSEOWA\left(\theta {\Delta }_{{a}_{11}},\theta {\Delta }_{{a}_{12}},\dots \dots \theta {\Delta }_{{a}_{nm}}\right)=\theta IVPFSEOWA\left({\Delta }_{{a}_{11}},{\Delta }_{{a}_{12}},\dots \dots {\Delta }_{{a}_{nm}}\right)$$

where $$\theta >0.$$

### Shift Invariance

Let $\Delta { }_{{a}_{ij}}=\left(\left[{\lambda }_{{a}_{ij} }^{l},{\lambda }_{{a}_{ij}}^{\mathpzc{u}}\right],\left[{\eta }_{{a}_{ij}}^{l},{\eta }_{{a}_{ij}}^{\mathpzc{u}}\right]\right)$ be a collection of IVPFSVs and $\Delta { }_{a}=\left(\left[{\lambda }_{a }^{l},{\lambda }_{a}^{\mathpzc{u}}\right],\left[{\eta }_{a}^{l},{\eta }_{a}^{\mathpzc{u}}\right]\right)$ be an IVPFSV. Then,

$$IVPFSEOWA \left(\Delta { }_{{a}_{11}}{\oplus }_{\varepsilon }\Delta { }_{a},\Delta { }_{{a}_{12}}{\oplus }_{\varepsilon }\Delta { }_{a},\dots ,\Delta { }_{{a}_{nm}}{\oplus }_{\varepsilon }\Delta { }_{a}\right)$$

$$= IVPFSEOWA\left({\Delta }_{{a}_{11}},{\Delta }_{{a}_{12}},\dots \dots {\Delta }_{{a}_{nm}}\right){\oplus }_{\varepsilon }\Delta { }_{a}$$

#### Proof:

Proof is obvious.

#### Remark 4.1.

It is important to consider the subsequent information while investigating the interactions among various ideas:

If ${\left({\lambda }_{ij}^{\mathpzc{u}}\right)}^{2}+{\left({\eta }_{ij}^{\mathpzc{u}}\right)}^{2}\le 1$, then the IVPFSS reduces to an IVIFSS^19^.

If there exists a single attribute, then the IVPFSS reduces to an IVPFS^16^.

Expert opinions and the assignment of importance weights are two key aspects that can be smoothly integrated using hybrid operators. The most crucial purpose of the Einstein-ordered AOs is to collect factors in a predetermined instruction. This technique is beneficial for real-world applications, where judgment entries often originate from multiple sources and opinions. If implementing both aspects is necessary, a more efficient and dynamic aggregate technique can be achieved by using Einstein hybrid aggregation operators. These operators focus entirely on one subset of these factors. We’ll discuss the IVPFSEHWA operator below to overcome this restriction.

#### Definition 4.5.

Let ${\Delta }_{{a}_{ij}}^{\prime} =\left(\left[{{\lambda }^{\prime}}_{{a}_{ij}}^{l},{{\lambda }^{\prime}}_{{a}_{ij}}^{\mathpzc{u}}\right],\left[{{\eta }^{\prime}}_{{a}_{ij}}^{l},{{\eta }^{\prime}}_{{a}_{ij}}^{\mathpzc{u}}\right]\right)$ be a family of IVPFSVs. Then, IVPFSEHWA is defined as:

$$IVPFSEHWA\left({\Delta }_{{a}_{11}}^{\prime},{\Delta }_{{a}_{12}}^{{{\prime}}},\dots \dots ,{\Delta }_{{a}_{nm}}^{\prime}\right)=\left(\stackrel{m}{\underset{j=1}{{\oplus }_{\varepsilon }}}{\Upsilon}_{j}\left(\stackrel{n}{\underset{i=1}{{\oplus }_{\varepsilon }}}{\partial }_{i}{\Delta }_{p\left({a}_{{t^{\prime}}\left(i\right)z\left(j\right)}\right)}^{\prime}\right)\right)$$

where ${\partial }_{i}$ be the weights of specialists and ${\Upsilon}_{j}$ are the weights of the criteria, with ${\partial }_{i}>0,\sum_{i=1}^{n}{\partial }_{i}=1$ and ${\Upsilon}_{j}>0,\sum_{j=1}^{m}{\Upsilon}_{j}=1$. Also, ${\Delta }_{p\left({a}_{{t^{\prime}}\left(i\right)z\left(j\right)}\right)}^{\prime}$ be the biggest point of IVPFS arguments ${\Delta }_{p\left({a}_{{t^{\prime}}\left(i\right)z\left(j\right)}\right)}^{\prime}=m{\Upsilon}_{j}\left(n{\partial }_{i}\left({a}_{ij}\right)\right)$. Where $m$ and $n$ be the balancing coefficients. If the ${\partial }_{i}={\left\{{\partial }_{1}, {\partial }_{2}, \dots \dots ,{\partial }_{n}\right\}}^{T}\to {\left\{\frac{1}{n}, \frac{1}{n}, \dots \dots ,\frac{1}{n}\right\}}^{T}$

and $${\Upsilon}_{j}={\left\{{\Upsilon}_{1}, {\Upsilon}_{2}, \dots \dots ,{\Upsilon}_{j}\right\}}^{T}\to {\left\{\frac{1}{m}, \frac{1}{m}, \dots \dots ,\frac{1}{m}\right\}}^{T}$$, 

then $${\left(m{\Upsilon}_{1}\left(n{\partial }_{1}\left({a}_{11}\right)\right), m{\Upsilon}_{1}\left(n{\partial }_{2}\left({a}_{21}\right)\right), \dots \dots ,m{\Upsilon}_{m}\left(n{\partial }_{n}\left({a}_{nm}\right)\right) \right)}^{T}\to {\left({\Delta }_{{a}_{11}},{\Delta }_{{a}_{12}},\dots \dots {\Delta }_{{a}_{nm}}\right)}^{T}$$.

#### Theorem 4.6.

Let ${\Delta }_{{a}_{ij}}^{\prime} =\left(\left[{{\lambda }^{\prime}}_{{a}_{ij}}^{l},{{\lambda }^{\prime}}_{{a}_{ij}}^{\mathpzc{u}}\right],\left[{{\eta }^{\prime}}_{{a}_{ij}}^{l},{{\eta }^{\prime}}_{{a}_{ij}}^{\mathpzc{u}}\right]\right)$ be a family of IVPFSVs. Then, the accumulation consequence of the IVPFSEHWA operator is also an IVPFSV.

$$IVPFSEHWA\left({\Delta }_{{a}_{11}}^{\prime},{\Delta }_{{a}_{12}}^{{{\prime}}},\dots \dots ,{\Delta }_{{a}_{nm}}^{\prime}\right)$$

4 $$=\left(\begin{array}{c}\left[\begin{array}{c}\frac{\sqrt{\prod_{j=1}^{m}{\left(\prod_{i=1}^{n}{\left(1+{\left({\lambda }_{p\left({a}_{{t^{\prime}}\left(i\right)z\left(j\right)}\right)}^{{\prime}l}\right)}^{2}\right)}^{{\partial }_{i}}\right)}^{{\Upsilon}_{j}}-\prod_{j=1}^{m}{\left(\prod_{i=1}^{n}{\left(1-{\left({\lambda }_{p\left({a}_{{t^{\prime}}\left(i\right)z\left(j\right)}\right)}^{{\prime}l}\right)}^{2}\right)}^{{\partial }_{i}}\right)}^{{\Upsilon}_{j}}}}{\sqrt{\prod_{j=1}^{m}{\left(\prod_{i=1}^{n}{\left(1+{\left({\lambda }_{p\left({a}_{{t^{\prime}}\left(i\right)z\left(j\right)}\right)}^{{\prime}l}\right)}^{2}\right)}^{{\partial }_{i}}\right)}^{{\Upsilon}_{j}}+\prod_{j=1}^{m}{\left(\prod_{i=1}^{n}{\left(1-{\left({\lambda }_{p\left({a}_{{t^{\prime}}\left(i\right)z\left(j\right)}\right)}^{{\prime}l}\right)}^{2}\right)}^{{\partial }_{i}}\right)}^{{\Upsilon}_{j}}}},\\ \frac{\sqrt{\prod_{j=1}^{m}{\left(\prod_{i=1}^{n}{\left(1+{\left({\lambda }_{p\left({a}_{{t^{\prime}}\left(i\right)z\left(j\right)}\right)}^{\mathcal{^{\prime}}\mathpzc{u}}\right)}^{2}\right)}^{{\partial }_{i}}\right)}^{{\Upsilon}_{j}}-\prod_{j=1}^{m}{\left(\prod_{i=1}^{n}{\left(1-{\left({\lambda }_{p\left({a}_{{t^{\prime}}\left(i\right)z\left(j\right)}\right)}^{\mathcal{^{\prime}}\mathpzc{u}}\right)}^{2}\right)}^{{\partial }_{i}}\right)}^{{\Upsilon}_{j}}}}{\sqrt{\prod_{j=1}^{m}{\left(\prod_{i=1}^{n}{\left(1+{\left({\lambda }_{p\left({a}_{{t^{\prime}}\left(i\right)z\left(j\right)}\right)}^{\mathcal{^{\prime}}\mathpzc{u}}\right)}^{2}\right)}^{{\partial }_{i}}\right)}^{{\Upsilon}_{j}}+\prod_{j=1}^{m}{\left(\prod_{i=1}^{n}{\left(1-{\left({\lambda }_{p\left({a}_{{t^{\prime}}\left(i\right)z\left(j\right)}\right)}^{\mathcal{^{\prime}}\mathpzc{u}}\right)}^{2}\right)}^{{\partial }_{i}}\right)}^{{\Upsilon}_{j}}}}\end{array}\right]\\ , \left[\begin{array}{c}\frac{\sqrt{2\prod_{j=1}^{m}{\left(\prod_{i=1}^{n}{\left({\left({\eta }_{p\left({a}_{{t^{\prime}}\left(i\right)z\left(j\right)}\right)}^{{\prime}l}\right)}^{2}\right)}^{{\partial }_{i}}\right)}^{{\Upsilon}_{j}}}}{\sqrt{\prod_{j=1}^{m}{\left(\prod_{i=1}^{n}{\left(2-{\left({\eta }_{p\left({a}_{{t^{\prime}}\left(i\right)z\left(j\right)}\right)}^{{\prime}l}\right)}^{2}\right)}^{{\partial }_{i}}\right)}^{{\Upsilon}_{j}}+\prod_{j=1}^{m}{\left(\prod_{i=1}^{n}{\left({\left({\eta }_{p\left({a}_{{t^{\prime}}\left(i\right)z\left(j\right)}\right)}^{{\prime}l}\right)}^{2}\right)}^{{\partial }_{i}}\right)}^{{\Upsilon}_{j}}}},\\ \frac{\sqrt{2\prod_{j=1}^{m}{\left(\prod_{i=1}^{n}{\left({\left({\eta }_{p\left({a}_{{t^{\prime}}\left(i\right)z\left(j\right)}\right)}^{\mathcal{^{\prime}}\mathpzc{u}}\right)}^{2}\right)}^{{\partial }_{i}}\right)}^{{\Upsilon}_{j}}}}{\sqrt{\prod_{j=1}^{m}{\left(\prod_{i=1}^{n}{\left(2-{\left({\eta }_{p\left({a}_{{t^{\prime}}\left(i\right)z\left(j\right)}\right)}^{\mathcal{^{\prime}}\mathpzc{u}}\right)}^{2}\right)}^{{\partial }_{i}}\right)}^{{\Upsilon}_{j}}+\prod_{j=1}^{m}{\left(\prod_{i=1}^{n}{\left({\left({\eta }_{p\left({a}_{{t^{\prime}}\left(i\right)z\left(j\right)}\right)}^{\mathcal{^{\prime}}\mathpzc{u}}\right)}^{2}\right)}^{{\partial }_{i}}\right)}^{{\Upsilon}_{j}}}}\end{array}\right]\end{array}\right)$$

where ${\partial }_{i}={\left\{{\partial }_{1}, {\partial }_{2}, \dots \dots ,{\partial }_{n}\right\}}^{T}$ are weights for professionals and ${\Upsilon}_{j}={\left\{{\Upsilon}_{1}, {\Upsilon}_{2}, \dots \dots ,{\Upsilon}_{j}\right\}}^{T}$ are the criteria weights with ${\partial }_{i}>0, \sum_{i=1}^{n}{\partial }_{i}=1$ and ${\Upsilon}_{j}>0, \sum_{j=1}^{m}{\Upsilon}_{j}=1$ and ${\Delta }_{p\left({a}_{{t^{\prime}}\left(i\right)z\left(j\right)}\right)}^{\prime}$ be the largest IVPFS arguments ${\Delta }_{p\left({a}_{{t^{\prime}}\left(i\right)z\left(j\right)}\right)}^{\prime}=m{\Upsilon}_{j}\left(n{\partial }_{i}\left({a}_{ij}\right)\right)$, and $m$, $n$ are the balancing coefficients. If the ${\partial }_{i}={\left\{{\partial }_{1}, {\partial }_{2}, \dots \dots ,{\partial }_{n}\right\}}^{T}\to {\left\{\frac{1}{n}, \frac{1}{n}, \dots \dots ,\frac{1}{n}\right\}}^{T}$

and $${\Upsilon}_{j}={\left\{{\Upsilon}_{1}, {\Upsilon}_{2}, \dots \dots ,{\Upsilon}_{j}\right\}}^{T}\to {\left\{\frac{1}{m}, \frac{1}{m}, \dots \dots ,\frac{1}{m}\right\}}^{T}$$, 

then $${\left(m{\Upsilon}_{1}\left(n{\partial }_{1}\left({a}_{11}\right)\right), m{\Upsilon}_{1}\left(n{\partial }_{2}\left({a}_{21}\right)\right), \dots \dots ,m{\Upsilon}_{m}\left(n{\partial }_{n}\left({a}_{nm}\right)\right) \right)}^{T}\to {\left({\Delta }_{{a}_{11}},{\Delta }_{{a}_{12}},\dots \dots {\Delta }_{{a}_{nm}}\right)}^{T}$$.

#### Proof:

Similar to Theorem 4.3.

In addition to the IVPFSEOWA operator, the IVPFSEHWA operator validates notable aspects. Some of these features are shift-invariance, idempotency, homogeneity, and boundedness. In what follows, we’ll go into further detail on these characteristics:

#### Proposition 4.7

If ${\Delta }_{{a}_{ij}}^{\prime} =\left(\left[{{\lambda }^{\prime}}_{{a}_{ij}}^{l},{{\lambda }^{\prime}}_{{a}_{ij}}^{\mathpzc{u}}\right],\left[{{\eta }^{\prime}}_{{a}_{ij}}^{l},{{\eta }^{\prime}}_{{a}_{ij}}^{\mathpzc{u}}\right]\right)$ be a collection of IVPFSVs. Also, ${\partial }_{i}$ and ${\Upsilon}_{j}$ correspondingly represent the weighting factors for professionals and criteria with ${\partial }_{i}>0 \mathrm{and} \sum_{i=1}^{n}{\partial }_{i}=1,$ ${\Upsilon}_{j}>0 \mathrm{and}\sum_{j=1}^{m}{\Upsilon}_{j}=1$.

### Idempotency

Let $${\Delta }_{p\left({a}_{{t^{\prime}}\left(i\right)z\left(j\right)}\right)}^{\prime}={\Delta }^{\prime}=\left(\left[{\lambda }^{{\prime}l},{\lambda }^{\mathcal{^{\prime}}\mathpzc{u}}\right],\left[{\eta }^{{\prime}l},{\eta }^{\mathcal{^{\prime}}\mathpzc{u}}\right]\right)$$, then

$$IVPFSEHWA\left({\Delta }_{{a}_{11}}^{\prime},{\Delta }_{{a}_{12}}^{{{\prime}}},\dots \dots ,{\Delta }_{{a}_{nm}}^{\prime}\right)={\Delta }^{\prime}$$

### Boundedness

Let $${\Delta }_{{a}_{ij}}^{-{\prime}}=\left(\left[min\left({\lambda }_{{a}_{ij} }^{{\prime}l}\right), min\left({\lambda }_{{a}_{ij}}^{\mathcal{^{\prime}}\mathpzc{u}}\right)\right], \left[max\left({\eta }_{{a}_{ij}}^{{\prime}l}\right), max\left({\eta }_{{a}_{ij}}^{\mathcal{^{\prime}}\mathpzc{u}}\right)\right]\right)$$

and $${\Delta }_{{a}_{ij}}^{+{\prime}}=\left(\left[max\left({\lambda }_{{a}_{ij} }^{{\prime}l}\right), max\left({\lambda }_{{a}_{ij}}^{\mathcal{^{\prime}}\mathpzc{u}}\right)\right], \left[min\left({\eta }_{{a}_{ij}}^{{\prime}l}\right), min\left({\eta }_{{a}_{ij}}^{\mathcal{^{\prime}}\mathpzc{u}}\right)\right]\right)$$. Then,

$${\Delta }_{{a}_{ij}}^{-{\prime}}\le IVPFSEHWA\left({\Delta }_{{a}_{11}}^{\prime},{\Delta }_{{a}_{12}}^{{{\prime}}},\dots \dots ,{\Delta }_{{a}_{nm}}^{\prime}\right)\le {\Delta }_{{a}_{ij}}^{+{\prime}}$$

### Monotonicity

Let ${\Delta }_{{a}_{ij}}^{\prime} =\left(\left[{{\lambda }^{\prime}}_{{a}_{ij}}^{l},{{\lambda }^{\prime}}_{{a}_{ij}}^{\mathpzc{u}}\right],\left[{{\eta }^{\prime}}_{{a}_{ij}}^{l},{{\eta }^{\prime}}_{{a}_{ij}}^{\mathpzc{u}}\right]\right) and \,\,{\Delta }_{{a}_{ij}}^{{\prime}*} =\left(\left[{{\lambda }^{\prime}}_{{a}_{ij}}^{l*},{{\lambda }^{\prime}}_{{a}_{ij}}^{\mathpzc{u}\mathcal{*}}\right],\left[{{\eta }^{\prime}}_{{a}_{ij}}^{l*},{{\eta }^{\prime}}_{{a}_{ij}}^{\mathpzc{u}\mathcal{*}}\right]\right)$ be the families of IVPFSVs. Then,

$$IVPFSEHWA\left({\Delta }_{{a}_{11}}^{\prime},{\Delta }_{{a}_{12}}^{{{\prime}}},\dots \dots ,{\Delta }_{{a}_{nm}}^{\prime}\right)\le IVPFSEHWA \left({\Delta }_{{a}_{11}}^{{\prime}*},{\Delta }_{{a}_{12}}^{{{\prime}}*},\dots \dots ,{\Delta }_{{a}_{nm}}^{{\prime}*}\right)$$

if $${\Delta }_{{a}_{nm}}^{\prime}\le {\Delta }_{{a}_{nm}}^{{\prime}*}$$ $$\forall i, j$$.

### Homogeneity

Let ${\Delta }_{{a}_{ij}}^{\prime} =\left(\left[{{\lambda }^{\prime}}_{{a}_{ij}}^{l},{{\lambda }^{\prime}}_{{a}_{ij}}^{\mathpzc{u}}\right],\left[{{\eta }^{\prime}}_{{a}_{ij}}^{l},{{\eta }^{\prime}}_{{a}_{ij}}^{\mathpzc{u}}\right]\right)$ be a family of IVPFSVs. Then,

$$IVPFSEHWA\left(\theta {\Delta }_{{a}_{11}}^{\prime},\theta {\Delta }_{{a}_{12}}^{{{\prime}}},\dots \dots ,\theta {\Delta }_{{a}_{nm}}^{\prime}\right)=\theta IVPFSEHWA\left({\Delta }_{{a}_{11}}^{\prime},{\Delta }_{{a}_{12}}^{{^{\prime}}},\dots \dots ,{\Delta }_{{a}_{nm}}^{\prime}\right)$$ for $$\theta >0.$$

### Shift Invariance

Let ${\Delta }_{{a}_{ij}}^{\prime} =\left(\left[{{\lambda }^{\prime}}_{{a}_{ij}}^{l},{{\lambda }^{\prime}}_{{a}_{ij}}^{\mathpzc{u}}\right],\left[{{\eta }^{\prime}}_{{a}_{ij}}^{l},{{\eta }^{\prime}}_{{a}_{ij}}^{\mathpzc{u}}\right]\right)$ be a family of IVPFSVs and ${\Delta }^{\prime}=\left(\left[{\lambda }^{{\prime}l},{\lambda }^{\mathcal{^{\prime}}\mathpzc{u}}\right],\left[{\eta }^{{\prime}l},{\eta }^{\mathcal{^{\prime}}\mathpzc{u}}\right]\right)$ be an IVPFSV. Then,

$$IVPFSEHWA \left({\Delta }_{{a}_{11}}^{\prime}{\oplus }_{\varepsilon }{\Delta }^{\prime}, {\Delta }_{{a}_{12}}^{\prime}{\oplus }_{\varepsilon }{\Delta }^{\prime},\dots ,{\Delta }_{{a}_{nm}}^{\prime}{\oplus }_{\varepsilon }{\Delta }^{\prime}\right)$$

$$= IVPFSEHWA\left({\Delta }_{{a}_{11}}^{\prime},{\Delta }_{{a}_{12}}^{{^{\prime}}},\dots \dots ,{\Delta }_{{a}_{nm}}^{\prime}\right){\oplus }_{\varepsilon }{\Delta }^{\prime}$$

## Interval-valued Pythagorean fuzzy soft Einstein weighted geometric aggregation operators

The following section describes the IVPFSEOWG and IVPFSEHWG operators, concentrating on their basic characteristics.

### Definition 5.1.

Let ${\Delta }_{{a}_{ij}}=\left(\left[{\lambda }_{{a}_{ij}}^{l},{\lambda }_{{a}_{ij}}^{\mathpzc{u}}\right],\left[{\eta }_{{a}_{ij}}^{l},{\eta }_{{a}_{ij}}^{\mathpzc{u}}\right]\right)$ be a family of IVPFSVs. Then IVPFSEOWG is defined as:

$$IVPFSEOWG\left({\Delta }_{{a}_{11}},{\Delta }_{{a}_{12}},\dots \dots ,{\Delta }_{{a}_{nm}}\right)$$

$$={\Upsilon}_{1}{{\partial }_{1}\Delta }_{p\left({a}_{{t^{\prime}}\left(1\right)z\left(1\right)}\right)}{{\otimes}}_{\varepsilon }{\Upsilon}_{1}{{\partial }_{2}\Delta }_{p\left({a}_{{t^{\prime}}\left(2\right)z\left(1\right)}\right)}{{\otimes}}_{\varepsilon }\dots \dots {{\otimes}}_{\varepsilon }{\Upsilon}_{m}{\partial }_{n}{\Delta }_{p\left({a}_{{t^{\prime}}\left(n\right)z\left(m\right)}\right)}$$

$$={\left(\stackrel{m}{\underset{j=1}{{{\otimes}}_{\varepsilon }}}{\left(\stackrel{n}{\underset{i=1}{{{\otimes}}_{\varepsilon }}}{\Delta }_{p\left({a}_{{t^{\prime}}\left(i\right)z\left(j\right)}\right)}\right)}^{{\partial }_{i}}\right)}^{{\Upsilon}_{j}}$$

where ${\partial }_{i}={\left\{{\partial }_{1}, {\partial }_{2}, \dots \dots ,{\partial }_{n}\right\}}^{T}$ and ${\Upsilon}_{j}={\left\{{\Upsilon}_{1}, {\Upsilon}_{2}, \dots \dots ,{\Upsilon}_{j}\right\}}^{T}$ be the weights for professionals and criteria such as ${\partial }_{i}>0,\sum_{i=1}^{n}{\partial }_{i}=1$ and ${\Upsilon}_{j}>0,\sum_{j=1}^{m}{\Upsilon}_{j}=1$. Also, ${\Delta }_{\sigma \left({a}_{\mathfrak{r}\left(i\right)z\left(j\right)}\right)}$ be the leading component of ${i}^{th}$ row and ${j}^{th}$ column in $\left({\Delta }_{{a}_{11}},{\Delta }_{{a}_{12}},\dots \dots ,{\Delta }_{{a}_{nm}}\right)$, such as ${\Delta }_{p\left({a}_{{t^{\prime}}\left(i\right)z\left(j\right)}\right)}\le {\Delta }_{p\left({a}_{{t^{\prime}}\left(i-1\right)z\left(j\right)}\right)}$ and ${\Delta }_{p\left({a}_{{t^{\prime}}\left(i\right)z\left(j\right)}\right)}\le {\Delta }_{p\left({a}_{{t^{\prime}}\left(i\right)z\left(j-1\right)}\right)}$ $\forall i,j$.

### Theorem 5.2.

Let ${\Delta }_{{a}_{ij}}=\left(\left[{\lambda }_{{a}_{ij}}^{l},{\lambda }_{{a}_{ij}}^{\mathpzc{u}}\right],\left[{\eta }_{{a}_{ij}}^{l},{\eta }_{{a}_{ij}}^{\mathpzc{u}}\right]\right)$ be a collection of IVPFSVs. Then, the aggregated outcome of the IVPFSEOWG operator is also an IVPFSV.

$$IVPFSEOWG\left({\Delta }_{{a}_{11}},{\Delta }_{{a}_{12}},\dots \dots {\Delta }_{{a}_{nm}}\right)$$

5 $$=\left(\begin{array}{c}\left[\begin{array}{c}\frac{\sqrt{2\prod_{j=1}^{m}{\left(\prod_{i=1}^{n}{\left({\left({\lambda }_{p\left({a}_{{t^{\prime}}\left(i\right)z\left(j\right)}\right)}^{l}\right)}^{2}\right)}^{{\partial }_{i}}\right)}^{{\Upsilon}_{j}}}}{\sqrt{\prod_{j=1}^{m}{\left(\prod_{i=1}^{n}{\left(2-{\left({\lambda }_{p\left({a}_{{t^{\prime}}\left(i\right)z\left(j\right)}\right)}^{l}\right)}^{2}\right)}^{{\partial }_{i}}\right)}^{{\Upsilon}_{j}}+\prod_{j=1}^{m}{\left(\prod_{i=1}^{n}{\left({\left({\lambda }_{p\left({a}_{{t^{\prime}}\left(i\right)z\left(j\right)}\right)}^{l}\right)}^{2}\right)}^{{\partial }_{i}}\right)}^{{\Upsilon}_{j}}}},\\ \frac{\sqrt{2\prod_{j=1}^{m}{\left(\prod_{i=1}^{n}{\left({\left({\lambda }_{p\left({a}_{{t^{\prime}}\left(i\right)z\left(j\right)}\right)}^{\mathpzc{u}}\right)}^{2}\right)}^{{\partial }_{i}}\right)}^{{\Upsilon}_{j}}}}{\sqrt{\prod_{j=1}^{m}{\left(\prod_{i=1}^{n}{\left(2-{\left({\lambda }_{p\left({a}_{{t^{\prime}}\left(i\right)z\left(j\right)}\right)}^{\mathpzc{u}}\right)}^{2}\right)}^{{\partial }_{i}}\right)}^{{\Upsilon}_{j}}+\prod_{j=1}^{m}{\left(\prod_{i=1}^{n}{\left({\left({\lambda }_{p\left({a}_{{t^{\prime}}\left(i\right)z\left(j\right)}\right)}^{\mathpzc{u}}\right)}^{2}\right)}^{{\partial }_{i}}\right)}^{{\Upsilon}_{j}}}}\end{array}\right],\\ , \left[\begin{array}{c}\frac{\sqrt{\prod_{j=1}^{m}{\left(\prod_{i=1}^{n}{\left(1+{\left({\eta }_{p\left({a}_{{t^{\prime}}\left(i\right)z\left(j\right)}\right)}^{l}\right)}^{2}\right)}^{{\partial }_{i}}\right)}^{{\Upsilon}_{j}}-\prod_{j=1}^{m}{\left(\prod_{i=1}^{n}{\left(1-{\left({\eta }_{p\left({a}_{{t^{\prime}}\left(i\right)z\left(j\right)}\right)}^{l}\right)}^{2}\right)}^{{\partial }_{i}}\right)}^{{\Upsilon}_{j}}}}{\sqrt{\prod_{j=1}^{m}{\left(\prod_{i=1}^{n}{\left(1+{\left({\eta }_{p\left({a}_{{t^{\prime}}\left(i\right)z\left(j\right)}\right)}^{l}\right)}^{2}\right)}^{{\partial }_{i}}\right)}^{{\Upsilon}_{j}}+\prod_{j=1}^{m}{\left(\prod_{i=1}^{n}{\left(1-{\left({\eta }_{p\left({a}_{{t^{\prime}}\left(i\right)z\left(j\right)}\right)}^{l}\right)}^{2}\right)}^{{\partial }_{i}}\right)}^{{\Upsilon}_{j}}}},\\ \frac{\sqrt{\prod_{j=1}^{m}{\left(\prod_{i=1}^{n}{\left(1+{\left({\eta }_{p\left({a}_{{t^{\prime}}\left(i\right)z\left(j\right)}\right)}^{\mathpzc{u}}\right)}^{2}\right)}^{{\partial }_{i}}\right)}^{{\Upsilon}_{j}}-\prod_{j=1}^{m}{\left(\prod_{i=1}^{n}{\left(1-{\left({\eta }_{p\left({a}_{{t^{\prime}}\left(i\right)z\left(j\right)}\right)}^{\mathpzc{u}}\right)}^{2}\right)}^{{\partial }_{i}}\right)}^{{\Upsilon}_{j}}}}{\sqrt{\prod_{j=1}^{m}{\left(\prod_{i=1}^{n}{\left(1+{\left({\eta }_{p\left({a}_{{t^{\prime}}\left(i\right)z\left(j\right)}\right)}^{\mathpzc{u}}\right)}^{2}\right)}^{{\partial }_{i}}\right)}^{{\Upsilon}_{j}}+\prod_{j=1}^{m}{\left(\prod_{i=1}^{n}{\left(1-{\left({\eta }_{p\left({a}_{{t^{\prime}}\left(i\right)z\left(j\right)}\right)}^{\mathpzc{u}}\right)}^{2}\right)}^{{\partial }_{i}}\right)}^{{\Upsilon}_{j}}}}\end{array}\right]\end{array}\right)$$

where ${\partial }_{i}={\left\{{\partial }_{1}, {\partial }_{2}, \dots \dots ,{\partial }_{n}\right\}}^{T}$ are the weights of professionals and ${\Upsilon}_{j}={\left\{{\Upsilon}_{1}, {\Upsilon}_{2}, \dots \dots ,{\Upsilon}_{j}\right\}}^{T}$ are the weights of the criteria with ${\partial }_{i}>0 \mathrm{and}\sum_{i=1}^{n}{\partial }_{i}=1$, and ${\Upsilon}_{j}>0 \mathrm{and} \sum_{j=1}^{m}{\Upsilon}_{j}=1$. Also, ${\Delta }_{p\left({a}_{{t^{\prime}}\left(i\right)z\left(j\right)}\right)}$ be the leading component of ${i}^{th}$ row and ${j}^{th}$ column in $\left({\Delta }_{{a}_{11}},{\Delta }_{{a}_{12}},\dots \dots ,{\Delta }_{{a}_{nm}}\right)$, such as ${\Delta }_{p\left({a}_{{t^{\prime}}\left(i\right)z\left(j\right)}\right)}\le {\Delta }_{p\left({a}_{{t^{\prime}}\left(i-1\right)z\left(j\right)}\right)}$ and ${\Delta }_{p\left({a}_{{t^{\prime}}\left(i\right)z\left(j\right)}\right)}\le {\Delta }_{p\left({a}_{{t^{\prime}}\left(i\right)z\left(j-1\right)}\right)}$ $\forall i,j$.

### Proof:

Similar to Theorem 4.3.

### Proposition 5.3.

If $\Delta { }_{{a}_{ij}}=\left(\left[{\lambda }_{{a}_{ij}}^{l},{\lambda }_{{a}_{ij}}^{\mho }\right],\left[{\eta }_{{a}_{ij}}^{l},{\eta }_{{a}_{ij}}^{\mho }\right]\right)$ consists of IVPFSVs. Also, ${\partial }_{i}$ and ${\Upsilon}_{j}$ correspondingly represent the weighting factors for professionals and criteria with ${\partial }_{i}>0 \mathrm{and} \sum_{i=1}^{n}{\partial }_{i}=1,$ ${\Upsilon}_{j}>0 \mathrm{and}\sum_{j=1}^{m}{\Upsilon}_{j}=1$.

### Idempotency

Let $\Delta { }_{{a}_{ij}}=\Delta { }_{{a}_{o}}=\left(\left[{\lambda }_{{a}_{o}}^{l},{\lambda }_{{a}_{o}}^{\mathpzc{u}}\right],\left[{\eta }_{{a}_{o}}^{l},{\eta }_{{a}_{o}}^{\mathpzc{u}}\right]\right)$ holds. Then,

$$IVPFSEOWG\left({\Delta }_{{a}_{11}},{\Delta }_{{a}_{12}},\dots \dots {\Delta }_{{a}_{nm}}\right)=\Delta { }_{{a}_{o}}$$

### Boundedness

Let $${\Delta }_{{a}_{ij}}^{-}=\left(\left[min\left({\lambda }_{{a}_{ij} }^{l}\right), min\left({\lambda }_{{a}_{ij}}^{\mathpzc{u}}\right)\right], \left[max\left({\eta }_{{a}_{ij}}^{l}\right), max\left({\eta }_{{a}_{ij}}^{\mathpzc{u}}\right)\right]\right)$$

and $${\Delta }_{{a}_{ij}}^{+}=\left(\left[max\left({\lambda }_{{a}_{ij} }^{l}\right), max\left({\lambda }_{{a}_{ij}}^{\mathpzc{u}}\right)\right], \left[min\left({\eta }_{{a}_{ij}}^{l}\right), min\left({\eta }_{{a}_{ij}}^{\mathpzc{u}}\right)\right]\right)$$. Then,

$${\Delta }_{{a}_{ij}}^{-}\le IVPFSEOWG\left({\Delta }_{{a}_{11}},{\Delta }_{{a}_{12}},\dots \dots {\Delta }_{{a}_{nm}}\right)\le {\Delta }_{{a}_{ij}}^{+}$$

### Monotonicity

Let $\Delta { }_{{a}_{ij}}=\left(\left[{\lambda }_{{a}_{ij} }^{l},{\lambda }_{{a}_{ij}}^{\mathpzc{u}}\right],\left[{\eta }_{{a}_{ij}}^{l},{\eta }_{{a}_{ij}}^{\mathpzc{u}}\right]\right) and {\Delta }_{{a}_{ij}}^{*} =\left(\left[{{\lambda }^{*}}_{{a}_{ij} }^{l},{{\lambda }^{*}}_{{a}_{ij}}^{\mathpzc{u}}\right],\left[{{\eta }^{*}}_{{a}_{ij}}^{l},{{\eta }^{*}}_{{a}_{ij}}^{\mathpzc{u}}\right]\right)$ be the families of IVPFSVs. Then,

$$IVPFSEOWG\left({\Delta }_{{a}_{11}},{\Delta }_{{a}_{12}},\dots \dots {\Delta }_{{a}_{nm}}\right)\le IVPFSEOWG \left({\Delta }_{{a}_{11}}^{*},{\Delta }_{{a}_{12}}^{*},\dots \dots \dots , {\Delta }_{{a}_{nm}}^{*}\right)$$

if $${\Delta }_{{a}_{nm}}\le {\Delta }_{{a}_{nm}}^{*}$$ $$\forall i, j$$.

### Homogeneity

Let $\Delta { }_{{a}_{ij}}=\left(\left[{\lambda }_{{a}_{ij} }^{l},{\lambda }_{{a}_{ij}}^{\mathpzc{u}}\right],\left[{\eta }_{{a}_{ij}}^{l},{\eta }_{{a}_{ij}}^{\mathpzc{u}}\right]\right)$ be a family of IVPFSVs. Then,

$$IVPFSEOWG\left(\theta {\Delta }_{{a}_{11}},\theta {\Delta }_{{a}_{12}},\dots \dots \theta {\Delta }_{{a}_{nm}}\right)$$

$$=\theta IVPFSEOWG\left({\Delta }_{{a}_{11}},{\Delta }_{{a}_{12}},\dots \dots {\Delta }_{{a}_{nm}}\right)$$

for $$\theta >0$$*.*

### Shift Invariance

Let $\Delta { }_{{a}_{ij}}=\left(\left[{\lambda }_{{a}_{ij} }^{l},{\lambda }_{{a}_{ij}}^{\mathpzc{u}}\right],\left[{\eta }_{{a}_{ij}}^{l},{\eta }_{{a}_{ij}}^{\mathpzc{u}}\right]\right)$ be a collection of IVPFSVs and $\Delta { }_{a}=\left(\left[{\lambda }_{a }^{l},{\lambda }_{a}^{\mathpzc{u}}\right],\left[{\eta }_{a}^{l},{\eta }_{a}^{\mathpzc{u}}\right]\right)$ be an IVPFSV. Then,

$$IVPFSEOWG \left(\Delta { }_{{a}_{11}}{{\otimes}}_{\varepsilon }\Delta { }_{a},\Delta { }_{{a}_{12}}{{\otimes}}_{\varepsilon }\Delta { }_{a},\dots ,\Delta { }_{{a}_{nm}}{{\otimes}}_{\varepsilon }\Delta { }_{a}\right)$$

$$= IVPFSEOWG\left({\Delta }_{{a}_{11}},{\Delta }_{{a}_{12}},\dots \dots {\Delta }_{{a}_{nm}}\right){{\otimes}}_{\varepsilon }\Delta { }_{a}$$

#### Definition 5.4

Let ${\Delta }_{{a}_{ij}}^{\prime} =\left(\left[{{\lambda }^{\prime}}_{{a}_{ij}}^{l},{{\lambda }^{\prime}}_{{a}_{ij}}^{\mathpzc{u}}\right],\left[{{\eta }^{\prime}}_{{a}_{ij}}^{l},{{\eta }^{\prime}}_{{a}_{ij}}^{\mathpzc{u}}\right]\right)$ be a family of IVPFSVs. Then, the IVPFSEHWG operator is formulated as:

$$IVPFSEHWG\left({\Delta }_{{a}_{11}}^{\prime},{\Delta }_{{a}_{12}}^{{{\prime}}},\dots \dots ,{\Delta }_{{a}_{nm}}^{\prime}\right)$$

$$=\left({\left(\stackrel{m}{\underset{j=1}{{{\otimes}}_{\varepsilon }}}{\left(\stackrel{n}{\underset{i=1}{{{\otimes}}_{\varepsilon }}}{\Delta }_{p\left({a}_{{t^{\prime}}\left(i\right)z\left(j\right)}\right)}^{\prime}\right)}^{{\partial }_{i}}\right)}^{{\Upsilon}_{j}}\right)$$

where ${\partial }_{i}={\left\{{\partial }_{1}, {\partial }_{2}, \dots \dots ,{\partial }_{n}\right\}}^{T}$ and ${\Upsilon}_{j}={\left\{{\Upsilon}_{1}, {\Upsilon}_{2}, \dots \dots ,{\Upsilon}_{j}\right\}}^{T}$ are the vectors of weights given to the experts and the criteria, respectively, fulfilling ${\partial }_{i}>0,\sum_{i=1}^{n}{\partial }_{i}=1$ and ${\Upsilon}_{j}>0,\sum_{j=1}^{m}{\Upsilon}_{j}=1$. Moreover, ${\Delta }_{p\left({a}_{{t^{\prime}}\left(i\right)z\left(j\right)}\right)}^{\prime}$ be the largest element of IVPFS arguments ${\Delta }_{p\left({a}_{{t^{\prime}}\left(i\right)z\left(j\right)}\right)}^{\prime}=m{\Upsilon}_{j}\left(n{\partial }_{i}\left({a}_{ij}\right)\right)$.

Where $m$ and $n$ be the balancing coefficients. If the ${\partial }_{i}={\left\{{\partial }_{1}, {\partial }_{2}, \dots \dots ,{\partial }_{n}\right\}}^{T}\to {\left\{\frac{1}{n}, \frac{1}{n}, \dots \dots ,\frac{1}{n}\right\}}^{T}$

and $${\Upsilon}_{j}={\left\{{\Upsilon}_{1}, {\Upsilon}_{2}, \dots \dots ,{\Upsilon}_{j}\right\}}^{T}\to {\left\{\frac{1}{m}, \frac{1}{m}, \dots \dots ,\frac{1}{m}\right\}}^{T}$$, then

$${\left(m{\Upsilon}_{1}\left(n{\partial }_{1}\left({a}_{11}\right)\right), m{\Upsilon}_{1}\left(n{\partial }_{2}\left({a}_{21}\right)\right), \dots \dots ,m{\Upsilon}_{m}\left(n{\partial }_{n}\left({a}_{nm}\right)\right) \right)}^{T}\to {\left({\Delta }_{{a}_{11}},{\Delta }_{{a}_{12}},\dots \dots {\Delta }_{{a}_{nm}}\right)}^{T}.$$

#### Theorem 5.5

Let ${\Delta }_{{a}_{ij}}^{\prime} =\left(\left[{{\lambda }^{\prime}}_{{a}_{ij}}^{l},{{\lambda }^{\prime}}_{{a}_{ij}}^{\mathpzc{u}}\right],\left[{{\eta }^{\prime}}_{{a}_{ij}}^{l},{{\eta }^{\prime}}_{{a}_{ij}}^{\mathpzc{u}}\right]\right)$ be a collection of IVPFSVs. Then, the aggregated outcome of the IVPFSEHWG operator is also an IVPFSV.

$$IVPFSEHWG\left({\Delta }_{{a}_{11}}^{\prime},{\Delta }_{{a}_{12}}^{{{\prime}}},\dots \dots ,{\Delta }_{{a}_{nm}}^{\prime}\right)$$

6 $$=\left(\begin{array}{c}\begin{array}{c}\left[\begin{array}{c}\frac{\sqrt{2\prod_{j=1}^{m}{\left(\prod_{i=1}^{n}{\left({\left({\lambda }_{p\left({a}_{{t^{\prime}}\left(i\right)z\left(j\right)}\right)}^{{\prime}l}\right)}^{2}\right)}^{{\partial }_{i}}\right)}^{{\Upsilon}_{j}}}}{\sqrt{\prod_{j=1}^{m}{\left(\prod_{i=1}^{n}{\left(2-{\left({\lambda }_{p\left({a}_{{t^{\prime}}\left(i\right)z\left(j\right)}\right)}^{{\prime}l}\right)}^{2}\right)}^{{\partial }_{i}}\right)}^{{\Upsilon}_{j}}+\prod_{j=1}^{m}{\left(\prod_{i=1}^{n}{\left({\left({\lambda }_{p\left({a}_{{t^{\prime}}\left(i\right)z\left(j\right)}\right)}^{{\prime}l}\right)}^{2}\right)}^{{\partial }_{i}}\right)}^{{\Upsilon}_{j}}}},\\ \frac{\sqrt{2\prod_{j=1}^{m}{\left(\prod_{i=1}^{n}{\left({\left({\lambda }_{p\left({a}_{{t^{\prime}}\left(i\right)z\left(j\right)}\right)}^{\mathcal{^{\prime}}\mathpzc{u}}\right)}^{2}\right)}^{{\partial }_{i}}\right)}^{{\Upsilon}_{j}}}}{\sqrt{\prod_{j=1}^{m}{\left(\prod_{i=1}^{n}{\left(2-{\left({\lambda }_{p\left({a}_{{t^{\prime}}\left(i\right)z\left(j\right)}\right)}^{\mathcal{^{\prime}}\mathpzc{u}}\right)}^{2}\right)}^{{\partial }_{i}}\right)}^{{\Upsilon}_{j}}+\prod_{j=1}^{m}{\left(\prod_{i=1}^{n}{\left({\left({\lambda }_{p\left({a}_{{t^{\prime}}\left(i\right)z\left(j\right)}\right)}^{\mathcal{^{\prime}}\mathpzc{u}}\right)}^{2}\right)}^{{\partial }_{i}}\right)}^{{\Upsilon}_{j}}}}\end{array}\right],\end{array}\\ \left[\begin{array}{c}\frac{\sqrt{\prod_{j=1}^{m}{\left(\prod_{i=1}^{n}{\left(1+{\left({\eta }_{p\left({a}_{{t^{\prime}}\left(i\right)z\left(j\right)}\right)}^{{\prime}l}\right)}^{2}\right)}^{{\partial }_{i}}\right)}^{{\Upsilon}_{j}}-\prod_{j=1}^{m}{\left(\prod_{i=1}^{n}{\left(1-{\left({\eta }_{p\left({a}_{{t^{\prime}}\left(i\right)z\left(j\right)}\right)}^{{\prime}l}\right)}^{2}\right)}^{{\partial }_{i}}\right)}^{{\Upsilon}_{j}}}}{\sqrt{\prod_{j=1}^{m}{\left(\prod_{i=1}^{n}{\left(1+{\left({\eta }_{p\left({a}_{{t^{\prime}}\left(i\right)z\left(j\right)}\right)}^{{\prime}l}\right)}^{2}\right)}^{{\partial }_{i}}\right)}^{{\Upsilon}_{j}}+\prod_{j=1}^{m}{\left(\prod_{i=1}^{n}{\left(1-{\left({\eta }_{p\left({a}_{{t^{\prime}}\left(i\right)z\left(j\right)}\right)}^{{\prime}l}\right)}^{2}\right)}^{{\partial }_{i}}\right)}^{{\Upsilon}_{j}}}},\\ \frac{\sqrt{\prod_{j=1}^{m}{\left(\prod_{i=1}^{n}{\left(1+{\left({\eta }_{p\left({a}_{{t^{\prime}}\left(i\right)z\left(j\right)}\right)}^{\mathcal{^{\prime}}\mathpzc{u}}\right)}^{2}\right)}^{{\partial }_{i}}\right)}^{{\Upsilon}_{j}}-\prod_{j=1}^{m}{\left(\prod_{i=1}^{n}{\left(1-{\left({\eta }_{p\left({a}_{{t^{\prime}}\left(i\right)z\left(j\right)}\right)}^{\mathcal{^{\prime}}\mathpzc{u}}\right)}^{2}\right)}^{{\partial }_{i}}\right)}^{{\Upsilon}_{j}}}}{\sqrt{\prod_{j=1}^{m}{\left(\prod_{i=1}^{n}{\left(1+{\left({\eta }_{p\left({a}_{{t^{\prime}}\left(i\right)z\left(j\right)}\right)}^{\mathcal{^{\prime}}\mathpzc{u}}\right)}^{2}\right)}^{{\partial }_{i}}\right)}^{{\Upsilon}_{j}}+\prod_{j=1}^{m}{\left(\prod_{i=1}^{n}{\left(1-{\left({\eta }_{p\left({a}_{{t^{\prime}}\left(i\right)z\left(j\right)}\right)}^{\mathcal{^{\prime}}\mathpzc{u}}\right)}^{2}\right)}^{{\partial }_{i}}\right)}^{{\Upsilon}_{j}}}}\end{array}\right]\end{array}\right)$$

where ${\partial }_{i}={\left\{{\partial }_{1}, {\partial }_{2}, \dots \dots ,{\partial }_{n}\right\}}^{T}$ and ${\partial }_{i}={\left\{{\Upsilon}_{1}, {\Upsilon}_{2}, \dots \dots ,{\Upsilon}_{j}\right\}}^{T}$ denote the weight vectors associated with the experts and the parameters, respectively, where ${\partial }_{i}>0,\sum_{i=1}^{n}{\partial }_{i}=1$ and ${\Upsilon}_{j}>0,\sum_{j=1}^{m}{\Upsilon}_{j}=1$ and ${\Delta }_{p\left({a}_{{t^{\prime}}\left(i\right)z\left(j\right)}\right)}^{\prime}$ be the largest element of IVPFS arguments ${\Delta }_{p\left({a}_{{t^{\prime}}\left(i\right)z\left(j\right)}\right)}^{\prime}=m{\Upsilon}_{j}\left(n{\partial }_{i}\left({a}_{ij}\right)\right)$, and $m$, $n$ are the balancing coefficients.

 If the $${\partial }_{i}={\left\{{\partial }_{1}, {\partial }_{2}, \dots \dots ,{\partial }_{n}\right\}}^{T}\to {\left\{\frac{1}{n}, \frac{1}{n}, \dots \dots ,\frac{1}{n}\right\}}^{T}$$

and $${\Upsilon}_{j}={\left\{{\Upsilon}_{1}, {\Upsilon}_{2}, \dots \dots ,{\Upsilon}_{j}\right\}}^{T}\to {\left\{\frac{1}{m}, \frac{1}{m}, \dots \dots ,\frac{1}{m}\right\}}^{T}$$,

then $${\left(m{\Upsilon}_{1}\left(n{\partial }_{1}\left({a}_{11}\right)\right), m{\Upsilon}_{1}\left(n{\partial }_{2}\left({a}_{21}\right)\right), \dots \dots ,m{\Upsilon}_{m}\left(n{\partial }_{n}\left({a}_{nm}\right)\right) \right)}^{T}\to {\left({\Delta }_{{a}_{11}},{\Delta }_{{a}_{12}},\dots \dots {\Delta }_{{a}_{nm}}\right)}^{T}$$.

#### Proof:

Similar to Theorem 4.3.

#### Proposition 5.6

If ${\Delta }_{{a}_{ij}}^{\prime} =\left(\left[{{\lambda }^{\prime}}_{{a}_{ij}}^{l},{{\lambda }^{\prime}}_{{a}_{ij}}^{\mathpzc{u}}\right],\left[{{\eta }^{\prime}}_{{a}_{ij}}^{l},{{\eta }^{\prime}}_{{a}_{ij}}^{\mathpzc{u}}\right]\right)$ consists of IVPFSVs. Also, ${\partial }_{i}$ and ${\Upsilon}_{j}$ correspondingly represent the weighting factors for professionals and criteria with ${\partial }_{i}>0 \mathrm{and} \sum_{i=1}^{n}{\partial }_{i}=1,$ ${\Upsilon}_{j}>0 \mathrm{and}\sum_{j=1}^{m}{\Upsilon}_{j}=1$.

### Idempotency

Let $${\Delta }_{p\left({a}_{{t^{\prime}}\left(i\right)z\left(j\right)}\right)}^{\prime}={\Delta }^{\prime}=\left(\left[{\lambda }^{{\prime}l},{\lambda }^{\mathcal{^{\prime}}\mathpzc{u}}\right],\left[{\eta }^{{\prime}l},{\eta }^{\mathcal{^{\prime}}\mathpzc{u}}\right]\right)$$ $$\forall$$ $$i,j$$. Then,

$$IVPFSEHWG\left({\Delta }_{{a}_{11}}^{\prime},{\Delta }_{{a}_{12}}^{{{\prime}}},\dots \dots ,{\Delta }_{{a}_{nm}}^{\prime}\right)={\Delta }^{\prime}$$

### Boundedness

Let $${\Delta }_{{a}_{ij}}^{-{\prime}}=\left(\left[min\left({\lambda }_{{a}_{ij} }^{{\prime}l}\right), min\left({\lambda }_{{a}_{ij}}^{\mathcal{^{\prime}}\mathpzc{u}}\right)\right], \left[max\left({\eta }_{{a}_{ij}}^{{\prime}l}\right), max\left({\eta }_{{a}_{ij}}^{\mathcal{^{\prime}}\mathpzc{u}}\right)\right]\right)$$

and $${\Delta }_{{a}_{ij}}^{+{\prime}}=\left(\left[max\left({\lambda }_{{a}_{ij} }^{{\prime}l}\right), max\left({\lambda }_{{a}_{ij}}^{\mathcal{^{\prime}}\mathpzc{u}}\right)\right], \left[min\left({\eta }_{{a}_{ij}}^{{\prime}l}\right), min\left({\eta }_{{a}_{ij}}^{\mathcal{^{\prime}}\mathpzc{u}}\right)\right]\right)$$. Then,

$${\Delta }_{{a}_{ij}}^{-{\prime}}\le IVPFSEHWG\left({\Delta }_{{a}_{11}}^{\prime},{\Delta }_{{a}_{12}}^{{{\prime}}},\dots \dots ,{\Delta }_{{a}_{nm}}^{\prime}\right)\le {\Delta }_{{a}_{ij}}^{+{\prime}}$$

### Monotonicity

Let ${\Delta }_{{a}_{ij}}^{\prime} =\left(\left[{{\lambda }^{\prime}}_{{a}_{ij}}^{l},{{\lambda }^{\prime}}_{{a}_{ij}}^{\mathpzc{u}}\right],\left[{{\eta }^{\prime}}_{{a}_{ij}}^{l},{{\eta }^{\prime}}_{{a}_{ij}}^{\mathpzc{u}}\right]\right) and {\Delta }_{{a}_{ij}}^{{\prime}*} =\left(\left[{{\lambda }^{\prime}}_{{a}_{ij}}^{l*},{{\lambda }^{\prime}}_{{a}_{ij}}^{\mathpzc{u}\mathcal{*}}\right],\left[{{\eta }^{\prime}}_{{a}_{ij}}^{l*},{{\eta }^{\prime}}_{{a}_{ij}}^{\mathpzc{u}\mathcal{*}}\right]\right)$ be the families of IVPFSVs. Then,

$$IVPFSEHWG\left({\Delta }_{{a}_{11}}^{\prime},{\Delta }_{{a}_{12}}^{{{\prime}}},\dots \dots ,{\Delta }_{{a}_{nm}}^{\prime}\right)\le IVPFSEHWG\left({\Delta }_{{a}_{11}}^{{\prime}*},{\Delta }_{{a}_{12}}^{{{\prime}}*},\dots \dots ,{\Delta }_{{a}_{nm}}^{{\prime}*}\right)$$

if $${\Delta }_{{a}_{nm}}^{\prime}\le {\Delta }_{{a}_{nm}}^{{\prime}*}$$ $$\forall i, j$$.

### Homogeneity

Let ${\Delta }_{{a}_{ij}}^{\prime} =\left(\left[{{\lambda }^{\prime}}_{{a}_{ij}}^{l},{{\lambda }^{\prime}}_{{a}_{ij}}^{\mathpzc{u}}\right],\left[{{\eta }^{\prime}}_{{a}_{ij}}^{l},{{\eta }^{\prime}}_{{a}_{ij}}^{\mathpzc{u}}\right]\right)$ be a family of IVPFSVs. Then,

$$IVPFSEHWG\left(\theta {\Delta }_{{a}_{11}}^{\prime},\theta {\Delta }_{{a}_{12}}^{{{\prime}}},\dots \dots ,\theta {\Delta }_{{a}_{nm}}^{\prime}\right)$$

$$=\theta IVPFSEHWG\left({\Delta }_{{a}_{11}}^{\prime},{\Delta }_{{a}_{12}}^{{{\prime}}},\dots \dots ,{\Delta }_{{a}_{nm}}^{\prime}\right)$$

for $$\theta >0.$$

### Shift Invariance

Let ${\Delta }_{{a}_{ij}}^{\prime} =\left(\left[{{\lambda }^{\prime}}_{{a}_{ij}}^{l},{{\lambda }^{\prime}}_{{a}_{ij}}^{\mathpzc{u}}\right],\left[{{\eta }^{\prime}}_{{a}_{ij}}^{l},{{\eta }^{\prime}}_{{a}_{ij}}^{\mathpzc{u}}\right]\right)$ be a family of IVPFSVs and ${\Delta }^{\prime}=\left(\left[{\lambda }^{{\prime}l},{\lambda }^{\mathcal{^{\prime}}\mathpzc{u}}\right],\left[{\eta }^{{\prime}l},{\eta }^{\mathcal{^{\prime}}\mathpzc{u}}\right]\right)$ be an IVPFSV. Then,

$$IVPFSEHWG\left({\Delta }_{{a}_{11}}^{\prime}{{\otimes}}_{\varepsilon }{\Delta }^{\prime}, {\Delta }_{{a}_{12}}^{\prime}{{\otimes}}_{\varepsilon }{\Delta }^{\prime},\dots ,{\Delta }_{{a}_{nm}}^{\prime}{{\otimes}}_{\varepsilon }{\Delta }^{\prime}\right)$$

$$= IVPFSEHWG\left({\Delta }_{{a}_{11}}^{\prime},{\Delta }_{{a}_{12}}^{{{\prime}}},\dots \dots ,{\Delta }_{{a}_{nm}}^{\prime}\right){{\otimes}}_{\varepsilon }{\Delta }^{\prime}$$

## The evaluation based on the distance from the average solution (EDAS) model using Einstein-ordered and hybrid AOs

The interaction of experts from various fields frequently results in an abundance of thoughts on the most effective operation plans. To organize the uncertain circumstances and their findings, implementing IVPFSS ratings is necessary. This research introduces the Einstein-ordered and hybrid AOs within the IVPFSS context. Also, it advocates the execution of the EDAS approach, which draws on these AOs, to solve the MAGDM issue in FSCM. The EDAS method incorporates two essential measures, the PDA and NDA, to precisely assess variations across multiple alternatives. This methodology is adequate for managing MAGDM obstacles, despite the desire for a detailed analysis of both ideal and imperfect decisions. The consequent section explores the mathematical basis supporting this strategy.

Further instructions for executing the specified method are provided:

**Step 1:** A team of decision experts $\ss =\left\{{\ss }_{1},{\ss }_{2},\dots \dots {\ss }_{s}\right\}$ is entrusted with expressing their preferences among a collection of alternatives $\bar{\mathrm{U}}=\left\{{\bar{\mathrm{U}}}_{1},{\bar{\mathrm{U}}}_{2},\dots \dots {\bar{\mathrm{U}}}_{k}\right\}$. In MAGDM circumstances based on attributes set $\mathfrak{R}=\left\{{\mathfrak{R}}_{1},{\mathfrak{R}}_{2},\dots \dots {\mathfrak{R}}_{z}\right\}$. Let $\partial ={\left({\partial }_{1}, {\partial }_{2},\dots , {\partial }_{n}\right)}^{T}$ and $\Upsilon={\left({\Upsilon}_{1}, {\Upsilon}_{2},\dots , {\Upsilon}_{n}\right)}^{T}$ be the weights of professionals and criteria with ${\partial }_{i}>0$ and $\sum_{i=1}^{n}{\partial }_{i}=1$, ${\Upsilon}_{j}>0$ and $\sum_{j=1}^{m}{\Upsilon}_{j}=1$. The decision experts undertake decision matrices using IVPFSNs, such as ${\Xi }_{{\mathrm{a}}_{\mathrm{ij}}}^{p}=\left({\lambda }_{{a}_{ij}}^{(p)}, {\eta }_{{a}_{ij}}^{(p)}\right)$,

where ${\lambda }_{{a}_{ij}}^{(p)}=\left[{\lambda }_{{a}_{ij}}^{l},{\lambda }_{{a}_{ij}}^{\mathpzc{u}}\right]$, ${\eta }_{{a}_{ij}}^{(p)}=\left[{\eta }_{{a}_{ij}}^{l},{\eta }_{{a}_{ij}}^{\mathpzc{u}}\right]$, and $0\le {\lambda }_{{a}_{ij}}^{l},{\lambda }_{{a}_{ij}}^{\mathpzc{u}}, {\eta }_{{a}_{ij}}^{l},{\eta }_{{a}_{ij}}^{\mathpzc{u}}\le 1$ and ${\left({\lambda }_{{a}_{ij}}^{\mathpzc{u}}\right)}^{2}+{\left({\eta }_{{a}_{ij}}^{\mathpzc{u}}\right)}^{2}\le 1$, $\forall i, j$. The following describes the interaction between IVPFSNs and experts’ assessments of the set of alternatives:

$${\left[{\Xi }_{{a}_{ij}}^{p}\right]}_{n\times m}$$

$$=\begin{array}{c}{\ss }_{1}\\ {\ss }_{2}\\ \vdots \\ {\ss }_{n}\end{array}\left(\begin{array}{cccc}\left(\left[{\lambda }_{{a}_{11}}^{l},{\lambda }_{{a}_{11}}^{\mathpzc{u}}\right], \left[{\eta }_{{a}_{11}}^{l},{\eta }_{{a}_{11}}^{\mathpzc{u}}\right]\right)& \left(\left[{\lambda }_{{a}_{12}}^{l},{\lambda }_{{a}_{12}}^{\mathpzc{u}}\right], \left[{\eta }_{{a}_{12}}^{l},{\eta }_{{a}_{12}}^{\mathpzc{u}}\right]\right)& \cdots & \left(\left[{\lambda }_{{a}_{1m}}^{l},{\lambda }_{{a}_{1m}}^{\mathpzc{u}}\right], \left[{\eta }_{{a}_{1m}}^{l},{\eta }_{{a}_{1m}}^{\mathpzc{u}}\right]\right)\\ \left(\left[{\lambda }_{{a}_{21}}^{l},{\lambda }_{{a}_{21}}^{\mathpzc{u}}\right], \left[{\eta }_{{a}_{21}}^{l},{\eta }_{{a}_{21}}^{\mathpzc{u}}\right]\right)& \left(\left[{\lambda }_{{a}_{22}}^{l},{\lambda }_{{a}_{22}}^{\mathpzc{u}}\right], \left[{\eta }_{{a}_{22}}^{l},{\eta }_{{a}_{22}}^{\mathpzc{u}}\right]\right)& \cdots & \left(\left[{\lambda }_{{a}_{2m}}^{l},{\lambda }_{{a}_{2m}}^{\mathpzc{u}}\right], \left[{\eta }_{{a}_{2m}}^{l},{\eta }_{{a}_{2m}}^{\mathpzc{u}}\right]\right)\\ \vdots & \vdots & \vdots & \vdots \\ \left(\left[{\lambda }_{{a}_{n1}}^{l},{\lambda }_{{a}_{n1}}^{\mathpzc{u}}\right], \left[{\eta }_{{a}_{n1}}^{l},{\eta }_{{a}_{n1}}^{\mathpzc{u}}\right]\right)& \left(\left[{\lambda }_{{a}_{n1}}^{l},{\lambda }_{{a}_{n1}}^{\mathpzc{u}}\right], \left[{\eta }_{{a}_{n1}}^{l},{\eta }_{{a}_{n1}}^{\mathpzc{u}}\right]\right)& \cdots & \left(\left[{\lambda }_{{a}_{nm}}^{l},{\lambda }_{{a}_{nm}}^{\mathpzc{u}}\right], \left[{\eta }_{{a}_{nm}}^{l},{\eta }_{{a}_{nm}}^{\mathpzc{u}}\right]\right)\end{array}\right)$$

**Step 2:** The study of matrix ${\left[{\Xi }_{{a}_{ij}}^{p}\right]}_{n\times m}$ is carried out by splitting the criteria into two separate groups: cost-related attributes and benefit-related attributes. If the factors are identical, normalization is not necessary. Still, if the decision matrix incorporates divergent parameter forms, normalization must be executed to standardize them. The normalization mechanism is described in Eq. 7.

7 $${R}_{ij}=\left\{\begin{array}{c}{\left({\Xi }_{{a}_{ij}}^{p}\right)}^{c}= \left(\left[{\eta }_{{a}_{ij}}^{l},{\eta }_{{a}_{ij}}^{\mathpzc{u}}\right], \left[{\lambda }_{{a}_{ij}}^{l},{\lambda }_{{a}_{ij}}^{\mathpzc{u}}\right]\right)\\ {\Xi }_{{a}_{ij}}^{p}= \left(\left[{\lambda }_{{a}_{ij}}^{l},{\lambda }_{{a}_{ij}}^{\mathpzc{u}}\right], \left[{\eta }_{{a}_{ij}}^{l},{\eta }_{{a}_{ij}}^{\mathpzc{u}}\right]\right)\end{array}\right.$$

**Step 3:** Determine the ordered matrix for every alternate.

**Step 4:** Expert opinions and assessments are merged using intellectual selection evidence and assessed directly by experts through operators such as IVPFSEOWA, IVPFSEHWA, IVPFSEOWG, and IVPFSEHWG. This technique derives all desired preferences for alternatives from the provided parameters.

$${\left[{\Xi }_{{a}_{ij}}\right]}_{n\times m}={\left(\left[{\lambda }_{{a}_{ij}}^{l},{\lambda }_{{a}_{ij}}^{\mathpzc{u}}\right],\left[{\eta }_{{a}_{ij}}^{l},{\eta }_{{a}_{ij}}^{\mathpzc{u}}\right]\right)}_{n\times m}$$

**Step 5:** Compute the average solution matrix by using Eq. 8.

8 $$\text {\AA} {S}_{h}={\left[\mathrm{\AA} {S}_{h}\right]}_{1\times m}=\frac{1}{n}\stackrel{n}{\underset{i=1}{\oplus }}\left({\Xi }_{{a}_{ij}}\right)$$

**Step 6:** Calculate the PDA and NDA by determining ${D}_{b}$ and ${D}_{c}$ as an accumulation of beneficial and adverse factors, correspondingly. Afterwards, compute the PDAS and NDAS matrices assuming a particular technique:

$$PDA={\left[{\rho }_{{a}_{ij}}\right]}_{n\times m}$$**,** $$NDA={\left[{\gamma }_{{a}_{ij}}\right]}_{n\times m}$$, where

9 $${\rho }_{{a}_{ij}}=\left\{\begin{array}{c}\frac{max\left(0,\left({\Xi }_{{a}_{ij}}-\mathrm{\AA} {S}_{h}\right)\right)}{S\left( \text {\AA} {S}_{h}\right)} for i=1\left(1\right) n, j=1\left(1\right) m and {\mathfrak{R}}_{{\boldsymbol{z}}}\in {D}_{b} \\ \begin{array}{c}\frac{max\left(0,\left(\text {\AA} {S}_{h}-{\Xi }_{{a}_{ij}}\right)\right)}{S\left(\text {\AA }{S}_{h}\right)}\end{array}, for i=1\left(1\right) n, j=1\left(1\right) m and {\mathfrak{R}}_{{\boldsymbol{z}}}\in {D}_{c}\end{array}\right.$$

and

10 $${\gamma }_{{a}_{ij}}=\left\{\begin{array}{c}\frac{max\left(0,\left( \mathrm{\AA} {S}_{h}-{\Xi }_{{a}_{ij}}\right)\right)}{S\left(\mathrm{\AA} {S}_{h}\right)} for i=1\left(1\right)n, j=1\left(1\right)m and {\mathfrak{R}}_{{\boldsymbol{z}}}\in {D}_{b} \\ \begin{array}{c}\frac{max\left(0,\left({\Xi }_{{a}_{ij}}- \mathrm{\AA} {S}_{h}\right)\right)}{S\left( \mathrm{\AA} {S}_{h}\right)}\end{array} for i=1\left(1\right)n, j=1\left(1\right)m and {\mathfrak{R}}_{{\boldsymbol{z}}}\in {D}_{c}\end{array}\right.$$

**Step 7:** Determine the ${\rm P}_{\tau }$ and ${Q}_{\tau }$ for the IVPFSEHWA and IVPFSEOWA operators. Where, ${\rm P}_{\tau }$ and ${Q}_{\tau }$ be the positive and negative weighted distances, respectively.

11 $${\rm P}_{\tau }=\stackrel{m}{\underset{j=1}{\oplus }}{\Upsilon}_{j}\left(\stackrel{n}{\underset{i=1}{\oplus }}{\partial }_{i}{\rho }_{{a}_{ij}}\right)$$

12 $${Q}_{\tau }=\stackrel{m}{\underset{j=1}{\oplus }}{\Upsilon}_{j}\left(\stackrel{n}{\underset{i=1}{\oplus }}{\partial }_{i}{\gamma }_{{a}_{ij}}\right)$$

or with the operators, IVPFSEHWG and IVPFSEOWG defined as:

13 $${\rm P}_{\tau }={\left(\stackrel{m}{\underset{j=1}{{\otimes}}}{\left(\stackrel{n}{\underset{i=1}{{\otimes}}}{\rho }_{{a}_{ij}}\right)}^{{\partial }_{i}}\right)}^{{\Upsilon}_{j}}$$

14 $${Q}_{\tau }={\left(\stackrel{m}{\underset{j=1}{{\otimes}}}{\left(\stackrel{n}{\underset{i=1}{{\otimes}}}{\gamma }_{{a}_{ij}}\right)}^{{\partial }_{i}}\right)}^{{\Upsilon}_{j}}$$

where ${\Upsilon}_{j}>0,{ \partial }_{i}>0$, such as $\sum_{j=1}^{m}{\Upsilon}_{j}=1$ and.

**Step 8:** Using Eq. 15 and Eq. 16, obtain the normalized values of ${\rm P}_{\tau }$ and ${Q}_{\tau }$, $\tau =\mathrm{1,2},\dots \dots m.$

15 $$N{\rm P}_{\tau }=\frac{{\rm P}_{\tau }}{max\left({\rm P}_{1},{\rm P}_{2},\dots \dots {\rm P}_{m}\right)}$$

16 $$N{Q}_{\tau }=1-\frac{{Q}_{\tau }}{max\left({Q}_{1},{Q}_{2},\dots \dots {Q}_{m}\right)}$$

**Step 9:** Employ Eq. 17 to determine the appraisal score $I$, such as $I\in \left[\mathrm{0,1}\right].$

17 $$I=\frac{1}{2}\left(N{\rm P}_{\tau }\oplus N{Q}_{\tau }\right)$$

**Step 10:** The outcomes are organized in terms of their overall opinions, from most favourable to least favourable. The alternatives with the most points will be considered particularly helpful, while the selection with the lowest score will be evaluated as the least favoured.

**Fig. 1:**
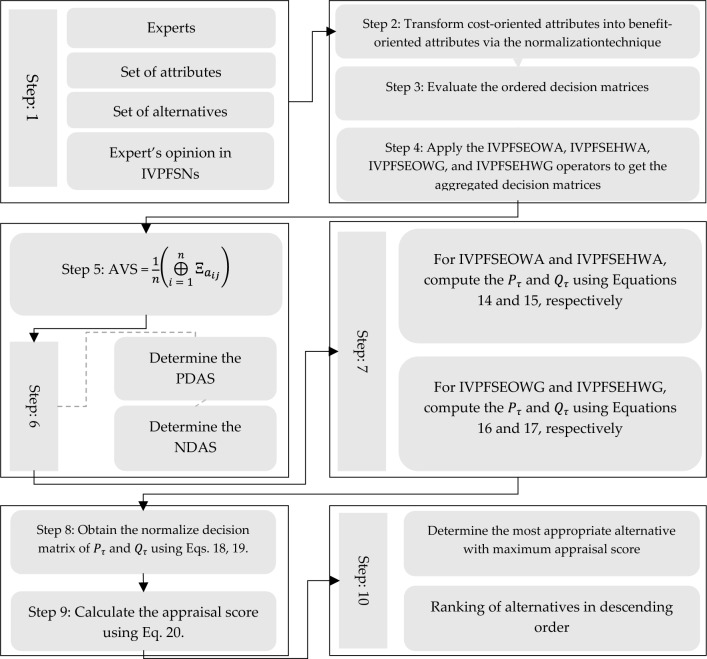
Flow chart of the developed EDAS method.

Figure 1 outlines the recommended strategy.

## Application of the proposed EDAS method in sustainable food suppliers

This section will evaluate performance to determine the best approach for finding a sustainable food supplier.

### Alternative description of sustainable food suppliers

Finding the most sustainable food suppliers is challenging and heavily relies on your location, the accessibility of the products, and, last but not least, your personal taste. Nevertheless, the following ranking of five outstanding organic food companies is listed for their commitment to social responsibility, environmental protection, and great quality products:

#### *Patagonia provisions*^5^

Patagonia Provisions, the environmental and farming rejuvenation efforts of the outdoor product supplier Patagonia, have gained worldwide recognition for their continuous dedication. In fact, this organization provides a range of agricultural products through sustainable means that are not only genuine but also intentionally grown, including grains, meats, fish, and basic products.

#### *Thrive market*^6^

Thrive market is an internet grocery store that offers easy access to local, non-GMO, and organic food items.

#### *Organic valley*^4^

The producers of Organic Valley are registered organic producers who act as a unified team, driven by a sense of social responsibility and mission to organic agriculture.

#### *Whole foods market*^7^

Whole Foods Market is a well-known retail store, providing customers with a wide range of organic, locally grown, and even professionally grown products. It not only invites local farmers and businesses to participate but also financially supports organizations that strive for openness and corporate responsibility in the environmental sector.

#### *Dr. Bronner’s*^2^

The organic and transparent food collection is an important part of Dr. Bronner’s, a company known for its health and hygiene products. Dr. Bronner’s integrates sustainable farming practices that are profitable for both consumers and the earth by obtaining its products directly from local farmers.

The alternative profiles in Section "Alternative description of sustainable food suppliers" were summarized to reflect recurring sustainability practices emphasized in the food supply-chain literature. These practices were then operationalized into measurable evaluation criteria (Table 1). Specifically, the aspects highlighted across the alternatives, social contribution and labor practices, economic capability and innovation orientation, and environmental footprint and waste management were mapped into the six criteria used for evaluation.

**Table 1:** Criteria description for sustainable food supplier selection.

Principle aspects	Criteria	Definition
Social sustainability	Community engagement^5,7^	By investing in local projects, supporting grassroots efforts, and cooperating with local groups, suppliers build relationships with communities to promote economic development in the region
	Worker health and safety^39^	The safety and health of employees come first through protective measures, such as providing training, personal protective equipment, good working conditions, medical insurance, and regular inspections
Economic sustainability	Financial ability^5,6^	Suppliers are developing very competitive pricing controls for workers, manufacturers, and distributors to ensure adequate supply and trade, thereby improving living standards and stabilizing agriculture
	Support for innovation and entrepreneurship^24,58^	By providing scientific and technical support and promoting environmental protection methods, suppliers help the food industry become more creative and entrepreneurial
Environmental sustainability	Carbon footprint reduction^38,53^	During production, processing, transportation, and storage, suppliers are committed to improving logistics, adopting regional purchasing preferences, and implementing emission-reduction plans throughout the food supply chain
	Waste reduction and recycling^53,58^	Suppliers’ efforts to reduce waste and promote the recycling and composting of organic materials in various ways

### Problem definition

The IVPFSS not only attracts studies that are suitable but also engages with them greatly, and hopefully, the development of prescriptions for the problem of sustainable food supplier selection will be the result of this engagement. Then, based on a previous study and the experts’ comments, we specified the parameters for sustainable food sources. The identification of eco-friendly procurement hindrances classified the parameters into three main categories: social, economic, and environmental sustainability. Subsequently, we created an eligibility mapping that zeroes in on the different factors that affect our choices for green manufacturers and the characteristics of food suppliers. The necessary information to find a trustworthy food supplier is presented in Table 1. Using the parameters provided in Table 1, we assessed five possible proposals (refer to subsection "Alternative description of sustainable food suppliers") to identify the best organic food supplier within the IVPFSS framework.

### Selection of a sustainable food supplier

Given that finding a suitable substitute is challenging in itself, identifying a fully feasible natural substitute is even more challenging. In this case study, a panel of five experts was selected to ensure that all fields were covered and the decision-making was reliable. These experts come from a wide range of fields, including decision science, environmental management, logistics/procurement, food supply chain management, and sustainable operation. Each expert has 8 to 12 years of relevant academic or industry experience. Before scoring, experts were informed of the definitions of each evaluation standard (Table 1) and the meaning of the scoring scale based on IVPFSS, to ensure everyone understood it consistently. Each expert independently uses IVPFSNs to evaluate five candidate suppliers. Then, using the proposed Einstein sequence and Einstein mixing operator, their evaluations are integrated, and experts’ weights are assigned based on experience and domain relevance to improve transparency and repeatability.

This study presents five different yet practical options for good food sources. The issue here is to identify the best suppliers and develop a management structure for the selected organic food suppliers. The rationale for the various sustainable food providers is provided in subsection "Alternative description of sustainable food suppliers" as follows: 

 .

$$\overline{{\mathrm{U}}} = \left\{ \begin{gathered} \overline{{\mathrm{U}}} _{1} = {\text{Patagonia provisions}},\overline{{\mathrm{U}}} _{2} = {\text{Thrive market}},\overline{{\mathrm{U}}} _{3} = {\text{Organic Valley}}, \hfill \\ \overline{{\mathrm{U}}} _{4} = {\text{Whole Foods Market}},\overline{{\mathrm{U}}} _{5} = {\mathrm{Dr}}.Bronner^{\prime } {\mathrm{s}} \hfill \\ \end{gathered} \right\}$$

The variables’ weights help identify sustainable food sources, enabling professionals to assess their choices among five possibilities. From this point of view, we evaluate the next six parameters: $\mathfrak{R}=\{{\mathfrak{R}}_{1}=\text{Community engagement}$, ${\mathfrak{R}}_{2}=\text{Worker health and safety}$, ${\mathfrak{R}}_{3}=\text{Financial ability}$, ${\mathfrak{R}}_{4}=\text{Support for innovation and entrepreneurship}$, and ${\mathfrak{R}}_{5}=\text{Carbon footprint reduction}$, ${\mathfrak{R}}_{6}=$ Waste reduction and recycling} are selection criteria with weights $\Upsilon=(0.18, \mathrm{0.12,0.2,0.15,0.2}, 0.15),$ respectively. of the sustainable food supplier selection problem.

Let $\ss =\left\{{\ss }_{1}, {\ss }_{2}, {\ss }_{3}, {\ss }_{4}, {\ss }_{5}\right\}$ be a group consisting of five specialists with weights $\partial =(\mathrm{0.21,0.19,0.22}, 0.18, 0.20).$ Experts ${\ss }_{1}$, ${\ss }_{2}$, and ${\ss }_{4}$ are from the industry, with their positions as Procurement manager, Quality assurance specialist, and Supply chain manager, and 15 years of experience. These three experts have their own area of expertise, such as Food sourcing and supplier evaluation, Food safety and quality management, and Logistics and supplier performance. Whereas the other two experts ${\ss }_{3}$ and ${\ss }_{5}$ are from academia with expertise in Environmental sustainability, green supply chains, and Sustainable supply chain decision analysis, with 15 years of experience. Each expert assessed the suppliers using predefined criteria based on IVPFSS information. After accumulating the individual assessment environments, the decisions were structured into IVPFSNs and consequently managed using the established EDAS technique. The experts’ assessments are aggregated using the proposed Einstein-based interval-valued Pythagorean fuzzy soft aggregation operators to obtain the collective decision matrix for the consequent EDAS method.

**Fig. 2:**
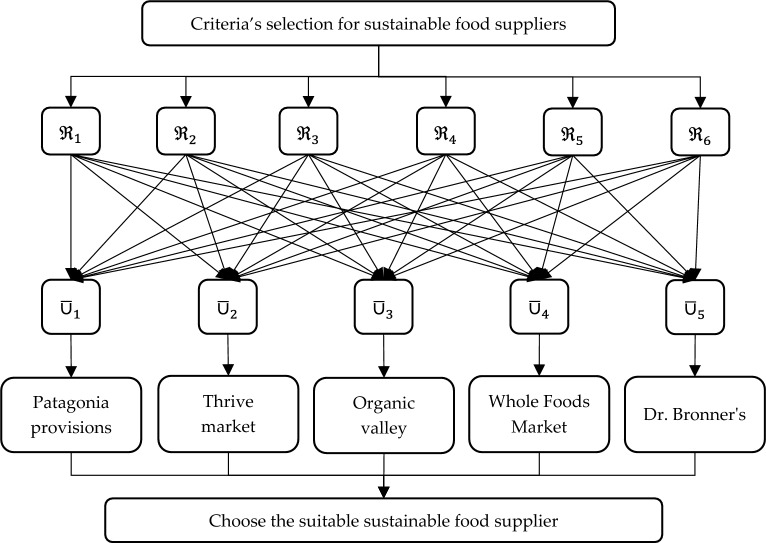
Selection process of the sustainable food supplier.

Where ${\mathfrak{R}}_{2}=\text{Worker health and safety}$, ${\mathfrak{R}}_{3}=\text{Financial ability}$ and ${\mathfrak{R}}_{6}=$ Waste reduction and recycling are cost-like parameters, while the remaining ones are of benefit type. The classification of parameters to be evaluated when choosing a sustainable food provider is illustrated in Fig. 2.

Given the abovementioned criteria, our current approach suggests identifying the most optimal option. The decision mechanism generates substantial attention to safeguarding the global ecological balance and the well-being of individuals and crops. The primary focus of sustainable food supplier selection strategies is to ensure both social and economic viability. Evaluators provided their assessments as IVPFSNs for each sustainable food provider option specified in section "Alternative description of sustainable food suppliers". The Einstein-ordered and Einstein hybrid AOs described in Section "The evaluation based on the distance from the average solution (EDAS) model using Einstein-ordered and hybrid AOs" are integrated into the EDAS strategy to determine the optimal sustainable food source decision. The subsequent section presents the procedure for selecting the most suitable sustainable food supplier utilizing the recommended EDAS technique.

Step 1: The team of professionals meticulously evaluated all the documented food supplier procedures and provided their feedback. Table 2 provides a concise overview of experts’ interests, expressed as IVPFSNs, for each available option.

**Table 2:** The expert’s opinion on each alternative IVPFSV based on the considered factors.

${\bar{\mathrm{U}}}_{1}$	${\mathfrak{R}}_{1}$	${\mathfrak{R}}_{2}$	${\mathfrak{R}}_{3}$	${\mathfrak{R}}_{4}$	${\mathfrak{R}}_{5}$	${\mathfrak{R}}_{6}$
${\boldsymbol{\ss }}_{1}$	$\left(\left[\mathrm{0.6,0.8}\right],\left[\mathrm{0.3,0.5}\right]\right)$	$\left(\left[\mathrm{0.3,0.8}\right],\left[\mathrm{0.4,0.6}\right]\right)$	$\left(\left[\mathrm{0.4,0.6}\right],\left[\mathrm{0.3,0.7}\right]\right)$	$\left(\left[\mathrm{0.1,0.8}\right],\left[\mathrm{0.4,0.6}\right]\right)$	$\left(\left[\mathrm{0.3,0.6}\right],\left[\mathrm{0.1,0.7}\right]\right)$	$\left(\left[\mathrm{0.4,0.5}\right],\left[\mathrm{0.6,0.8}\right]\right)$
${\boldsymbol{\ss }}_{2}$	$\left(\left[\mathrm{0.2,0.3}\right],\left[\mathrm{0.6,0.9}\right]\right)$	$\left(\left[\mathrm{0.4,0.5}\right],\left[\mathrm{0.2,0.8}\right]\right)$	$\left(\left[\mathrm{0.2,0.5}\right],\left[\mathrm{0.5,0.7}\right]\right)$	$\left(\left[\mathrm{0.3,0.6}\right],\left[\mathrm{0.4,0.8}\right]\right)$	$\left(\left[\mathrm{0.1,0.2}\right],\left[\mathrm{0.5,0.8}\right]\right)$	$\left(\left[\mathrm{0.6,0.6}\right],\left[\mathrm{0.5,0.7}\right]\right)$
${\boldsymbol{\ss }}_{3}$	$\left(\left[\mathrm{0.6,0.7}\right],\left[\mathrm{0.2,0.7}\right]\right)$	$\left(\left[\mathrm{0.5,0.7}\right],\left[\mathrm{0.4,0.7}\right]\right)$	$\left(\left[\mathrm{0.4,0.5}\right],\left[\mathrm{0.1,0.3}\right]\right)$	$\left(\left[\mathrm{0.4,0.7}\right],\left[\mathrm{0.3,0.5}\right]\right)$	$\left(\left[\mathrm{0.7,0.8}\right],\left[\mathrm{0.4,0.6}\right]\right)$	$\left(\left[\mathrm{0.6,0.8}\right],\left[\mathrm{0.3,0.5}\right]\right)$
${\boldsymbol{\ss }}_{4}$	$\left(\left[\mathrm{0.1,0.4}\right],\left[\mathrm{0.4,0.6}\right]\right)$	$\left(\left[\mathrm{0.3,0.6}\right],\left[\mathrm{0.1,0.7}\right]\right)$	$\left(\left[\mathrm{0.6,0.6}\right],\left[\mathrm{0.3,0.7}\right]\right)$	$\left(\left[\mathrm{0.2,0.3}\right],\left[\mathrm{0.1,0.8}\right]\right)$	$\left(\left[\mathrm{0.4,0.6}\right],\left[\mathrm{0.5,0.7}\right]\right)$	$\left(\left[\mathrm{0.2,0.5}\right],\left[\mathrm{0.5,0.7}\right]\right)$
${\boldsymbol{\ss }}_{5}$	$\left(\left[\mathrm{0.7,0.8}\right],\left[\mathrm{0.3,0.4}\right]\right)$	$\left(\left[\mathrm{0.3,0.7}\right],\left[\mathrm{0.4,0.6}\right]\right)$	$\left(\left[\mathrm{0.4,0.8}\right],\left[\mathrm{0.2,0.6}\right]\right)$	$\left(\left[\mathrm{0.3,0.8}\right],\left[\mathrm{0.5,0.6}\right]\right)$	$\left(\left[\mathrm{0.1,0.8}\right],\left[\mathrm{0.4,0.5}\right]\right)$	$\left(\left[\mathrm{0.2,0.3}\right],\left[\mathrm{0.6,0.8}\right]\right)$
$${\bar{\mathrm{U}}}_{2}$$						
${\boldsymbol{\ss }}_{1}$	$\left(\left[\mathrm{0.4,0.7}\right],\left[\mathrm{0.2,0.6}\right]\right)$	$\left(\left[\mathrm{0.4,0.7}\right],\left[\mathrm{0.4,0.5}\right]\right)$	$\left(\left[\mathrm{0.4,0.5}\right],\left[\mathrm{0.6,0.8}\right]\right)$	$\left(\left[\mathrm{0.4,0.7}\right],\left[\mathrm{0.1,0.6}\right]\right)$	$\left(\left[\mathrm{0.6,0.7}\right],\left[\mathrm{0.2,0.6}\right]\right)$	$\left(\left[\mathrm{0.5,0.7}\right],\left[\mathrm{0.3,0.6}\right]\right)$
${\boldsymbol{\ss }}_{2}$	$\left(\left[\mathrm{0.6,0.8}\right],\left[\mathrm{0.3,0.4}\right]\right)$	$\left(\left[\mathrm{0.2,0.6}\right],\left[\mathrm{0.5,0.8}\right]\right)$	$\left(\left[\mathrm{0.6,0.6}\right],\left[\mathrm{0.5,0.7}\right]\right)$	$\left(\left[\mathrm{0.1,0.5}\right],\left[\mathrm{0.1,0.8}\right]\right)$	$\left(\left[\mathrm{0.4,0.7}\right],\left[\mathrm{0.6,0.6}\right]\right)$	$\left(\left[\mathrm{0.4,0.5}\right],\left[\mathrm{0.3,0.7}\right]\right)$
${\boldsymbol{\ss }}_{3}$	$\left(\left[\mathrm{0.1,0.8}\right],\left[\mathrm{0.1,0.5}\right]\right)$	$\left(\left[\mathrm{0.3,0.8}\right],\left[\mathrm{0.3,0.4}\right]\right)$	$\left(\left[\mathrm{0.6,0.8}\right],\left[\mathrm{0.3,0.5}\right]\right)$	$\left(\left[\mathrm{0.3,0.4}\right],\left[\mathrm{0.3,0.4}\right]\right)$	$\left(\left[\mathrm{0.3,0.5}\right],\left[\mathrm{0.4,0.8}\right]\right)$	$\left(\left[\mathrm{0.2,0.6}\right],\left[\mathrm{0.3,0.5}\right]\right)$
${\boldsymbol{\ss }}_{4}$	$\left(\left[\mathrm{0.3,0.8}\right],\left[\mathrm{0.4,0.6}\right]\right)$	$\left(\left[\mathrm{0.4,0.8}\right],\left[\mathrm{0.4,0.6}\right]\right)$	$\left(\left[\mathrm{0.2,0.5}\right],\left[\mathrm{0.5,0.7}\right]\right)$	$\left(\left[\mathrm{0.2,0.4}\right],\left[\mathrm{0.5,0.8}\right]\right)$	$\left(\left[\mathrm{0.3,0.6}\right],\left[\mathrm{0.5,0.7}\right]\right)$	$\left(\left[\mathrm{0.3,0.4}\right],\left[\mathrm{0.4,0.6}\right]\right)$
${\boldsymbol{\ss }}_{5}$	$\left(\left[\mathrm{0.3,0.4}\right],\left[\mathrm{0.3,0.5}\right]\right)$	$\left(\left[\mathrm{0.3,0.9}\right],\left[\mathrm{0.1,0.2}\right]\right)$	$\left(\left[\mathrm{0.2,0.3}\right],\left[\mathrm{0.6,0.8}\right]\right)$	$\left(\left[\mathrm{0.1,0.4}\right],\left[\mathrm{0.5,0.8}\right]\right)$	$\left(\left[\mathrm{0.4,0.8}\right],\left[\mathrm{0.4,0.6}\right]\right)$	$\left(\left[\mathrm{0.5,0.8}\right],\left[\mathrm{0.3,0.5}\right]\right)$
$${\bar{\mathrm{U}}}_{3}$$						
${\boldsymbol{\ss }}_{1}$	$\left(\left[\mathrm{0.5,0.7}\right],\left[\mathrm{0.3,0.6}\right]\right)$	$\left(\left[\mathrm{0.4,0.5}\right],\left[\mathrm{0.2,0.6}\right]\right)$	$\left(\left[\mathrm{0.5,0.9}\right],\left[\mathrm{0.3,0.4}\right]\right)$	$\left(\left[\mathrm{0.4,0.6}\right],\left[\mathrm{0.3,0.4}\right]\right)$	$\left(\left[\mathrm{0.2,0.5}\right],\left[\mathrm{0.5,0.8}\right]\right)$	$\left(\left[\mathrm{0.3,0.8}\right],\left[\mathrm{0.4,0.6}\right]\right)$
${\boldsymbol{\ss }}_{2}$	$\left(\left[\mathrm{0.4,0.5}\right],\left[\mathrm{0.3,0.7}\right]\right)$	$\left(\left[\mathrm{0.5,0.5}\right],\left[\mathrm{0.4,0.6}\right]\right)$	$\left(\left[\mathrm{0.3,0.5}\right],\left[\mathrm{0.4,0.6}\right]\right)$	$\left(\left[\mathrm{0.1,0.6}\right],\left[\mathrm{0.3,0.8}\right]\right)$	$\left(\left[\mathrm{0.4,0.6}\right],\left[\mathrm{0.6,0.8}\right]\right)$	$\left(\left[\mathrm{0.4,0.5}\right],\left[\mathrm{0.2,0.8}\right]\right)$
${\boldsymbol{\ss }}_{3}$	$\left(\left[\mathrm{0.2,0.6}\right],\left[\mathrm{0.3,0.5}\right]\right)$	$\left(\left[\mathrm{0.5,0.6}\right],\left[\mathrm{0.3,0.7}\right]\right)$	$\left(\left[\mathrm{0.3,0.8}\right],\left[\mathrm{0.2,0.4}\right]\right)$	$\left(\left[\mathrm{0.5,0.6}\right],\left[\mathrm{0.1,0.7}\right]\right)$	$\left(\left[\mathrm{0.5,0.7}\right],\left[\mathrm{0.3,0.6}\right]\right)$	$\left(\left[\mathrm{0.5,0.7}\right],\left[\mathrm{0.4,0.7}\right]\right)$
${\boldsymbol{\ss }}_{4}$	$\left(\left[\mathrm{0.3,0.4}\right],\left[\mathrm{0.4,0.6}\right]\right)$	$\left(\left[\mathrm{0.2,0.5}\right],\left[\mathrm{0.2,0.8}\right]\right)$	$\left(\left[\mathrm{0.3,0.6}\right],\left[\mathrm{0.4,0.8}\right]\right)$	$\left(\left[\mathrm{0.4,0.7}\right],\left[\mathrm{0.6,0.7}\right]\right)$	$\left(\left[\mathrm{0.4,0.6}\right],\left[\mathrm{0.1,0.5}\right]\right)$	$\left(\left[\mathrm{0.3,0.6}\right],\left[\mathrm{0.1,0.7}\right]\right)$
${\boldsymbol{\ss }}_{5}$	$\left(\left[\mathrm{0.5,0.8}\right],\left[\mathrm{0.3,0.5}\right]\right)$	$\left(\left[\mathrm{0.6,0.9}\right],\left[\mathrm{0.3,0.4}\right]\right)$	$\left(\left[\mathrm{0.8,0.9}\right],\left[\mathrm{0.1,0.4}\right]\right)$	$\left(\left[\mathrm{0.3,0.4}\right],\left[\mathrm{0.3,0.4}\right]\right)$	$\left(\left[\mathrm{0.4,0.7}\right],\left[\mathrm{0.3,0.6}\right]\right)$	$\left(\left[\mathrm{0.3,0.7}\right],\left[\mathrm{0.4,0.6}\right]\right)$
$${\bar{\mathrm{U}}}_{4}$$						
${\boldsymbol{\ss }}_{1}$	$\left(\left[\mathrm{0.5,0.8}\right],\left[\mathrm{0.3,0.6}\right]\right)$	$\left(\left[\mathrm{0.5,0.6}\right],\left[\mathrm{0.2,0.4}\right]\right)$	$\left(\left[\mathrm{0.4,0.6}\right],\left[\mathrm{0.5,0.7}\right]\right)$	$\left(\left[\mathrm{0.6,0.8}\right],\left[\mathrm{0.2,0.5}\right]\right)$	$\left(\left[\mathrm{0.5,0.7}\right],\left[\mathrm{0.4,0.6}\right]\right)$	$\left(\left[\mathrm{0.3,0.6}\right],\left[\mathrm{0.1,0.7}\right]\right)$
${\boldsymbol{\ss }}_{2}$	$\left(\left[\mathrm{0.3,0.5}\right],\left[\mathrm{0.5,0.7}\right]\right)$	$\left(\left[\mathrm{0.5,0.7}\right],\left[\mathrm{0.5,0.7}\right]\right)$	$\left(\left[\mathrm{0.6,0.8}\right],\left[\mathrm{0.3,0.5}\right]\right)$	$\left(\left[\mathrm{0.1,0.7}\right],\left[\mathrm{0.5,0.7}\right]\right)$	$\left(\left[\mathrm{0.3,0.5}\right],\left[\mathrm{0.4,0.5}\right]\right)$	$\left(\left[\mathrm{0.1,0.2}\right],\left[\mathrm{0.5,0.8}\right]\right)$
${\boldsymbol{\ss }}_{3}$	$\left(\left[\mathrm{0.5,07}\right],\left[\mathrm{0.4,0.5}\right]\right)$	$\left(\left[\mathrm{0.2,0.8}\right],\left[\mathrm{0.1,0.6}\right]\right)$	$\left(\left[\mathrm{0.5,0.8}`\right],\left[\mathrm{0.5,0.6}\right]\right)$	$\left(\left[\mathrm{0.4,0.8}\right],\left[\mathrm{0.6,0.6}\right]\right)$	$\left(\left[\mathrm{0.1,0.4}\right],\left[\mathrm{0.7,0.9}\right]\right)$	$\left(\left[\mathrm{0.7,0.8}\right],\left[\mathrm{0.4,0.6}\right]\right)$
${\boldsymbol{\ss }}_{4}$	$\left(\left[\mathrm{0.2,0.6}\right],\left[\mathrm{0.5,0.8}\right]\right)$	$\left(\left[\mathrm{0.2,0.4}\right],\left[\mathrm{0.6,0.7}\right]\right)$	$\left(\left[\mathrm{0.1,0.6}\right],\left[\mathrm{0.6,0.8}\right]\right)$	$\left(\left[\mathrm{0.3,0.7}\right],\left[\mathrm{0.5,0.6}\right]\right)$	$\left(\left[\mathrm{0.5,0.8}\right],\left[\mathrm{0.4,0.5}\right]\right)$	$\left(\left[\mathrm{0.4,0.6}\right],\left[\mathrm{0.5,0.7}\right]\right)$
${\boldsymbol{\ss }}_{5}$	$\left(\left[0.5.0.5\right],\left[\mathrm{0.4,0.8}\right]\right)$	$\left(\left[\mathrm{0.2,0.7}\right],\left[\mathrm{0.3,0.4}\right]\right)$	$\left(\left[\mathrm{0.6,0.7}\right],\left[\mathrm{0.5,0.7}\right]\right)$	$\left(\left[\mathrm{0.4,0.9}\right],\left[\mathrm{0.3,0.4}\right]\right)$	$\left(\left[\mathrm{0.1,0.6}\right],\left[\mathrm{0.5,0.8}\right]\right)$	$\left(\left[\mathrm{0.1,0.8}\right],\left[\mathrm{0.4,0.5}\right]\right)$
$${\bar{\mathrm{U}}}_{5}$$						
${\boldsymbol{\ss }}_{1}$	$\left(\left[\mathrm{0.4,0.7}\right],\left[\mathrm{0.6,0.7}\right]\right)$	$\left(\left[\mathrm{0.3,0.6}\right],\left[\mathrm{0.6,0.7}\right]\right)$	$\left(\left[\mathrm{0.4,0.6}\right],\left[\mathrm{0.6,0.8}\right]\right)$	$\left(\left[\mathrm{0.4,0.5}\right],\left[\mathrm{0.2,0.8}\right]\right)$	$\left(\left[\mathrm{0.5,0.7}\right],\left[\mathrm{0.4,0.7}\right]\right)$	$\left(\left[\mathrm{0.5,0.8}\right],\left[\mathrm{0.3,0.6}\right]\right)$
${\boldsymbol{\ss }}_{2}$	$\left(\left[\mathrm{0.3,0.5}\right],\left[\mathrm{0.4,0.7}\right]\right)$	$\left(\left[\mathrm{0.2,0.5}\right],\left[\mathrm{0.6,0.8}\right]\right)$	$\left(\left[\mathrm{0.3,0.6}\right],\left[\mathrm{0.6,0.7}\right]\right)$	$\left(\left[\mathrm{0.1,0.4}\right],\left[\mathrm{0.4,0.5}\right]\right)$	$\left(\left[\mathrm{0.4,0.7}\right],\left[\mathrm{0.6,0.7}\right]\right)$	$\left(\left[\mathrm{0.3,0.5}\right],\left[\mathrm{0.5,0.7}\right]\right)$
${\boldsymbol{\ss }}_{3}$	$\left(\left[\mathrm{0.1,0.4}\right],\left[\mathrm{0.2,0.8}\right]\right)$	$\left(\left[\mathrm{0.4,0.6}\right],\left[\mathrm{0.1,0.5}\right]\right)$	$\left(\left[\mathrm{0.2,0.5}\right],\left[\mathrm{0.3,0.8}\right]\right)$	$\left(\left[\mathrm{0.3,0.6}\right],\left[\mathrm{0.2,0.7}\right]\right)$	$\left(\left[\mathrm{0.4,0.5}\right],\left[\mathrm{0.5,0.8}\right]\right)$	$\left(\left[\mathrm{0.5,07}\right],\left[\mathrm{0.4,0.5}\right]\right)$
${\boldsymbol{\ss }}_{4}$	$\left(\left[\mathrm{0.4,0.6}\right],\left[\mathrm{0.2,0.8}\right]\right)$	$\left(\left[\mathrm{0.3,0.4}\right],\left[\mathrm{0.5,0.7}\right]\right)$	$\left(\left[\mathrm{0.1,0.4}\right],\left[\mathrm{0.5,0.8}\right]\right)$	$\left(\left[\mathrm{0.4,0.6}\right],\left[\mathrm{0.3,0.5}\right]\right)$	$\left(\left[\mathrm{0.3,0.4}\right],\left[\mathrm{0.3,0.8}\right]\right)$	$\left(\left[\mathrm{0.2,0.6}\right],\left[\mathrm{0.5,0.8}\right]\right)$
${\boldsymbol{\ss }}_{5}$	$\left(\left[\mathrm{0.3,0.4}\right],\left[\mathrm{0.6,0.9}\right]\right)$	$\left(\left[\mathrm{0.4,0.6}\right],\left[\mathrm{0.4,0.7}\right]\right)$	$\left(\left[\mathrm{0.5,0.7}\right],\left[\mathrm{0.6,0.7}\right]\right)$	$\left(\left[\mathrm{0.3,0.5}\right],\left[\mathrm{0.6,0.8}\right]\right)$	$\left(\left[\mathrm{0.3,0.6}\right],\left[\mathrm{0.7,0.8}\right]\right)$	$\left(\left[0.5.0.5\right],\left[\mathrm{0.4,0.8}\right]\right)$

Step 2: Since we already know that, we may examine the matrix ${\left[{\Xi }_{{a}_{ij}}^{p}\right]}_{n\times m}$ by dividing the factors into two categories: those associated with costs and those associated with benefits. Normalizing the factors is unnecessary if they are of identical form. The other criteria that were taken into consideration are of a beneficial kind, whereas ${\mathfrak{R}}_{2}=\text{Worker health and safety}$, ${\mathfrak{R}}_{3}=\text{Financial ability}$ and ${\mathfrak{R}}_{6}=$ Waste reduction and recycling. Therefore, employing Eq. 7, which is provided in Table 3, to standardize the experts’ opinions is essential.

**Table 3:** Normalized decision matrices.

${\bar{\mathrm{U}}}_{1}$	${\mathfrak{R}}_{1}$	${\mathfrak{R}}_{2}$	${\mathfrak{R}}_{3}$	${\mathfrak{R}}_{4}$	${\mathfrak{R}}_{5}$	${\mathfrak{R}}_{6}$
${\boldsymbol{\ss }}_{1}$	$\left(\left[\mathrm{0.6,0.8}\right],\left[\mathrm{0.3,0.5}\right]\right)$	$\left(\left[\mathrm{0.4,0.6}\right],\left[\mathrm{0.3,0.8}\right]\right)$	$\left(\left[\mathrm{0.3,0.7}\right], \left[\mathrm{0.4,0.6}\right]\right)$	$\left(\left[\mathrm{0.1,0.8}\right],\left[\mathrm{0.4,0.6}\right]\right)$	$\left(\left[\mathrm{0.3,0.6}\right],\left[\mathrm{0.1,0.7}\right]\right)$	$\left(\left[\mathrm{0.6,0.8}\right], \left[\mathrm{0.4,0.5}\right]\right)$
${\boldsymbol{\ss }}_{2}$	$\left(\left[\mathrm{0.2,0.3}\right],\left[\mathrm{0.6,0.9}\right]\right)$	$\left(\left[\mathrm{0.2,0.8}\right], \left[\mathrm{0.4,0.5}\right]\right)$	$\left(\left[\mathrm{0.5,0.7}\right], \left[\mathrm{0.2,0.5}\right]\right)$	$\left(\left[\mathrm{0.3,0.6}\right],\left[\mathrm{0.4,0.8}\right]\right)$	$\left(\left[\mathrm{0.1,0.2}\right],\left[\mathrm{0.5,0.8}\right]\right)$	$\left(\left[\mathrm{0.5,0.7}\right], \left[\mathrm{0.6,0.6}\right]\right)$
${\boldsymbol{\ss }}_{3}$	$\left(\left[\mathrm{0.6,0.7}\right],\left[\mathrm{0.2,0.7}\right]\right)$	$\left(\left[\mathrm{0.4,0.7}\right],\left[\mathrm{0.5,0.7}\right]\right)$	$\left(\left[\mathrm{0.1,0.3}\right], \left[\mathrm{0.4,0.5}\right]\right)$	$\left(\left[\mathrm{0.4,0.7}\right],\left[\mathrm{0.3,0.5}\right]\right)$	$\left(\left[\mathrm{0.7,0.8}\right],\left[\mathrm{0.4,0.6}\right]\right)$	$\left(\left[\mathrm{0.3,0.5}\right], \left[\mathrm{0.6,0.8}\right]\right)$
${\boldsymbol{\ss }}_{4}$	$\left(\left[\mathrm{0.1,0.4}\right],\left[\mathrm{0.4,0.6}\right]\right)$	$\left(\left[\mathrm{0.1,0.7}\right], \left[\mathrm{0.3,0.6}\right]\right)$	$\left(\left[\mathrm{0.3,0.7}\right], \left[\mathrm{0.6,0.6}\right]\right)$	$\left(\left[\mathrm{0.2,0.3}\right],\left[\mathrm{0.1,0.8}\right]\right)$	$\left(\left[\mathrm{0.4,0.6}\right],\left[\mathrm{0.5,0.7}\right]\right)$	$\left(\left[\mathrm{0.5,0.7}\right], \left[\mathrm{0.2,0.5}\right]\right)$
${\boldsymbol{\ss }}_{5}$	$\left(\left[\mathrm{0.7,0.8}\right],\left[\mathrm{0.3,0.4}\right]\right)$	$\left(\left[\mathrm{0.4,0.6}\right], \left[\mathrm{0.3,0.7}\right]\right)$	$\left(\left[\mathrm{0.2,0.6}\right], \left[\mathrm{0.4,0.8}\right]\right)$	$\left(\left[\mathrm{0.3,0.8}\right],\left[\mathrm{0.5,0.6}\right]\right)$	$\left(\left[\mathrm{0.1,0.8}\right],\left[\mathrm{0.4,0.5}\right]\right)$	$\left(\left[\mathrm{0.6,0.8}\right], \left[\mathrm{0.2,0.3}\right]\right)$
$${{\bar{\mathrm{U}}}}_{2}$$						
${\boldsymbol{\ss }}_{1}$	$\left(\left[\mathrm{0.4,0.7}\right],\left[\mathrm{0.2,0.6}\right]\right)$	$\left(\left[\mathrm{0.4,0.5}\right], \left[\mathrm{0.4,0.7}\right]\right)$	$\left(\left[\mathrm{0.6,0.8}\right], \left[\mathrm{0.4,0.5}\right]\right)$	$\left(\left[\mathrm{0.4,0.7}\right],\left[\mathrm{0.1,0.6}\right]\right)$	$\left(\left[\mathrm{0.6,0.7}\right],\left[\mathrm{0.2,0.6}\right]\right)$	$\left(\left[\mathrm{0.3,0.6}\right], \left[\mathrm{0.5,0.7}\right]\right)$
${\boldsymbol{\ss }}_{2}$	$\left(\left[\mathrm{0.6,0.8}\right],\left[\mathrm{0.3,0.4}\right]\right)$	$\left(\left[\mathrm{0.5,0.8}\right], \left[\mathrm{0.2,0.6}\right]\right)$	$\left(\left[\mathrm{0.5,0.7}\right], \left[\mathrm{0.6,0.6}\right]\right)$	$\left(\left[\mathrm{0.1,0.5}\right],\left[\mathrm{0.1,0.8}\right]\right)$	$\left(\left[\mathrm{0.4,0.7}\right],\left[\mathrm{0.6,0.6}\right]\right)$	$\left(\left[\mathrm{0.3,0.7}\right], \left[\mathrm{0.4,0.5}\right]\right)$
${\boldsymbol{\ss }}_{3}$	$\left(\left[\mathrm{0.1,0.8}\right],\left[\mathrm{0.1,0.5}\right]\right)$	$\left(\left[\mathrm{0.3,0.4}\right], \left[\mathrm{0.3,0.8}\right]\right)$	$\left(\left[\mathrm{0.3,0.5}\right], \left[\mathrm{0.6,0.8}\right]\right)$	$\left(\left[\mathrm{0.3,0.4}\right],\left[\mathrm{0.3,0.4}\right]\right)$	$\left(\left[\mathrm{0.3,0.5}\right],\left[\mathrm{0.4,0.8}\right]\right)$	$\left(\left[\mathrm{0.3,0.5}\right], \left[\mathrm{0.2,0.6}\right]\right)$
${\boldsymbol{\ss }}_{4}$	$\left(\left[\mathrm{0.3,0.8}\right],\left[\mathrm{0.4,0.6}\right]\right)$	$\left(\left[\mathrm{0.4,0.6}\right], \left[\mathrm{0.4,0.8}\right]\right)$	$\left(\left[\mathrm{0.5,0.7}\right], \left[\mathrm{0.2,0.5}\right]\right)$	$\left(\left[\mathrm{0.2,0.4}\right],\left[\mathrm{0.5,0.8}\right]\right)$	$\left(\left[\mathrm{0.3,0.6}\right],\left[\mathrm{0.5,0.7}\right]\right)$	$\left(\left[\mathrm{0.4,0.6}\right], \left[\mathrm{0.3,0.4}\right]\right)$
${\boldsymbol{\ss }}_{5}$	$\left(\left[\mathrm{0.3,0.4}\right],\left[\mathrm{0.3,0.5}\right]\right)$	$\left(\left[\mathrm{0.1,0.2}\right], \left[\mathrm{0.3,0.9}\right]\right)$	$\left(\left[\mathrm{0.6,0.8}\right], \left[\mathrm{0.2,0.3}\right]\right)$	$\left(\left[\mathrm{0.1,0.4}\right],\left[\mathrm{0.5,0.8}\right]\right)$	$\left(\left[\mathrm{0.4,0.8}\right],\left[\mathrm{0.4,0.6}\right]\right)$	$\left(\left[\mathrm{0.3,0.5}\right], \left[\mathrm{0.5,0.8}\right]\right)$
$${{\bar{\mathrm{U}}}}_{3}$$						
${\boldsymbol{\ss }}_{1}$	$\left(\left[\mathrm{0.5,0.7}\right],\left[\mathrm{0.3,0.6}\right]\right)$	$\left(\left[\mathrm{0.2,0.6}\right], \left[\mathrm{0.4,0.5}\right]\right)$	$\left(\left[\mathrm{0.3,0.4}\right], \left[\mathrm{0.5,0.9}\right]\right)$	$\left(\left[\mathrm{0.4,0.6}\right],\left[\mathrm{0.3,0.4}\right]\right)$	$\left(\left[\mathrm{0.2,0.5}\right],\left[\mathrm{0.5,0.8}\right]\right)$	$\left(\left[\mathrm{0.4,0.6}\right], \left[\mathrm{0.3,0.8}\right]\right)$
${\boldsymbol{\ss }}_{2}$	$\left(\left[\mathrm{0.4,0.5}\right],\left[\mathrm{0.3,0.7}\right]\right)$	$\left(\left[\mathrm{0.4,0.6}\right], \left[\mathrm{0.5,0.5}\right]\right)$	$\left(\left[\mathrm{0.4,0.6}\right], \left[\mathrm{0.3,0.5}\right]\right)$	$\left(\left[\mathrm{0.1,0.6}\right],\left[\mathrm{0.3,0.8}\right]\right)$	$\left(\left[\mathrm{0.4,0.6}\right],\left[\mathrm{0.6,0.8}\right]\right)$	$\left(\left[\mathrm{0.2,0.8}\right], \left[\mathrm{0.4,0.5}\right]\right)$
${\boldsymbol{\ss }}_{3}$	$\left(\left[\mathrm{0.2,0.6}\right],\left[\mathrm{0.3,0.5}\right]\right)$	$\left(\left[\mathrm{0.3,0.7}\right], \left[\mathrm{0.5,0.6}\right]\right)$	$\left(\left[\mathrm{0.2,0.4}\right], \left[\mathrm{0.3,0.8}\right]\right)$	$\left(\left[\mathrm{0.5,0.6}\right],\left[\mathrm{0.1,0.7}\right]\right)$	$\left(\left[\mathrm{0.5,0.7}\right],\left[\mathrm{0.3,0.6}\right]\right)$	$\left(\left[\mathrm{0.4,0.7}\right], \left[\mathrm{0.5,0.7}\right]\right)$
${\boldsymbol{\ss }}_{4}$	$\left(\left[\mathrm{0.3,0.4}\right],\left[\mathrm{0.4,0.6}\right]\right)$	$\left(\left[\mathrm{0.2,0.8}\right], \left[\mathrm{0.2,0.5}\right]\right)$	$\left(\left[\mathrm{0.4,0.8}\right], \left[\mathrm{0.3,0.6}\right]\right)$	$\left(\left[\mathrm{0.4,0.7}\right],\left[\mathrm{0.6,0.7}\right]\right)$	$\left(\left[\mathrm{0.4,0.6}\right],\left[\mathrm{0.1,0.5}\right]\right)$	$\left(\left[\mathrm{0.1,0.7}\right], \left[\mathrm{0.3,0.6}\right]\right)$
${\boldsymbol{\ss }}_{5}$	$\left(\left[\mathrm{0.5,0.8}\right],\left[\mathrm{0.3,0.5}\right]\right)$	$\left(\left[\mathrm{0.3,0.4}\right], \left[\mathrm{0.6,0.9}\right]\right)$	$\left(\left[\mathrm{0.1,0.4}\right], \left[\mathrm{0.8,0.9}\right]\right)$	$\left(\left[\mathrm{0.3,0.4}\right],\left[\mathrm{0.3,0.4}\right]\right)$	$\left(\left[\mathrm{0.4,0.7}\right],\left[\mathrm{0.3,0.6}\right]\right)$	$\left(\left[\mathrm{0.4,0.6}\right], \left[\mathrm{0.3,0.7}\right]\right)$
$${{\bar{\mathrm{U}}}}_{4}$$						
${\boldsymbol{\ss }}_{1}$	$\left(\left[\mathrm{0.5,0.8}\right],\left[\mathrm{0.3,0.6}\right]\right)$	$\left(\left[\mathrm{0.2,0.4}\right], \left[\mathrm{0.5,0.6}\right]\right)$	$\left(\left[\mathrm{0.5,0.7}\right], \left[\mathrm{0.4,0.6}\right]\right)$	$\left(\left[\mathrm{0.6,0.8}\right],\left[\mathrm{0.2,0.5}\right]\right)$	$\left(\left[\mathrm{0.5,0.7}\right],\left[\mathrm{0.4,0.6}\right]\right)$	$\left(\left[\mathrm{0.1,0.7}\right], \left[\mathrm{0.3,0.6}\right]\right)$
${\boldsymbol{\ss }}_{2}$	$\left(\left[\mathrm{0.3,0.5}\right],\left[\mathrm{0.5,0.7}\right]\right)$	$\left(\left[\mathrm{0.5,0.7}\right], \left[\mathrm{0.5,0.7}\right]\right)$	$\left(\left[\mathrm{0.3,0.5}\right], \left[\mathrm{0.6,0.8}\right]\right)$	$\left(\left[\mathrm{0.1,0.7}\right],\left[\mathrm{0.5,0.7}\right]\right)$	$\left(\left[\mathrm{0.3,0.5}\right],\left[\mathrm{0.4,0.5}\right]\right)$	$\left(\left[\mathrm{0.5,0.8}\right], \left[\mathrm{0.1,0.2}\right]\right)$
${\boldsymbol{\ss }}_{3}$	$\left(\left[\mathrm{0.5,07}\right],\left[\mathrm{0.4,0.5}\right]\right)$	$\left(\left[\mathrm{0.1,0.6}\right], \left[\mathrm{0.2,0.8}\right]\right)$	$\left(\left[\mathrm{0.5,0.6}\right], \left[\mathrm{0.5,0.8}`\right]\right)$	$\left(\left[\mathrm{0.4,0.8}\right],\left[\mathrm{0.6,0.6}\right]\right)$	$\left(\left[\mathrm{0.1,0.4}\right],\left[\mathrm{0.7,0.9}\right]\right)$	$\left(\left[\mathrm{0.4,0.6}\right], \left[\mathrm{0.7,0.8}\right]\right)$
${\boldsymbol{\ss }}_{4}$	$\left(\left[\mathrm{0.2,0.6}\right],\left[\mathrm{0.5,0.8}\right]\right)$	$\left(\left[\mathrm{0.6,0.7}\right], \left[\mathrm{0.2,0.4}\right]\right)$	$\left(\left[\mathrm{0.6,0.8}\right], \left[\mathrm{0.1,0.6}\right]\right)$	$\left(\left[\mathrm{0.3,0.7}\right],\left[\mathrm{0.5,0.6}\right]\right)$	$\left(\left[\mathrm{0.5,0.8}\right],\left[\mathrm{0.4,0.5}\right]\right)$	$\left(\left[\mathrm{0.5,0.7}\right], \left[\mathrm{0.4,0.6}\right]\right)$
${\boldsymbol{\ss }}_{5}$	$\left(\left[0.5.0.5\right],\left[\mathrm{0.4,0.8}\right]\right)$	$\left(\left[\mathrm{0.3,0.4}\right], \left[\mathrm{0.2,0.7}\right]\right)$	$\left(\left[\mathrm{0.5,0.7}\right], \left[\mathrm{0.6,0.7}\right]\right)$	$\left(\left[\mathrm{0.4,0.9}\right],\left[\mathrm{0.3,0.4}\right]\right)$	$\left(\left[\mathrm{0.1,0.6}\right],\left[\mathrm{0.5,0.8}\right]\right)$	$\left(\left[\mathrm{0.4,0.5}\right], \left[\mathrm{0.1,0.8}\right]\right)$
$${{\bar{\mathrm{U}}}}_{5}$$						
${\boldsymbol{\ss }}_{1}$	$\left(\left[\mathrm{0.4,0.7}\right],\left[\mathrm{0.6,0.7}\right]\right)$	$\left(\left[\mathrm{0.6,0.7}\right], \left[\mathrm{0.3,0.6}\right]\right)$	$\left(\left[\mathrm{0.6,0.8}\right], \left[\mathrm{0.4,0.6}\right]\right)$	$\left(\left[\mathrm{0.4,0.5}\right],\left[\mathrm{0.2,0.8}\right]\right)$	$\left(\left[\mathrm{0.5,0.7}\right],\left[\mathrm{0.4,0.7}\right]\right)$	$\left(\left[\mathrm{0.3,0.6}\right], \left[\mathrm{0.5,0.8}\right]\right)$
${\boldsymbol{\ss }}_{2}$	$\left(\left[\mathrm{0.3,0.5}\right],\left[\mathrm{0.4,0.7}\right]\right)$	$\left(\left[\mathrm{0.6,0.8}\right], \left[\mathrm{0.2,0.5}\right]\right)$	$\left(\left[\mathrm{0.6,0.7}\right], \left[\mathrm{0.3,0.6}\right]\right)$	$\left(\left[\mathrm{0.1,0.4}\right],\left[\mathrm{0.4,0.5}\right]\right)$	$\left(\left[\mathrm{0.4,0.7}\right],\left[\mathrm{0.6,0.7}\right]\right)$	$\left(\left[\mathrm{0.5,0.7}\right], \left[\mathrm{0.3,0.5}\right]\right)$
${\boldsymbol{\ss }}_{3}$	$\left(\left[\mathrm{0.1,0.4}\right],\left[\mathrm{0.2,0.8}\right]\right)$	$\left(\left[\mathrm{0.1,0.5}\right], \left[\mathrm{0.4,0.6}\right]\right)$	$\left(\left[\mathrm{0.3,0.8}\right], \left[\mathrm{0.2,0.5}\right]\right)$	$\left(\left[\mathrm{0.3,0.6}\right],\left[\mathrm{0.2,0.7}\right]\right)$	$\left(\left[\mathrm{0.4,0.5}\right],\left[\mathrm{0.5,0.8}\right]\right)$	$\left(\left[\mathrm{0.4,0.5}\right], \left[\mathrm{0.5,07}\right]\right)$
${\boldsymbol{\ss }}_{4}$	$\left(\left[\mathrm{0.4,0.6}\right],\left[\mathrm{0.2,0.8}\right]\right)$	$\left(\left[\mathrm{0.5,0.7}\right], \left[\mathrm{0.3,0.4}\right]\right)$	$\left(\left[\mathrm{0.5,0.8}\right], \left[\mathrm{0.1,0.4}\right]\right)$	$\left(\left[\mathrm{0.4,0.6}\right],\left[\mathrm{0.3,0.5}\right]\right)$	$\left(\left[\mathrm{0.3,0.4}\right],\left[\mathrm{0.3,0.8}\right]\right)$	$\left(\left[\mathrm{0.5,0.8}\right], \left[\mathrm{0.2,0.6}\right]\right)$
${\boldsymbol{\ss }}_{5}$	$\left(\left[\mathrm{0.3,0.4}\right],\left[\mathrm{0.6,0.9}\right]\right)$	$\left(\left[\mathrm{0.4,0.7}\right], \left[\mathrm{0.4,0.6}\right]\right)$	$\left(\left[\mathrm{0.6,0.7}\right], \left[\mathrm{0.5,0.7}\right]\right)$	$\left(\left[\mathrm{0.3,0.5}\right],\left[\mathrm{0.6,0.8}\right]\right)$	$\left(\left[\mathrm{0.3,0.6}\right],\left[\mathrm{0.7,0.8}\right]\right)$	$\left(\left[\mathrm{0.4,0.8}\right], \left[0.5.0.5\right]\right)$

**Step 3:** Look at Table 4 to view the ordered decision matrices.

**Table 4:** Ordered decision matrices.

${{\bar{\mathrm{U}}}}_{1}$	${\mathfrak{R}}_{1}$	${\mathfrak{R}}_{2}$	${\mathfrak{R}}_{3}$	${\mathfrak{R}}_{4}$	${\mathfrak{R}}_{5}$	${\mathfrak{R}}_{6}$
${\boldsymbol{\ss }}_{1}$	$\left(\left[\mathrm{0.7,0.8}\right],\left[\mathrm{0.3,0.4}\right]\right)$	$\left(\left[\mathrm{0.6,0.8}\right], \left[\mathrm{0.4,0.5}\right]\right)$	$\left(\left[\mathrm{0.1,0.8}\right],\left[\mathrm{0.4,0.6}\right]\right)$	$\left(\left[\mathrm{0.3,0.6}\right],\left[\mathrm{0.1,0.7}\right]\right)$	$\left(\left[\mathrm{0.4,0.6}\right],\left[\mathrm{0.3,0.8}\right]\right)$	$\left(\left[\mathrm{0.3,0.5}\right], \left[\mathrm{0.6,0.8}\right]\right)$
${\boldsymbol{\ss }}_{2}$	$\left(\left[\mathrm{0.6,0.8}\right],\left[\mathrm{0.3,0.5}\right]\right)$	$\left(\left[\mathrm{0.5,0.7}\right], \left[\mathrm{0.2,0.5}\right]\right)$	$\left(\left[\mathrm{0.3,0.7}\right], \left[\mathrm{0.4,0.6}\right]\right)$	$\left(\left[\mathrm{0.5,0.7}\right], \left[\mathrm{0.6,0.6}\right]\right)$	$\left(\left[\mathrm{0.4,0.6}\right],\left[\mathrm{0.5,0.7}\right]\right)$	$\left(\left[\mathrm{0.3,0.6}\right],\left[\mathrm{0.4,0.8}\right]\right)$
${\boldsymbol{\ss }}_{3}$	$\left(\left[\mathrm{0.7,0.8}\right],\left[\mathrm{0.4,0.6}\right]\right)$	$\left(\left[\mathrm{0.6,0.7}\right],\left[\mathrm{0.2,0.7}\right]\right)$	$\left(\left[\mathrm{0.4,0.7}\right],\left[\mathrm{0.3,0.5}\right]\right)$	$\left(\left[\mathrm{0.4,0.7}\right],\left[\mathrm{0.5,0.7}\right]\right)$	$\left(\left[\mathrm{0.1,0.3}\right], \left[\mathrm{0.4,0.5}\right]\right)$	$\left(\left[\mathrm{0.2,0.3}\right],\left[\mathrm{0.6,0.9}\right]\right)$
${\boldsymbol{\ss }}_{4}$	$\left(\left[\mathrm{0.5,0.7}\right], \left[\mathrm{0.2,0.5}\right]\right)$	$\left(\left[\mathrm{0.2,0.8}\right], \left[\mathrm{0.4,0.5}\right]\right)$	$\left(\left[\mathrm{0.1,0.7}\right], \left[\mathrm{0.3,0.6}\right]\right)$	$\left(\left[\mathrm{0.3,0.7}\right], \left[\mathrm{0.6,0.6}\right]\right)$	$\left(\left[\mathrm{0.1,0.4}\right],\left[\mathrm{0.4,0.6}\right]\right)$	$\left(\left[\mathrm{0.2,0.3}\right],\left[\mathrm{0.1,0.8}\right]\right)$
${\boldsymbol{\ss }}_{5}$	$\left(\left[\mathrm{0.6,0.8}\right], \left[\mathrm{0.2,0.3}\right]\right)$	$\left(\left[\mathrm{0.1,0.8}\right],\left[\mathrm{0.4,0.5}\right]\right)$	$\left(\left[\mathrm{0.3,0.8}\right],\left[\mathrm{0.5,0.6}\right]\right)$	$\left(\left[\mathrm{0.4,0.6}\right], \left[\mathrm{0.3,0.7}\right]\right)$	$\left(\left[\mathrm{0.2,0.6}\right], \left[\mathrm{0.4,0.8}\right]\right)$	$\left(\left[\mathrm{0.1,0.2}\right],\left[\mathrm{0.5,0.8}\right]\right)$
$${{\bar{\mathrm{U}}}}_{2}$$						
${\boldsymbol{\ss }}_{1}$	$\left(\left[\mathrm{0.6,0.8}\right],\left[\mathrm{0.3,0.4}\right]\right)$	$\left(\left[\mathrm{0.6,0.8}\right], \left[\mathrm{0.4,0.5}\right]\right)$	$\left(\left[\mathrm{0.6,0.7}\right],\left[\mathrm{0.2,0.6}\right]\right)$	$\left(\left[\mathrm{0.4,0.7}\right],\left[\mathrm{0.1,0.6}\right]\right)$	$\left(\left[\mathrm{0.4,0.7}\right],\left[\mathrm{0.2,0.6}\right]\right)$	$\left(\left[\mathrm{0.4,0.5}\right], \left[\mathrm{0.4,0.7}\right]\right)$
${\boldsymbol{\ss }}_{2}$	$\left(\left[\mathrm{0.5,0.8}\right], \left[\mathrm{0.2,0.6}\right]\right)$	$\left(\left[\mathrm{0.3,0.7}\right], \left[\mathrm{0.4,0.5}\right]\right)$	$\left(\left[\mathrm{0.5,0.7}\right], \left[\mathrm{0.6,0.6}\right]\right)$	$\left(\left[\mathrm{0.4,0.7}\right],\left[\mathrm{0.6,0.6}\right]\right)$	$\left(\left[\mathrm{0.3,0.6}\right],\left[\mathrm{0.5,0.7}\right]\right)$	$\left(\left[\mathrm{0.3,0.6}\right], \left[\mathrm{0.5,0.7}\right]\right)$
${\boldsymbol{\ss }}_{3}$	$\left(\left[\mathrm{0.1,0.8}\right],\left[\mathrm{0.1,0.5}\right]\right)$	$\left(\left[\mathrm{0.3,0.4}\right],\left[\mathrm{0.3,0.4}\right]\right)$	$\left(\left[\mathrm{0.3,0.5}\right], \left[\mathrm{0.2,0.6}\right]\right)$	$\left(\left[\mathrm{0.1,0.5}\right],\left[\mathrm{0.1,0.8}\right]\right)$	$\left(\left[\mathrm{0.3,0.5}\right],\left[\mathrm{0.4,0.8}\right]\right)$	$\left(\left[\mathrm{0.3,0.4}\right], \left[\mathrm{0.3,0.8}\right]\right)$
${\boldsymbol{\ss }}_{4}$	$\left(\left[\mathrm{0.5,0.7}\right], \left[\mathrm{0.2,0.5}\right]\right)$	$\left(\left[\mathrm{0.4,0.6}\right], \left[\mathrm{0.3,0.4}\right]\right)$	$\left(\left[\mathrm{0.3,0.8}\right],\left[\mathrm{0.4,0.6}\right]\right)$	$\left(\left[\mathrm{0.4,0.6}\right], \left[\mathrm{0.4,0.8}\right]\right)$	$\left(\left[\mathrm{0.2,0.4}\right],\left[\mathrm{0.5,0.8}\right]\right)$	$\left(\left[\mathrm{0.3,0.5}\right], \left[\mathrm{0.6,0.8}\right]\right)$
${\boldsymbol{\ss }}_{5}$	$\left(\left[\mathrm{0.6,0.8}\right], \left[\mathrm{0.2,0.3}\right]\right)$	$\left(\left[\mathrm{0.4,0.8}\right],\left[\mathrm{0.4,0.6}\right]\right)$	$\left(\left[\mathrm{0.3,0.4}\right],\left[\mathrm{0.3,0.5}\right]\right)$	$\left(\left[\mathrm{0.3,0.5}\right], \left[\mathrm{0.5,0.8}\right]\right)$	$\left(\left[\mathrm{0.1,0.4}\right],\left[\mathrm{0.5,0.8}\right]\right)$	$\left(\left[\mathrm{0.1,0.2}\right], \left[\mathrm{0.3,0.9}\right]\right)$
$${{\bar{\mathrm{U}}}}_{3}$$						
${\boldsymbol{\ss }}_{1}$	$\left(\left[\mathrm{0.5,0.8}\right],\left[\mathrm{0.3,0.5}\right]\right)$	$\left(\left[\mathrm{0.4,0.6}\right],\left[\mathrm{0.3,0.4}\right]\right)$	$\left(\left[\mathrm{0.2,0.6}\right], \left[\mathrm{0.4,0.5}\right]\right)$	$\left(\left[\mathrm{0.4,0.7}\right],\left[\mathrm{0.6,0.7}\right]\right)$	$\left(\left[\mathrm{0.4,0.6}\right], \left[\mathrm{0.3,0.8}\right]\right)$	$\left(\left[\mathrm{0.2,0.5}\right],\left[\mathrm{0.5,0.8}\right]\right)$
${\boldsymbol{\ss }}_{2}$	$\left(\left[\mathrm{0.2,0.8}\right], \left[\mathrm{0.4,0.5}\right]\right)$	$\left(\left[\mathrm{0.4,0.6}\right], \left[\mathrm{0.3,0.5}\right]\right)$	$\left(\left[\mathrm{0.4,0.5}\right],\left[\mathrm{0.3,0.7}\right]\right)$	$\left(\left[\mathrm{0.1,0.6}\right],\left[\mathrm{0.3,0.8}\right]\right)$	$\left(\left[\mathrm{0.4,0.6}\right],\left[\mathrm{0.6,0.8}\right]\right)$	$\left(\left[\mathrm{0.4,0.7}\right], \left[\mathrm{0.5,0.7}\right]\right)$
${\boldsymbol{\ss }}_{3}$	$\left(\left[\mathrm{0.5,0.7}\right],\left[\mathrm{0.3,0.6}\right]\right)$	$\left(\left[\mathrm{0.5,0.7}\right],\left[\mathrm{0.3,0.6}\right]\right)$	$\left(\left[\mathrm{0.5,0.6}\right],\left[\mathrm{0.1,0.7}\right]\right)$	$\left(\left[\mathrm{0.2,0.6}\right],\left[\mathrm{0.3,0.5}\right]\right)$	$\left(\left[\mathrm{0.3,0.7}\right], \left[\mathrm{0.5,0.6}\right]\right)$	$\left(\left[\mathrm{0.2,0.4}\right], \left[\mathrm{0.3,0.8}\right]\right)$
${\boldsymbol{\ss }}_{4}$	$\left(\left[\mathrm{0.2,0.8}\right], \left[\mathrm{0.2,0.5}\right]\right)$	$\left(\left[\mathrm{0.4,0.8}\right], \left[\mathrm{0.3,0.6}\right]\right)$	$\left(\left[\mathrm{0.4,0.6}\right],\left[\mathrm{0.1,0.5}\right]\right)$	$\left(\left[\mathrm{0.1,0.7}\right], \left[\mathrm{0.3,0.6}\right]\right)$	$\left(\left[\mathrm{0.3,0.4}\right],\left[\mathrm{0.4,0.6}\right]\right)$	$\left(\left[\mathrm{0.3,0.4}\right], \left[\mathrm{0.5,0.9}\right]\right)$
${\boldsymbol{\ss }}_{5}$	$\left(\left[\mathrm{0.4,0.7}\right],\left[\mathrm{0.3,0.6}\right]\right)$	$\left(\left[\mathrm{0.4,0.6}\right], \left[\mathrm{0.5,0.5}\right]\right)$	$\left(\left[\mathrm{0.3,0.4}\right],\left[\mathrm{0.3,0.4}\right]\right)$	$\left(\left[\mathrm{0.4,0.6}\right], \left[\mathrm{0.3,0.7}\right]\right)$	$\left(\left[\mathrm{0.3,0.4}\right], \left[\mathrm{0.6,0.9}\right]\right)$	$\left(\left[\mathrm{0.1,0.4}\right], \left[\mathrm{0.8,0.9}\right]\right)$
$${{\bar{\mathrm{U}}}}_{4}$$						
${\boldsymbol{\ss }}_{1}$	$\left(\left[\mathrm{0.6,0.8}\right],\left[\mathrm{0.2,0.5}\right]\right)$	$\left(\left[\mathrm{0.6,0.7}\right], \left[\mathrm{0.2,0.4}\right]\right)$	$\left(\left[\mathrm{0.5,0.7}\right], \left[\mathrm{0.4,0.6}\right]\right)$	$\left(\left[\mathrm{0.5,0.7}\right],\left[\mathrm{0.4,0.6}\right]\right)$	$\left(\left[\mathrm{0.1,0.7}\right], \left[\mathrm{0.3,0.6}\right]\right)$	$\left(\left[\mathrm{0.2,0.4}\right], \left[\mathrm{0.5,0.6}\right]\right)$
${\boldsymbol{\ss }}_{2}$	$\left(\left[\mathrm{0.5,0.8}\right], \left[\mathrm{0.1,0.2}\right]\right)$	$\left(\left[\mathrm{0.5,0.7}\right], \left[\mathrm{0.5,0.7}\right]\right)$	$\left(\left[\mathrm{0.3,0.5}\right],\left[\mathrm{0.4,0.5}\right]\right)$	$\left(\left[\mathrm{0.3,0.7}\right],\left[\mathrm{0.5,0.6}\right]\right)$	$\left(\left[\mathrm{0.3,0.5}\right],\left[\mathrm{0.5,0.7}\right]\right)$	$\left(\left[\mathrm{0.4,0.6}\right], \left[\mathrm{0.7,0.8}\right]\right)$
${\boldsymbol{\ss }}_{3}$	$\left(\left[\mathrm{0.5,0.8}\right],\left[\mathrm{0.3,0.6}\right]\right)$	$\left(\left[\mathrm{0.5,07}\right],\left[\mathrm{0.4,0.5}\right]\right)$	$\left(\left[\mathrm{0.4,0.8}\right],\left[\mathrm{0.6,0.6}\right]\right)$	$\left(\left[\mathrm{0.1,0.7}\right],\left[\mathrm{0.5,0.7}\right]\right)$	$\left(\left[\mathrm{0.1,0.6}\right], \left[\mathrm{0.2,0.8}\right]\right)$	$\left(\left[\mathrm{0.5,0.6}\right], \left[\mathrm{0.5,0.8}`\right]\right)$
${\boldsymbol{\ss }}_{4}$	$\left(\left[\mathrm{0.6,0.8}\right], \left[\mathrm{0.1,0.6}\right]\right)$	$\left(\left[\mathrm{0.5,0.8}\right],\left[\mathrm{0.4,0.5}\right]\right)$	$\left(\left[\mathrm{0.5,0.7}\right], \left[\mathrm{0.4,0.6}\right]\right)$	$\left(\left[\mathrm{0.2,0.6}\right],\left[\mathrm{0.5,0.8}\right]\right)$	$\left(\left[\mathrm{0.1,0.6}\right],\left[\mathrm{0.5,0.8}\right]\right)$	$\left(\left[\mathrm{0.1,0.4}\right],\left[\mathrm{0.7,0.9}\right]\right)$
${\boldsymbol{\ss }}_{5}$	$\left(\left[\mathrm{0.4,0.9}\right],\left[\mathrm{0.3,0.4}\right]\right)$	$\left(\left[\mathrm{0.5,0.7}\right], \left[\mathrm{0.6,0.7}\right]\right)$	$\left(\left[\mathrm{0.4,0.5}\right], \left[\mathrm{0.1,0.8}\right]\right)$	$\left(\left[\mathrm{0.3,0.4}\right], \left[\mathrm{0.2,0.7}\right]\right)$	$\left(\left[0.5.0.5\right],\left[\mathrm{0.4,0.8}\right]\right)$	$\left(\left[\mathrm{0.3,0.5}\right], \left[\mathrm{0.6,0.8}\right]\right)$
$${{\bar{\mathrm{U}}}}_{5}$$						
${\boldsymbol{\ss }}_{1}$	$\left(\left[\mathrm{0.6,0.8}\right], \left[\mathrm{0.4,0.6}\right]\right)$	$\left(\left[\mathrm{0.5,0.7}\right], \left[\mathrm{0.3,0.4}\right]\right)$	$\left(\left[\mathrm{0.6,0.7}\right], \left[\mathrm{0.3,0.6}\right]\right)$	$\left(\left[\mathrm{0.5,0.7}\right],\left[\mathrm{0.4,0.7}\right]\right)$	$\left(\left[\mathrm{0.4,0.7}\right],\left[\mathrm{0.6,0.7}\right]\right)$	$\left(\left[\mathrm{0.4,0.5}\right], \left[\mathrm{0.5,07}\right]\right)$
${\boldsymbol{\ss }}_{2}$	$\left(\left[\mathrm{0.6,0.8}\right], \left[\mathrm{0.2,0.5}\right]\right)$	$\left(\left[\mathrm{0.6,0.7}\right], \left[\mathrm{0.3,0.6}\right]\right)$	$\left(\left[\mathrm{0.5,0.7}\right], \left[\mathrm{0.3,0.5}\right]\right)$	$\left(\left[\mathrm{0.4,0.7}\right],\left[\mathrm{0.6,0.7}\right]\right)$	$\left(\left[\mathrm{0.1,0.4}\right],\left[\mathrm{0.2,0.8}\right]\right)$	$\left(\left[\mathrm{0.3,0.6}\right], \left[\mathrm{0.5,0.8}\right]\right)$
${\boldsymbol{\ss }}_{3}$	$\left(\left[\mathrm{0.3,0.8}\right], \left[\mathrm{0.2,0.5}\right]\right)$	$\left(\left[\mathrm{0.4,0.7}\right], \left[\mathrm{0.4,0.6}\right]\right)$	$\left(\left[\mathrm{0.3,0.6}\right],\left[\mathrm{0.2,0.7}\right]\right)$	$\left(\left[\mathrm{0.1,0.4}\right],\left[\mathrm{0.4,0.5}\right]\right)$	$\left(\left[\mathrm{0.1,0.5}\right], \left[\mathrm{0.4,0.6}\right]\right)$	$\left(\left[\mathrm{0.3,0.5}\right],\left[\mathrm{0.4,0.7}\right]\right)$
${\boldsymbol{\ss }}_{4}$	$\left(\left[\mathrm{0.5,0.8}\right], \left[\mathrm{0.1,0.4}\right]\right)$	$\left(\left[\mathrm{0.5,0.8}\right], \left[\mathrm{0.2,0.6}\right]\right)$	$\left(\left[\mathrm{0.4,0.6}\right],\left[\mathrm{0.3,0.5}\right]\right)$	$\left(\left[\mathrm{0.4,0.6}\right],\left[\mathrm{0.2,0.8}\right]\right)$	$\left(\left[\mathrm{0.3,0.4}\right],\left[\mathrm{0.3,0.8}\right]\right)$	$\left(\left[\mathrm{0.3,0.4}\right],\left[\mathrm{0.6,0.9}\right]\right)$
${\boldsymbol{\ss }}_{5}$	$\left(\left[\mathrm{0.4,0.8}\right], \left[0.5.0.5\right]\right)$	$\left(\left[\mathrm{0.6,0.7}\right], \left[\mathrm{0.5,0.7}\right]\right)$	$\left(\left[\mathrm{0.3,0.5}\right],\left[\mathrm{0.6,0.8}\right]\right)$	$\left(\left[\mathrm{0.4,0.5}\right],\left[\mathrm{0.2,0.8}\right]\right)$	$\left(\left[\mathrm{0.4,0.5}\right],\left[\mathrm{0.5,0.8}\right]\right)$	$\left(\left[\mathrm{0.3,0.6}\right],\left[\mathrm{0.7,0.8}\right]\right)$

After the individual assessment, the experts’ findings are stated as IVPFSNs, which can be evaluated using the proposed Einstein-based AOs to build the aggregated decision matrix for the EDAS technique. This procedure endorsed the final assessment by replicating the combined data of the full expert panel while stabilizing the hesitation and reluctance built into human decision-making.

Step 4: Table 5 demonstrates the compilation of the aggregated decision matrix using the IVPFSEOWA operator.

**Table 5:** Aggregated decision matrix for IVPFSEOWA operator.

	${\mathfrak{R}}_{1}$	${\mathfrak{R}}_{2}$	${\mathfrak{R}}_{3}$	${\mathfrak{R}}_{4}$	${\mathfrak{R}}_{5}$	${\mathfrak{R}}_{6}$
${\boldsymbol{\ss }}_{1}$	$$\left(\begin{array}{c}\left[0.26255,0.36828\right],\\ \left[\mathrm{0.85406,0.90378}\right]\end{array}\right)$$	$$\left(\begin{array}{c}\left[0.19132,0.28268\right],\\ \left[0.90786,0.93069\right]\end{array}\right)$$	$$\left(\begin{array}{c}\left[0.20186, 0.33030\right],\\ \left[0.85051,0.91667\right]\end{array}\right)$$	$$\left(\begin{array}{c}\left[0.18528, 0.37432\right],\\ \left[\mathrm{0.8588,0.94797}\right]\end{array}\right)$$	$$\left(\begin{array}{c}\left[\mathrm{0.16377,0.30537}\right],\\ \left[\mathrm{0.84693,0.94309}\right]\end{array}\right)$$	$$\left(\begin{array}{c}\left[\mathrm{0.12117,0.18909}\right],\\ \left[\mathrm{0.92454,0.96044}\right]\end{array}\right)$$
${\boldsymbol{\ss }}_{2}$	$$\left(\begin{array}{c}\left[0.21535, 0.36828\right],\\ \left[\mathrm{082635,0.89626}\right]\end{array}\right)$$	$$\left(\begin{array}{c}\left[0.16525,0.24505\right],\\ \left[0.90933,0.94743\right]\end{array}\right)$$	$$\left(\begin{array}{c}\left[0.18452,0.29078\right],\\ \left[0.86766,0.91759\right]\end{array}\right)$$	$$\left(\begin{array}{c}\left[0.14230 0.25814\right],\\ \left[\mathrm{0.92525,0.95132}\right]\end{array}\right)$$	$$\left(\begin{array}{c}\left[0.14475, 0.24961\right],\\ \left[\mathrm{0.88295,0.95045}\right]\end{array}\right)$$	$$\left(\begin{array}{c}\left[0.13379, 0.24841\right],\\ \left[\mathrm{0.92643,0.96547}\right]\end{array}\right)$$
${\boldsymbol{\ss }}_{3}$	$$\left(\begin{array}{c}\left[0.20547, 0.35635\right],\\ \left[\mathrm{0.83275,0.92174}\right]\end{array}\right)$$	$$\left(\begin{array}{c}\left[0.16637,0.23606\right],\\ \left[0.90666,0.94821\right]\end{array}\right)$$	$$\left(\begin{array}{c}\left[0.17565, 0.30153\right],\\ \left[0.80936,0.92561\right]\end{array}\right)$$	$$\left(\begin{array}{c}\left[0.08520, 0.23634\right],\\ \left[\mathrm{0.88547,0.94627}\right]\end{array}\right)$$	$$\left(\begin{array}{c}\left[\mathrm{0.09226,0.24656}\right],\\ \left[\mathrm{0.86386,0.93184}\right]\end{array}\right)$$	$$\left(\begin{array}{c}\left[\mathrm{0.12147,0.17492}\right],\\ \left[\mathrm{0.90621,0.97147}\right]\end{array}\right)$$
${\boldsymbol{\ss }}_{4}$	$$\left(\begin{array}{c}\left[0.20336, 0.34626\right],\\ \left[\mathrm{0.77953,0.91303}\right]\end{array}\right)$$	$$\left(\begin{array}{c}\left[0.14350,0.28653\right],\\ \left[0.90385,0.93975\right]\end{array}\right)$$	$$\left(\begin{array}{c}\left[0.16307,0.31925\right],\\ \left[0.83802,0.90816\right]\end{array}\right)$$	$$\left(\begin{array}{c}\left[0.11701, 0.25826\right],\\ \left[\mathrm{0.90815,0.96025}\right]\end{array}\right)$$	$$\left(\begin{array}{c}\left[\mathrm{0.09940,0.20004}\right],\\ \left[\mathrm{0.87805,0.94459}\right]\end{array}\right)$$	$$\left(\begin{array}{c}\left[\mathrm{0.09893,0.15697}\right],\\ \left[\mathrm{0.91081,0.98134}\right]\end{array}\right)$$
${\boldsymbol{\ss }}_{5}$	$$\left(\begin{array}{c}\left[0.21075, 0.37246\right],\\ \left[\mathrm{0.84992,0.88805}\right]\end{array}\right)$$	$$\left(\begin{array}{c}\left[0.15083,0.26583\right],\\ \left[0.93627,0.95243\right]\end{array}\right)$$	$$\left(\begin{array}{c}\left[0.14355,0.25377\right],\\ \left[0.84633,0.92193\right]\end{array}\right)$$	$$\left(\begin{array}{c}\left[0.14232, 0.20876\right],\\ \left[\mathrm{0.87588,0.96259}\right]\end{array}\right)$$	$$\left(\begin{array}{c}\left[\mathrm{0.14724,0.22008}\right],\\ \left[\mathrm{0.89347,0.96584}\right]\end{array}\right)$$	$$\left(\begin{array}{c}\left[\mathrm{0.07793,0.16009}\right],\\ \left[\mathrm{0.93630,0.97716}\right]\end{array}\right)$$

Step 5: Apply Eq. 8 to calculate an average solution matrix to obtain:

$${\left[{\text \AA }{S}_{h}\right]}_{1\times 6}$$

$$= \left[ \begin{gathered} \begin{array}{*{20}c} {\left( {\begin{array}{*{20}c} {\left[ {0.88755,0.95283} \right],} \\ {\left[ {0.85803,0.92089} \right]} \\ \end{array} } \right)} & {\left( {\begin{array}{*{20}c} {\left[ {0.84593,0.91305} \right],} \\ {\left[ {0.92775,0.95333} \right]} \\ \end{array} } \right)} & {\left( {\begin{array}{*{20}c} {\left[ {0.85464,0.93097} \right],} \\ {\left[ {0.86986,0.93205} \right]} \\ \end{array} } \right)} \\ \end{array} \hfill \\ \begin{array}{*{20}c} {\left( {\begin{array}{*{20}c} {\left[ {0.81716,0.90433} \right],} \\ {\left[ {0.90985,0.96155} \right]} \\ \end{array} } \right)} & {\left( {\begin{array}{*{20}c} {\left[ {0.81649,0.90359} \right],} \\ {\left[ {0.89506,0.95621} \right]} \\ \end{array} } \right)} & {\left( {\begin{array}{*{20}c} {\left[ {0.79443,0.86562} \right],} \\ {\left[ {0.93448,0.97605} \right]} \\ \end{array} } \right)} \\ \end{array} \hfill \\ \end{gathered} \right]$$

Step 6: Table 6 shows the results of Eqs. 9 and 10. These equations determine the PDA and NDA matrices’ necessary structure and mathematical calculation.

**Table 6:** PDA and NDA matrices.

𝑷𝑫Å𝑺	${\mathfrak{R}}_{1}$	${\mathfrak{R}}_{2}$	${\mathfrak{R}}_{3}$	${\mathfrak{R}}_{4}$	${\mathfrak{R}}_{5}$	${\mathfrak{R}}_{6}$
${\boldsymbol{\ss }}_{1}$	$$\left(\begin{array}{c}\left[-0.05760, 0.09849\right], \\ \left[-0.10503, -0.05187\right]\end{array}\right)$$	$$\left(\begin{array}{c}\left[\mathrm{0.0,0.0}\right],\\ \left[\mathrm{0.0,0.0}\right]\end{array}\right)$$	$$\left(\begin{array}{c}\left[\mathrm{0.0,0.0}\right],\\ \left[\mathrm{0.0,0.0}\right]\end{array}\right)$$	$$\left(\begin{array}{c}\left[-0.05660, 0.06123\right],\\ \left[-0.95943, -0.93816\right]\end{array}\right)$$	$$\left(\begin{array}{c}\left[\mathrm{0.07846,0.10611}\right],\\ \left[-0.99522, -0.96190\right]\end{array}\right)$$	$$\left(\begin{array}{c}\left[\mathrm{0.0,0.0}\right],\\ \left[\mathrm{0.0,0.0}\right]\end{array}\right)$$
${\boldsymbol{\ss }}_{2}$	$$\left(\begin{array}{c}\left[-0.05760, 0.02184\right], \\ \left[-0.06005, -0.03966\right]\end{array}\right)$$	$$\left(\begin{array}{c}\left[0.0, 0.0\right],\\ \left[\mathrm{0.0,0.0}\right]\end{array}\right)$$	$$\left(\begin{array}{c}\left[\mathrm{0.0,0.0}\right],\\ \left[\mathrm{0.0,0.0}\right]\end{array}\right)$$	$$\left(\begin{array}{c}\left[0.02668, 0.04017\right],\\ \left[-1.04590, -0.09425\right]\end{array}\right)$$	$$\left(\begin{array}{c}\left[0.03128, 0.05294\right],\\ \left[-1.04357, -0.97178\right]\end{array}\right)$$	$$\left(\begin{array}{c}\left[\mathrm{0.0,0.0}\right],\\ \left[\mathrm{0.0,0.0}\right]\end{array}\right)$$
${\boldsymbol{\ss }}_{3}$	$$\left(\begin{array}{c}\left[-0.00579, 0.03823\right], \\ \left[-0.08103, -0.07042\right]\end{array}\right)$$	$$\left(\begin{array}{c}\left[\mathrm{0.0,0.00746}\right],\\ \left[\mathrm{0.0,0.0}\right]\end{array}\right)$$	$$\left(\begin{array}{c}\left[\mathrm{0.0,0.0}\right],\\ \left[\mathrm{0.0,0.0}\right]\end{array}\right)$$	$$\left(\begin{array}{c}\left[0.0, 0.01179\right],\\ \left[-0.99412, -0.93595\right]\end{array}\right)$$	$$\left(\begin{array}{c}\left[0.0, 0.02719\right],\\ \left[-1.01795, -0.94680\right]\end{array}\right)$$	$$\left(\begin{array}{c}\left[\mathrm{0.0,0.0}\right],\\ \left[\mathrm{0.0,0.0}\right]\end{array}\right)$$
${\boldsymbol{\ss }}_{4}$	$$\left(\begin{array}{c}\left[0.00237, 0.02185\right],\\ \left[-0.06690, 0.0\right]\end{array}\right)$$	$$\left(\begin{array}{c}\left[0.0, 0.00868\right],\\ \left[\mathrm{0.0,0.0}\right]\end{array}\right)$$	$$\left(\begin{array}{c}\left[0.0, 0.0\right],\\ \left[\mathrm{0.0,0.0}\right]\end{array}\right)$$	$$\left(\begin{array}{c}\left[-0.04032, 0.0\right],\\ \left[-1.02365, -0.95416\right]\end{array}\right)$$	$$\left(\begin{array}{c}\left[0.0, 0.0\right],\\ \left[-1.03699, -0.96391\right]\end{array}\right)$$	$$\left(\begin{array}{c}\left[-0.0193, 0.00455\right],\\ \left[\mathrm{0.0,0.0}\right]\end{array}\right)$$
${\boldsymbol{\ss }}_{5}$	$$\left(\begin{array}{c}\left[0.01437, 0.06438\right],\\ \left[-0.09831, -0.02633\right]\end{array}\right)$$	$$\left(\begin{array}{c}\left[\mathrm{0.0,0.0}\right],\\ \left[\mathrm{0.0,0.0}\right]\end{array}\right)$$	$$\left(\begin{array}{c}\left[\mathrm{0.02454,0.03252}\right],\\ \left[\mathrm{0.0,0.0}\right]\end{array}\right)$$	$$\left(\begin{array}{c}\left[-0.02671, 0.0\right],\\ \left[-0.98164, -0.95721\right]\end{array}\right)$$	$$\left(\begin{array}{c}\left[0.0, 0.05627\right],\\ \left[-1.05768, -0.99243\right]\end{array}\right)$$	$$\left(\begin{array}{c}\left[0.01445, 0.02978\right],\\ \left[\mathrm{0.0,0.0}\right]\end{array}\right)$$
$${{N}}{{D}}{\AA }{{S}}$$						
${\boldsymbol{\ss }}_{1}$	$$\left(\begin{array}{c}\left[\mathrm{0.0,0.0}\right], \\ \left[\mathrm{0.0,0.0}\right]\end{array}\right)$$	$$\left(\begin{array}{c}\left[-0.05347,-0.05312\right],\\ \left[-0.03415, 0.01634\right]\end{array}\right)$$	$$\left(\begin{array}{c}\left[-0.08255, -0.06313\right],\\ \left[-0.07101, 0.04497\right]\end{array}\right)$$	$$\left(\begin{array}{c}\left[\mathrm{0.0,0.0}\right],\\ \left[\mathrm{0.0,0.0}\right]\end{array}\right)$$	$$\left(\begin{array}{c}\left[\mathrm{0.0,0.0}\right],\\ \left[\mathrm{0.0,0.0}\right]\end{array}\right)$$	$$\left(\begin{array}{c}\left[-0.02477, 0.02156\right],\\ \left[-0.04052, 0.00714\right]\end{array}\right)$$
${\boldsymbol{\ss }}_{2}$	$$\left(\begin{array}{c}\left[\mathrm{0.0,0.0}\right], \\ \left[\mathrm{0.0,0.0}\right]\end{array}\right)$$	$$\left(\begin{array}{c}\left[-0.01959, -0.00422\right],\\ \left[-0.03809, -0.03606\right]\end{array}\right)$$	$$\left(\begin{array}{c}\left[-0.03706, 0.02314\right],\\ \left[-0.09680,-0.04635\right]\end{array}\right)$$	$$\left(\begin{array}{c}\left[\mathrm{0.0,0.0}\right],\\ \left[\mathrm{0.0,0.0}\right]\end{array}\right)$$	$$\left(\begin{array}{c}\left[\mathrm{0.0,0.0}\right],\\ \left[\mathrm{0.0,0.0}\right]\end{array}\right)$$	$$\left(\begin{array}{c}\left[-0.10115, 0.04169\right],\\ \left[-0.04279, 0.01318\right]\end{array}\right)$$
${\boldsymbol{\ss }}_{3}$	$$\left(\begin{array}{c}\left[\mathrm{0.0,0.0}\right], \\ \left[\mathrm{0.0,0.0}\right]\end{array}\right)$$	$$\left(\begin{array}{c}\left[-\mathrm{0.02105,0.0}\right],\\ \left[-0.03910, -0.32590,\right]\end{array}\right)$$	$$\left(\begin{array}{c}\left[-0.03929, -0.02373\right],\\ \left[-0.0584, -0.00913\right]\end{array}\right)$$	$$\left(\begin{array}{c}\left[-0.04766, 0.0\right],\\ \left[\mathrm{0.0,0.0}\right]\end{array}\right)$$	$$\left(\begin{array}{c}\left[-0.01701, 0.0\right],\\ \left[\mathrm{0.0,0.0}\right]\end{array}\right)$$	$$\left(\begin{array}{c}\left[-0.02516, 0.00253\right],\\ \left[-0.01848, 0.02039\right]\end{array}\right)$$
${\boldsymbol{\ss }}_{4}$	$$\left(\begin{array}{c}\left[\mathrm{0.0,0.0}\right],\\ \left[-0.016, 0.0\right]\end{array}\right)$$	$$\left(\begin{array}{c}\left[-0.05812, 0.0\right],\\ \left[-0.03259, 0.02811\right]\end{array}\right)$$	$$\left(\begin{array}{c}\left[-0.06595, -0.00481\right],\\ \left[-0.05223, -0.03217\right]\end{array}\right)$$	$$\left(\begin{array}{c}\left[-0.00624, 0.0\right],\\ \left[\mathrm{0.0,0.0}\right]\end{array}\right)$$	$$\left(\begin{array}{c}\left[-0.03524, -0.00792\right],\\ \left[\mathrm{0.0,0.0}\right]\end{array}\right)$$	$$\left(\begin{array}{c}\left[\mathrm{0.0,0.0}\right],\\ \left[-0.03225, -0.02402\right]\end{array}\right)$$
${\boldsymbol{\ss }}_{5}$	$$\left(\begin{array}{c}\left[\mathrm{0.0,0.0}\right],\\ \left[\mathrm{0.0,0.0}\right]\end{array}\right)$$	$$\left(\begin{array}{c}\left[-0.03122, -0.00085\right],\\ \left[-0.028093, 0.04459\right]\end{array}\right)$$	$$\left(\begin{array}{c}\left[\mathrm{0.0,0.0}\right],\\ \left[-0.06472, 0.05287\right]\end{array}\right)$$	$$\left(\begin{array}{c}\left[-0.02412, 0.0\right],\\ \left[\mathrm{0.0,0.0}\right]\end{array}\right)$$	$$\left(\begin{array}{c}\left[-0.00834, 0.0\right],\\ \left[\mathrm{0.0,0.0}\right]\end{array}\right)$$	$$\left(\begin{array}{c}\left[\mathrm{0.0,0.0}\right],\\ \left[-0.05465, 0.02723\right]\end{array}\right)$$

Step 7: The positive and negative weighted distances for the alternatives are calculated. Particularly, we used Eq. 11 to find the positive distances (${\rm P}_{\tau }$: $\tau =\mathrm{1,2},\mathrm{3,4},5$) of alternatives for IVPFSEHWA operator and Eq. 12 to find the negative distances (${Q}_{\tau }$: $\tau =\mathrm{1,2},\mathrm{3,4},5$) of alternatives for the IVPFSEOWA operator. These weighted distance measures compute the relative dominance and dependency of each alternative with respect to the average solution. The obtained values are given as:

$${\rm P}_{1}=\left(\left[0.48927, 0.79001\right], \left[0.20172, 0.45602\right]\right),$$

$${\rm P}_{2}=\left(\left[0.71723, 0.89060\right], \left[0.40492, 0.44324\right]\right),$$

$${\rm P}_{3}=\left(\left[0.65675, 0.94178\right], \left[0.22449, 0.33602\right]\right),$$

$${\rm P}_{4}=\left(\left[ 0.01529, 0.55318\right], \left[0.35568, 0.69645\right]\right),$$

$${\rm P}_{5}=\left(\left[0.12776, 0.93086\right], \left[0.38473, 0.57988\right]\right),$$

$${Q}_{1}=\left(\left[0.92500, 0.99017\right], \left[0.14001, 0.23203\right]\right),$$

$${Q}_{2}=\left(\left[0.051165, 0.94577\right], \left[0.18442, 0.2361\right]\right),$$

$${Q}_{3}=\left(\left[0.08726, 0.96282\right], \left[0.09758, 0.22794\right]\right),$$

$${Q}_{4}=\left(\left[0.08293, 0.98659\right], \left[0.07141, 0.57516\right]\right),$$

And $${Q}_{5}=\left(\left[0.05599, 0.95889\right], \left[0.11992, 0.24083\right]\right)$$.

Also, the corresponding values for the IVPFSEHWG and IVPFSEOWG operators are calculated using Eqs. (13) and (14).

Step 8: Consequently, we use the equations. (15) and (16) to normalize the weighted distance values of the ${\rm P}_{\tau }$ and ${Q}_{\tau }$. The normalized positive and negative assessment values are also presented as follows:

$$N{\rm P}_{1}=\left(\left[0.68217, 0.83885\right], \left[0.49817, 0.65478\right]\right),$$

$$N{\rm P}_{2}=\left(\left[0.94566, 1.0\right], \left[0.63643, 1.0\right]\right),$$

$$N{\rm P}_{3}=\left(\left[0.91567, 1.0\right], \left[0.48248, 0.55441\right]\right),$$

$$N{\rm P}_{4}=\left(\left[0.021318, 0.58738\right], \left[0.87840, 1.0\right]\right),$$

$$N{\rm P}_{5}=\left(\left[0.17813, 0.98840\right], \left[0.83262, 0.95014\right]\right),$$

$$N{Q}_{1}=\left(\left[0.0, 0.0\right], \left[0.24081, 0.59658\right]\right),$$

$$N{Q}_{2}=\left(\left[0.04491, 0.94469\right], \left[0.0, 0.58951\right]\right),$$

$$N{Q}_{3}=\left(\left[0.02762, 0.90566\right], \left[0.47088, 0.59658\right]\right),$$

$$N{Q}_{4}=\left(\left[0.00362, 0.91035\right], \left[0.0, 0.61424\right]\right),$$

And $${NQ}_{5}=\left(\left[0.03159, 0.93947\right], \left[0.34975, 0.58128\right]\right)$$.

Step 9: Evaluate the integrative appraisal score $I$ employing Eq. 17 as stated:

$${I}_{1}=\left(\left[0.34108, 0.41942\right], \left[0.09178, 0.33399\right]\right),$$

$${I}_{2}=\left(\left[0.47294, 0.5\right], \left[0.0, 0.31843\right]\right),$$

$${I}_{3}=\left(\left[0.49568, 0.5\right], \left[0.21043, 0.23548\right]\right),$$

$${I}_{4}=\left(\left[0.29369, 0.45521\right], \left[0.0, 0.50484\right]\right),$$

And $${I}_{5}=\left(\left[0.47155, 0.49421\right], \left[0.31897, 0.44125\right]\right)$$.

$${I}_{1}=0.49027, {I}_{2}=0.62862,$$

$${I}_{3}=0.57295, {I}_{4}=0.52561,$$

and $${I}_{5}=0.75768$$.

Step 10: Afterwards, the potential choices are arranged in descending order based on their appraisal scores. The highest-ranked option is the most superior, while the lowest-ranked one is the least favorable alternative ${{\bar{\mathrm{U}}}}_{5}>{{\bar{\mathrm{U}}}}_{2}>{{\bar{\mathrm{U}}}}_{3}>{{\bar{\mathrm{U}}}}_{4}>{{\bar{\mathrm{U}}}}_{1}$.

This suggested approach provides substantial improvements over the well-established EDAS approach. While the current EDAS approach expresses the mean difference of the assessed facts using real numbers, the suggested technique represents these values. Therefore, the proposed paradigm allows experts to articulate their viewpoints more flexibly.

## Comparative analysis and discussion

The following section compares our model with other models, structures, and operators.

### Comparative analysis

The evaluation of our proposed method will be done in three different ways. First, a comparison is made between the new EDAS method and existing fuzzy model-based EDAS methods. Next, the proposed EDAS approach is evaluated against existing aggregation operators within the IVPFSS framework. Finally, the methods are compared with the TOPSIS and MCDM techniques within the IVPFSS framework to validate our approach.

#### Comparative analysis of the proposed model with EDAS approaches in different structures

Specifically, classical approaches, including fuzzy EDAS^49^ and IVFS EDAS^50^, primarily focused on the MD or the interval for each option. Nevertheless, the IFS EDAS^51^ and IVIFS EDAS^52^ methods perform inspections that incorporate both MD and NMD. The PFS EDAS process was proposed by Liu et al.^53^, and the IVPFS EDAS framework was laid out by Yanmaz et al.^55^. This method, however, will still need to address the issue of achieving a good interaction with the parameterization of prospects. The comparison of our suggested EDAS approach and the existing EDAS methodologies, which work under various fuzzy set frameworks, is shown in Table 7, which presents an empirical investigation.

**Table 7:** Comparison with prevailing EDAS models.

Structure	Appraisal score					Ranking
Fuzzy EDAS^49^	n/a					n/a
Interval fuzzy EDAS^50^	n/a					n/a
IFS EDAS^51^	n/a					n/a
IVIFS EDAS^52^	n/a					n/a
PFS EDAS^53^	n/a					n/a
IVPFS EDAS^55^	n/a					n/a
Proposed EDAS	0.49027	0.62862	0.57295	0.52561	0.757680.75768	${{\bar{\mathrm{U}}}}_{5}>{{\bar{\mathrm{U}}}}_{2}>{{\bar{\mathrm{U}}}}_{3}>{{\bar{\mathrm{U}}}}_{4}>{{\bar{\mathrm{U}}}}_{1}$

The proposed EDAS model solves the main problems of traditional EDAS methods, especially in parameter selection. It is worth noting that it is practical as an information source and that its reliability is evident in its practical application. The results in Table 7 show that this method performs very well in selecting the best option from all available options.

#### Comparison with different aggregation operators

**Fig. 3:**
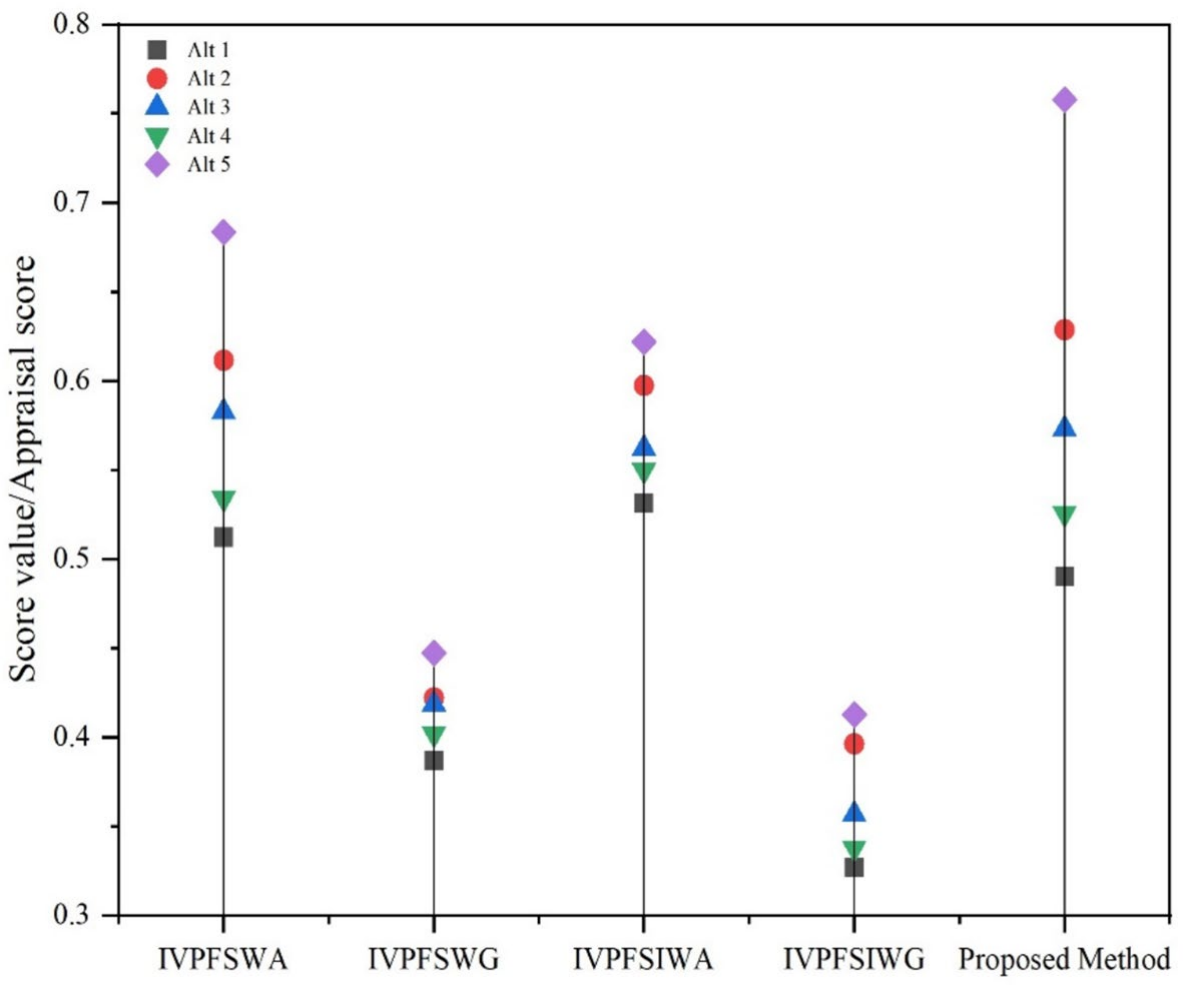
Comparison against various operators.

The comparative research focuses on AOs to examine and clarify the significant benefits of the planned approach. The primary objective of the analysis is to review the developed model with multiple operators, such as IVPFSWA^21^, IVPFSWG^21^, IVPFSIWA^35^, and IVPFSIWG^35^. The IVPFSEOWA operator is intentionally altered into every desired operator in Step 2 of the numerical illustration stated in Sect. 7.4 to boost its precision. Furthermore, the additional information from assessing remains identical. The outcomes listed in Table 8 and Fig. 3 confirm the consequences of implementing several operators. Table 8 clarifies that the ordering methods of organic food suppliers vary depending on the method used to assemble the subsequent evaluation details. Although some analytical variation, the outcomes are still fairly stable, with ${{\bar{\mathrm{U}}}}_{5}$ frequently dominating the place of the most effective supplier, proving the practicality of the recommended approach.

**Table 8:** Ranking of alternatives using distinct AOs in the same structure.

Structure	Alternatives score values/appraisal score	Ranking
IVPFSWA^21^	$S\left({{\bar{\mathrm{U}}}}_{1}\right)$= 0.51213, $S\left({{\bar{\mathrm{U}}}}_{2}\right)$ = 0.61147, $S\left({{\bar{\mathrm{U}}}}_{3}\right)$ = 0.58264, $S\left({{\bar{\mathrm{U}}}}_{4}\right)$ = 0.53415, and $S\left({{\bar{\mathrm{U}}}}_{5}\right)$ = 0.68345	${{\bar{\mathrm{U}}}}_{5}>{{\bar{\mathrm{U}}}}_{2}>{{\bar{\mathrm{U}}}}_{3}>{{\bar{\mathrm{U}}}}_{4}>{{\bar{\mathrm{U}}}}_{1}$
IVPFSWG^21^	$S\left({{\bar{\mathrm{U}}}}_{1}\right)$= 0.38672, $S\left({{\bar{\mathrm{U}}}}_{2}\right)$ = 0.42198, $S\left({{\bar{\mathrm{U}}}}_{3}\right)$ = 0.41856, $S\left({{\bar{\mathrm{U}}}}_{4}\right)$ = 0.40196, and $S\left({{\bar{\mathrm{U}}}}_{5}\right)$ = 0.44712	${{\bar{\mathrm{U}}}}_{5}>{{\bar{\mathrm{U}}}}_{2}>{{\bar{\mathrm{U}}}}_{3}>{{\bar{\mathrm{U}}}}_{4}>{{\bar{\mathrm{U}}}}_{1}$
IVPFSIWA^35^	$S\left({{\bar{\mathrm{U}}}}_{1}\right)$= 0.53129, $S\left({{\bar{\mathrm{U}}}}_{2}\right)$ = 0.59736, $S\left({{\bar{\mathrm{U}}}}_{3}\right)$ = 0.56214, $S\left({{\bar{\mathrm{U}}}}_{4}\right)$ = 0.54981, and $S\left({{\bar{\mathrm{U}}}}_{5}\right)$ = 0.62184	${{\bar{\mathrm{U}}}}_{5}>{{\bar{\mathrm{U}}}}_{2}>{{\bar{\mathrm{U}}}}_{3}>{{\bar{\mathrm{U}}}}_{4}>{{\bar{\mathrm{U}}}}_{1}$
IVPFSIWG^35^	$S\left({{\bar{\mathrm{U}}}}_{1}\right)$= 0.32693, $S\left({{\bar{\mathrm{U}}}}_{2}\right)$ = 0.39627, $S\left({{\bar{\mathrm{U}}}}_{3}\right)$ = 0.35672, $S\left({{\bar{\mathrm{U}}}}_{4}\right)$ = 0.33781, and $S\left({{\bar{\mathrm{U}}}}_{5}\right)$ = 0.41265	${{\bar{\mathrm{U}}}}_{5}>{{\bar{\mathrm{U}}}}_{2}>{{\bar{\mathrm{U}}}}_{3}>{{\bar{\mathrm{U}}}}_{4}>{{\bar{\mathrm{U}}}}_{1}$
Proposed Method	$I\left({{\bar{\mathrm{U}}}}_{1}\right)$= 0.49027, $I\left({{\bar{\mathrm{U}}}}_{2}\right)$ = 0.62862, $I\left({{\bar{\mathrm{U}}}}_{3}\right)$ = 0.57295, $I\left({{\bar{\mathrm{U}}}}_{4}\right)$ = 0.52561, and $I\left({{\bar{\mathrm{U}}}}_{5}\right)$ = 0.75768	${{\bar{\mathrm{U}}}}_{5}>{{\bar{\mathrm{U}}}}_{2}>{{\bar{\mathrm{U}}}}_{3}>{{\bar{\mathrm{U}}}}_{4}>{{\bar{\mathrm{U}}}}_{1}$

The results suggest that this technique is the most advantageous, as it is supported by numerous scholars, thereby enhancing its credibility and practicality for identifying conscious suppliers. The results indicate beyond a doubt that the mentioned tactic is feasible. On the other hand, some differences in the ranking order were observed, and these are likely due to the particular implementation of the Einstein operations within the overall approach. These tactics are shown to be very powerful in spotting not only complex and unclear patterns in the data set, but also without going along with the risk of errors or enhancements. The earlier operators overlooked the need to gather more trustworthy, often varying information at that time, which might explain the differences in the ranking of the results. Given that the development of intelligent operators capable of dealing with fluctuating and intricate aspects in DM is a must, it is accordingly important to verify the dependability of our proposed method.

#### Comparison with different decision-making techniques in the same structure

A comparative analysis of the proposed EDAS model with two existing methods: the TOPSIS method^36^ and the MCDM technique^60^. A clear pattern emerges across all three approaches: alternative ${{\bar{\mathrm{U}}}}_{5}$ regularly attains the maximum appraisal score or closeness coefficient, while alternative ${{\bar{\mathrm{U}}}}_{2}$ is ranked second in all approaches. This consistency shows that these two suppliers are comparatively robust choices and that the planned EDAS method aligns with earlier approaches for identifying the best options. But some modifications are based on the rank of the remaining alternatives. The ranking order is ${{\bar{\mathrm{U}}}}_{5}>{{\bar{\mathrm{U}}}}_{2}>{{\bar{\mathrm{U}}}}_{1}>{{\bar{\mathrm{U}}}}_{4}>{{\bar{\mathrm{U}}}}_{3}$ using the TOPIS technique​, whereas both the MCDM and the proposed EDAS model generate the same ranking ${{\bar{\mathrm{U}}}}_{5}>{{\bar{\mathrm{U}}}}_{2}>{{\bar{\mathrm{U}}}}_{3}>{{\bar{\mathrm{U}}}}_{4}>{{\bar{\mathrm{U}}}}_{1}$. which supports assurance in the consistency and rationality of the planned EDAS model. This variation suggests that TOPSIS and EDAS differ in how they treat the relative performance of middle- and lower-ranked alternatives.

Table 9 presents the final ranking of the proposed EDAS method, thereby improving the reliability of supplier selection. The existing TOPSIS and MCDM techniques use mathematical methods to determine the best alternative. The difference in this concept shows that our method is powerful and reliable, as it points to the lowest-cost option, while at the same time, we need to find suppliers safely and appropriately through sustainable practices.

**Table 9:** Comparison with the TOPSIS method.

Method	Appraisal score/closeness coefficient					Ranking
TOPSIS method^36^	0.62947	0.67391	0.52813	0.58209	0.71539	${{\bar{\mathrm{U}}}}_{5}>{{\bar{\mathrm{U}}}}_{2}>{{\bar{\mathrm{U}}}}_{1}>{{\bar{\mathrm{U}}}}_{4}>{{\bar{\mathrm{U}}}}_{3}$
MCDM technique^60^	0.50962	0.54138	0.53708	0.51862	0.56094	${{\bar{\mathrm{U}}}}_{5}>{{\bar{\mathrm{U}}}}_{2}>{{\bar{\mathrm{U}}}}_{3}>{{\bar{\mathrm{U}}}}_{4}>{{\bar{\mathrm{U}}}}_{1}$
Proposed EDAS	0.49027	0.62862	0.57295	0.52561	0.75768	${{\bar{\mathrm{U}}}}_{5}>{{\bar{\mathrm{U}}}}_{2}>{{\bar{\mathrm{U}}}}_{3}>{{\bar{\mathrm{U}}}}_{4}>{{\bar{\mathrm{U}}}}_{1}$

**Fig. 4:**
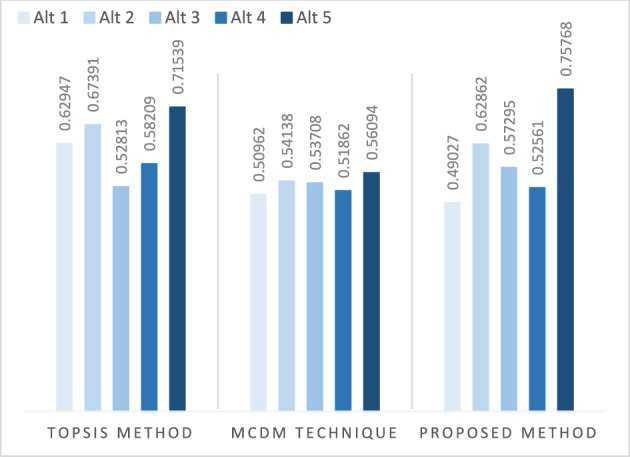
Comparison with TOPSIS and MCDM techniques.

Figure 4 shows the schematic diagram of the proposed method and the TOPSIS method^36^.

The results from our recommended method are clearly superior to those of the existing TOPSIS method, making our method more reliable than traditional methods for addressing complex practical decision-making problems.

### Discussion and implications

In this subsection, we discuss the results of our proposed model compared with other decision-making techniques. Moreover, we explore the implications and benefits of our developed EDAS model.

#### Discussion

We confirmed that the proposed EDAS model is effectively comparable to prevailing research by observing the closeness of its outputs to those of existing methods. This method provides a better, more comprehensive perspective on the decision-making management environment by fully leveraging information related to attributes. One of the most remarkable advantages of this method is that it can provide more accurate guidance on determining the boundaries of each option, thereby helping maintain data consistency. Establishing high-quality, reliable data is a great improvement that makes this method more useful in the decision-making process.

The proposed EDAS model systematically assesses all prospective suppliers to select sustainable suppliers. The EDAS method, developed by Ghorabaee^49^, Ilieva^50^, Mishra et al.^51^, and Liu et al.^53^, still faces multiple complications during execution, including MD and NMD intervals and parametrization. Also, the DM techniques proposed by Peng and Yang^16^, Liu et al.^43^, and Rahman et al.^47,48^ can handle MD and NMD intervals; however, they still fail to combine the parametrizations of the alternatives according to the average solution matrix.

Li and Wang^52^ and Yanmaz et al.^55^ developed the EDAS method to accurately identify the best alternative by integrating the mean solution matrix with MD and NMD intervals. This also highlights the difference between our methods, demonstrating that our proposed method has a stronger ability to handle complex decision makers (DMs) and provides more reliable, accurate results than traditional methods.

Nonetheless, these frameworks still fail to address option parameterized assessment properly. Jiang et al.^19^ and Zulqarnain et al.^21^ respectively combined DM techniques with the IVIFSS and IVPFSS to overcome those limitations. The issues referred to above were solved using these techniques. They generated a large number of AOs, and various features of them were identified, as well as DM methods tailored to situations where ${MD}^{\mho }+{NMD}^{\mho }>1$. Besides, these innovations have significantly increased the ability to evaluate complex options more accurately.

#### Benefits and theoretical implementations of the developed EDAS technique

It is challenging to select the best company for preserving healthy food sources. To address this issue, a model was developed that considered the perspectives on sustainable supplier management and its key characteristics. With this technique, the assessment results will be trustworthy and dependable.

To get a precise assessment of the alternatives, we used the EDAS method. During the whole process, ${{\bar{\mathrm{U}}}}_{5}$ was the strongest option, with ${{\bar{\mathrm{U}}}}_{2}$, ${{\bar{\mathrm{U}}}}_{3}$, ${{\bar{\mathrm{U}}}}_{4}$, and ${{\bar{\mathrm{U}}}}_{1}$ coming in that sequence of preference, that is, ${{\bar{\mathrm{U}}}}_{5}>{{\bar{\mathrm{U}}}}_{2}>{{\bar{\mathrm{U}}}}_{3}>{{\bar{\mathrm{U}}}}_{4}>{{\bar{\mathrm{U}}}}_{1}$. It should not be forgotten that using different sets of attribute weightings can impact the order of the outcomes. However, it is still worth noting that ${{\bar{\mathrm{U}}}}_{5}$ has always been proven to be the most effective choice.

The analysis of the operational capabilities of sustainable suppliers and their competitors provides municipal officials with valuable insights to inform decision-making and develop projects that enhance the community’s quality of life.

In unpredictable situations, the EDAS method effectively identifies appropriate organizations to address the sustainable supplier problem. Decision support tools, combined with structured methods, will significantly improve evaluation accuracy and reduce the organization’s workload. It is recommended to integrate specialised software to optimize the DM management process, thereby reducing the time and labour required to resolve such issues.

This research delivers multiple important benefits for managers involved in sustainable food supply chains, proposing actionable policies to expand decision-making in supplier selection and sustainability recital monitoring.

1. The proposed EDAS technique provides a robust mechanism to evaluate suppliers based on three main factors: environmental impact, social responsibility, and economic viability. Managers make more precise, data-driven decisions in supplier selection by integrating the EDAS model with Einstein-ordered/hybrid AOs for IVPFSS.
2. One of the major problems for managers is the growing market circumstances, governing variations, and management conflicts in dealing with sustainable food supply chains. The proposed EDAS model helps managers integrate real-time data into their decision-making processes, enabling continuous supplier evaluation.
3. Executives can use the developed EDAS model to evaluate the potential risks posed by suppliers, such as non-compliance and disturbances in fabrication due to climate-related measures.
4. The proposed EDAS technique provides a structured methodology for maintaining suppliers’ enduring presence in key sectors such as waste reduction, carbon footprint reduction, and community engagement.
5. Sustainable supplier selection is not just about reducing environmental impact; it is also about fostering innovation and collaboration with suppliers. Stakeholders can use the proposed model to identify committed suppliers of sustainable technologies, such as green packaging or renewable energy, for production processes.

### Limitations of the developed aggregation operators and EDAS model in IVPFSS

The implementation of the Einstein-ordered/hybrid AOs-based EDAS model for IVPFSS offers clear advantages that improve overall performance. This method not only optimizes the DM process but also enhances the system’s stability and reliability. However, people cannot ignore the inherent limitations of this process. The following limitations must be carefully evaluated to accurately assess the model’s potential and identify areas for further improvement.

1. The Einstein-ordered and hybrid AOs depend on the ordering of inputs and balancing coefficients that control the trade-off between weighted and ordered aggregation, respectively.
2. Different techniques for computing the criteria weights can alter the impact of the evaluation mechanism and may lead to ranking sensitivity in marginal cases.
3. The IVPFSS effectively handles uncertainty via MD and NMD intervals, but this structure still faces operational viability constraints and depends on the score/accuracy functions for ranking.
4. The selection of relevant, readily available data is a key factor in the successful application of the developed EDAS method. Any inconsistency or defect in the report information, especially in the sustainable supplier management system and expert opinions, will affect the validity of the research results.
5. We used the criteria weights in this study, which reflect the experts’ relative importance; in future studies, we plan to compute the criteria weights using the CRITIC/AHP/entropy methods when reliable quantitative data are available.
6. As mentioned earlier, this methodology shows that outsiders will not affect the characteristics in the decision-making process. However, in practice, these powers often show interdependence. Excluding these interactions may lead to a decline in the correctness and efficiency of decision-making methods.
7. Although the proposed EDAS model organizes expert familiarity, the eminence and consistency of the outcomes still depend on the decision-making capability, experience, and reliability of the participating assessors. Future studies may expand the size and diversity of the expert panel and examine alternative mechanisms for determining expert weights to further improve robustness.

## Conclusion

This research explores the sustainable food suppliers using social, economic, and environmental criteria, demonstrating the methodology’s relevance to real-world issues and providing a reliable foundation for sustainable supply chain management. The primary objective of this research is to define the Einstein operational laws for IVPFSS and several AOs using these operational laws, such as IVPFSEOWA, IVPFSEHWA, IVPFSEOWG, and IVPFSEHWG, and to examine their desirable properties. Moreover, a novel decision-support model, the Evaluation based on the Distance from the Average Solution (EDAS), is developed for sustainable food supplier selection using the Einstein ordered/hybrid AOs within the IVPFSS structure. The proposed EDAS model effectively addresses internal challenges and uncertainties in MAGDM, thereby enhancing DM performance and reliability. A case study is conducted to evaluate the effectiveness of the newly established EDAS method for identifying the optimal sustainable supplier in food supply chain management. The comparative analysis illustrated the benefits of the planned technique over traditional approaches, highlighting its versatility, computational effectiveness, and potential to identify intricate attribute interactions. The outcomes highlighted its ability to deliver accurate, dependable answers, making it an essential tool for decision-makers in sustainable supply chain management. This study comprises real-world tools that help experts make more informed, transparent, and sustainable decisions, ultimately leading to the development of more robust, globally responsible food supply chains.

Future research could enhance our proposed framework by integrating it with other decision-making models, such as the Analytic Hierarchy Process (AHP) or the Best–Worst Method (BWM), to refine supplier selection across various industries. Incorporating machine learning algorithms could enable dynamic updates to weights and criteria based on real-time supplier data, thereby improving the framework’s adaptability. Extending the framework to multi-tier supply chains would provide deeper insights into supplier interdependencies, helping businesses make more informed decisions. Additionally, while the framework was applied to sustainable food supply chains, it could be adapted to other sectors, such as healthcare, manufacturing, and retail. Lastly, exploring the inclusion of more complex uncertainty models, such as fuzzy credibility-based or hesitant fuzzy sets, could further strengthen the framework’s ability to handle imprecise data and enhance its robustness across diverse decision-making scenarios.

## Data Availability

All the data used and analyzed is available in the manuscript.

## Appendix 1

### Proof:

The presented theorem can be explored via mathematical induction.

For $n=1$, we get ${\partial }_{i}=1$. So,

$$IVPFSEOWA\left({\Delta }_{{a}_{11}},{\Delta }_{{a}_{12}},\dots \dots {\Delta }_{{a}_{1m}}\right)$$

$$=\stackrel{m}{\underset{j=1}{{\oplus }_{\varepsilon }}}{\Upsilon}_{j}{\Delta }_{p\left({a}_{{t^{\prime}}\left(1\right)z\left(j\right)}\right)}$$

$$=\left(\begin{array}{c}\left[\begin{array}{c}\frac{\sqrt{\prod_{j=1}^{m}{\left(1+{\left({\lambda }_{p\left({a}_{{t^{\prime}}\left(1\right)z\left(j\right)}\right)}^{l}\right)}^{2}\right)}^{{\Upsilon}_{j}}-\prod_{j=1}^{m}{\left(1-{\left({\lambda }_{p\left({a}_{{t^{\prime}}\left(1\right)z\left(j\right)}\right)}^{l}\right)}^{2}\right)}^{{\Upsilon}_{j}}}}{\sqrt{\prod_{j=1}^{m}{\left(1+{\left({\lambda }_{p\left({a}_{{t^{\prime}}\left(1\right)z\left(j\right)}\right)}^{l}\right)}^{2}\right)}^{{\Upsilon}_{j}}+\prod_{j=1}^{m}{\left(1-{\left({\lambda }_{p\left({a}_{{t^{\prime}}\left(1\right)z\left(j\right)}\right)}^{l}\right)}^{2}\right)}^{{\Upsilon}_{j}}}},\\ \frac{\sqrt{\prod_{j=1}^{m}{\left(1+{\left({\lambda }_{p\left({a}_{{t^{\prime}}\left(1\right)z\left(j\right)}\right)}^{\mathpzc{u}}\right)}^{2}\right)}^{{\Upsilon}_{j}}-\prod_{j=1}^{m}{\left(1+{\left({\lambda }_{p\left({a}_{{t^{\prime}}\left(1\right)z\left(j\right)}\right)}^{\mathpzc{u}}\right)}^{2}\right)}^{{\Upsilon}_{j}}}}{\sqrt{\prod_{j=1}^{m}{\left(1+{\left({\lambda }_{p\left({a}_{{t^{\prime}}\left(1\right)z\left(j\right)}\right)}^{\mathpzc{u}}\right)}^{2}\right)}^{{\Upsilon}_{j}}+\prod_{j=1}^{m}{\left(1+{\left({\lambda }_{p\left({a}_{{t^{\prime}}\left(1\right)z\left(j\right)}\right)}^{\mathpzc{u}}\right)}^{2}\right)}^{{\Upsilon}_{j}}}}\end{array}\right], \\ \left[\begin{array}{c}\frac{\sqrt{2\prod_{j=1}^{m}{\left({\left({\eta }_{p\left({a}_{{t^{\prime}}\left(1\right)z\left(j\right)}\right)}^{l}\right)}^{2}\right)}^{{\Upsilon}_{j}}}}{\sqrt{\prod_{j=1}^{m}{\left(2-{\left({\eta }_{p\left({a}_{{t^{\prime}}\left(1\right)z\left(j\right)}\right)}^{l}\right)}^{2}\right)}^{{\Upsilon}_{j}}+\prod_{j=1}^{m}{\left({\left({\eta }_{p\left({a}_{{t^{\prime}}\left(1\right)z\left(j\right)}\right)}^{l}\right)}^{2}\right)}^{{\Upsilon}_{j}}}},\\ \frac{\sqrt{2\prod_{j=1}^{m}{\left({\left({\eta }_{p\left({a}_{{t^{\prime}}\left(1\right)z\left(j\right)}\right)}^{\mathpzc{u}}\right)}^{2}\right)}^{{\Upsilon}_{j}}}}{\sqrt{\prod_{j=1}^{m}{\left(2-{\left({\eta }_{p\left({a}_{{t^{\prime}}\left(1\right)z\left(j\right)}\right)}^{\mathpzc{u}}\right)}^{2}\right)}^{{\Upsilon}_{j}}+\prod_{j=1}^{m}{\left({\left({\eta }_{p\left({a}_{{t^{\prime}}\left(1\right)z\left(j\right)}\right)}^{\mathpzc{u}}\right)}^{2}\right)}^{{\Upsilon}_{j}}}}\end{array}\right]\end{array}\right)$$

$$=\left(\begin{array}{c}\left[\begin{array}{c}\frac{\sqrt{\prod_{j=1}^{m}{\left(\prod_{i=1}^{1}{\left(1+{\left({\lambda }_{p\left({a}_{{t^{\prime}}\left(i\right)z\left(j\right)}\right)}^{l}\right)}^{2}\right)}^{{\partial }_{i}}\right)}^{{\Upsilon}_{j}}-\prod_{j=1}^{m}{\left(\prod_{i=1}^{1}{\left(1-{\left({\lambda }_{p\left({a}_{{t^{\prime}}\left(i\right)z\left(j\right)}\right)}^{l}\right)}^{2}\right)}^{{\partial }_{i}}\right)}^{{\Upsilon}_{j}}}}{\sqrt{\prod_{j=1}^{m}{\left(\prod_{i=1}^{1}{\left(1+{\left({\lambda }_{p\left({a}_{{t^{\prime}}\left(i\right)z\left(j\right)}\right)}^{l}\right)}^{2}\right)}^{{\partial }_{i}}\right)}^{{\Upsilon}_{j}}+\prod_{j=1}^{m}{\left(\prod_{i=1}^{1}{\left(1-{\left({\lambda }_{p\left({a}_{{t^{\prime}}\left(i\right)z\left(j\right)}\right)}^{l}\right)}^{2}\right)}^{{\partial }_{i}}\right)}^{{\Upsilon}_{j}}}},\\ \frac{\sqrt{\prod_{j=1}^{m}{\left(\prod_{i=1}^{1}{\left(1+{\left({\lambda }_{p\left({a}_{{t^{\prime}}\left(i\right)z\left(j\right)}\right)}^{\mathpzc{u}}\right)}^{2}\right)}^{{\partial }_{i}}\right)}^{{\Upsilon}_{j}}-\prod_{j=1}^{m}{\left(\prod_{i=1}^{1}{\left(1-{\left({\lambda }_{p\left({a}_{{t^{\prime}}\left(i\right)z\left(j\right)}\right)}^{\mathpzc{u}}\right)}^{2}\right)}^{{\partial }_{i}}\right)}^{{\Upsilon}_{j}}}}{\sqrt{\prod_{j=1}^{m}{\left(\prod_{i=1}^{1}{\left(1+{\left({\lambda }_{p\left({a}_{{t^{\prime}}\left(i\right)z\left(j\right)}\right)}^{\mathpzc{u}}\right)}^{2}\right)}^{{\partial }_{i}}\right)}^{{\Upsilon}_{j}}+\prod_{j=1}^{m}{\left(\prod_{i=1}^{1}{\left(1-{\left({\lambda }_{p\left({a}_{{t^{\prime}}\left(i\right)z\left(j\right)}\right)}^{\mathpzc{u}}\right)}^{2}\right)}^{{\partial }_{i}}\right)}^{{\Upsilon}_{j}}}}\end{array}\right], \\ \left[\begin{array}{c}\frac{\sqrt{2\prod_{j=1}^{m}{\left(\prod_{i=1}^{1}{\left({\left({\eta }_{p\left({a}_{{t^{\prime}}\left(i\right)z\left(j\right)}\right)}^{l}\right)}^{2}\right)}^{{\partial }_{i}}\right)}^{{\Upsilon}_{j}}}}{\sqrt{\prod_{j=1}^{m}{\left(\prod_{i=1}^{1}{\left(2-{\left({\eta }_{p\left({a}_{{t^{\prime}}\left(i\right)z\left(j\right)}\right)}^{l}\right)}^{2}\right)}^{{\partial }_{i}}\right)}^{{\Upsilon}_{j}}+\prod_{j=1}^{m}{\left(\prod_{i=1}^{1}{\left({\left({\eta }_{p\left({a}_{{t^{\prime}}\left(i\right)z\left(j\right)}\right)}^{l}\right)}^{2}\right)}^{{\partial }_{i}}\right)}^{{\Upsilon}_{j}}}},\\ \frac{\sqrt{2\prod_{j=1}^{m}{\left(\prod_{i=1}^{1}{\left({\left({\eta }_{p\left({a}_{{t^{\prime}}\left(i\right)z\left(j\right)}\right)}^{\mathpzc{u}}\right)}^{2}\right)}^{{\partial }_{i}}\right)}^{{\Upsilon}_{j}}}}{\sqrt{\prod_{j=1}^{m}{\left(\prod_{i=1}^{1}{\left(2-{\left({\eta }_{p\left({a}_{{t^{\prime}}\left(i\right)z\left(j\right)}\right)}^{\mathpzc{u}}\right)}^{2}\right)}^{{\partial }_{i}}\right)}^{{\Upsilon}_{j}}+\prod_{j=1}^{m}{\left(\prod_{i=1}^{1}{\left({\left({\eta }_{p\left({a}_{{t^{\prime}}\left(i\right)z\left(j\right)}\right)}^{\mathpzc{u}}\right)}^{2}\right)}^{{\partial }_{i}}\right)}^{{\Upsilon}_{j}}}}\end{array}\right]\end{array}\right)$$

For $m=1$, we get ${\Upsilon}_{j}=1$. So,

$$IVPFSEOWA\left({\Delta }_{{a}_{11}},{\Delta }_{{a}_{12}},\dots \dots {\Delta }_{{a}_{i1}}\right)$$

$$=\stackrel{n}{\underset{i=1}{{\oplus }_{\varepsilon }}}{\partial }_{i}{\Delta }_{p\left({a}_{{t^{\prime}}\left(i\right)z\left(1\right)}\right)}$$

$$=\left(\begin{array}{c}\left[\begin{array}{c}\frac{\sqrt{\prod_{i=1}^{n}{\left(1+{\left({\lambda }_{p\left({a}_{{t^{\prime}}\left(i\right)z\left(1\right)}\right)}^{l}\right)}^{2}\right)}^{{\partial }_{i}}-{\prod_{i=1}^{n}\left(1-{\left({\lambda }_{p\left({a}_{{t^{\prime}}\left(i\right)z\left(1\right)}\right)}^{l}\right)}^{2}\right)}^{{\partial }_{i}}}}{\sqrt{\prod_{i=1}^{n}{\left(1+{\left({\lambda }_{p\left({a}_{{t^{\prime}}\left(i\right)z\left(1\right)}\right)}^{l}\right)}^{2}\right)}^{{\partial }_{i}}+{\prod_{i=1}^{n}\left(1-{\left({\lambda }_{p\left({a}_{{t^{\prime}}\left(i\right)z\left(1\right)}\right)}^{l}\right)}^{2}\right)}^{{\partial }_{i}}}},\\ \frac{\sqrt{\prod_{i=1}^{n}{\left(1+{\left({\lambda }_{p\left({a}_{{t^{\prime}}\left(i\right)z\left(1\right)}\right)}^{\mathpzc{u}}\right)}^{2}\right)}^{{\partial }_{i}}-{\prod_{i=1}^{n}\left(1-{\left({\lambda }_{p\left({a}_{{t^{\prime}}\left(i\right)z\left(1\right)}\right)}^{\mathpzc{u}}\right)}^{2}\right)}^{{\partial }_{i}}}}{\sqrt{\prod_{i=1}^{n}{\left(1+{\left({\lambda }_{p\left({a}_{{t^{\prime}}\left(i\right)z\left(1\right)}\right)}^{\mathpzc{u}}\right)}^{2}\right)}^{{\partial }_{i}}+{\prod_{i=1}^{n}\left(1-{\left({\lambda }_{p\left({a}_{{t^{\prime}}\left(i\right)z\left(1\right)}\right)}^{\mathpzc{u}}\right)}^{2}\right)}^{{\partial }_{i}}}}\end{array}\right], \\ \left[\begin{array}{c}\frac{\sqrt{2\prod_{i=1}^{n}{\left({\left({\eta }_{p\left({a}_{{t^{\prime}}\left(i\right)z\left(1\right)}\right)}^{l}\right)}^{2}\right)}^{{\partial }_{i}}}}{\sqrt{\prod_{i=1}^{n}{\left(2-{\left({\eta }_{p\left({a}_{{t^{\prime}}\left(i\right)z\left(1\right)}\right)}^{l}\right)}^{2}\right)}^{{\partial }_{i}}+\prod_{j=1}^{m}{\left({\left({\eta }_{p\left({a}_{{t^{\prime}}\left(i\right)z\left(1\right)}\right)}^{l}\right)}^{2}\right)}^{{\partial }_{i}}}},\\ \frac{\sqrt{2\prod_{i=1}^{n}{\left({\left({\eta }_{p\left({a}_{{t^{\prime}}\left(i\right)z\left(1\right)}\right)}^{\mathpzc{u}}\right)}^{2}\right)}^{{\partial }_{i}}}}{\sqrt{\prod_{i=1}^{n}{\left(2-{\left({\eta }_{p\left({a}_{{t^{\prime}}\left(i\right)z\left(1\right)}\right)}^{\mathpzc{u}}\right)}^{2}\right)}^{{\partial }_{i}}+\prod_{j=1}^{m}{\left({\left({\eta }_{p\left({a}_{{t^{\prime}}\left(i\right)z\left(1\right)}\right)}^{\mathpzc{u}}\right)}^{2}\right)}^{{\partial }_{i}}}}\end{array}\right]\end{array}\right)$$

$$=\left(\begin{array}{c}\left[\begin{array}{c}\frac{\sqrt{\prod_{j=1}^{1}{\left(\prod_{i=1}^{n}{\left(1+{\left({\lambda }_{p\left({a}_{{t^{\prime}}\left(i\right)z\left(j\right)}\right)}^{l}\right)}^{2}\right)}^{{\partial }_{i}}\right)}^{{\Upsilon}_{j}}-\prod_{j=1}^{1}{\left(\prod_{i=1}^{n}{\left(1-{\left({\lambda }_{p\left({a}_{{t^{\prime}}\left(i\right)z\left(j\right)}\right)}^{l}\right)}^{2}\right)}^{{\partial }_{i}}\right)}^{{\Upsilon}_{j}}}}{\sqrt{\prod_{j=1}^{1}{\left(\prod_{i=1}^{n}{\left(1+{\left({\lambda }_{p\left({a}_{{t^{\prime}}\left(i\right)z\left(j\right)}\right)}^{l}\right)}^{2}\right)}^{{\partial }_{i}}\right)}^{{\Upsilon}_{j}}+\prod_{j=1}^{1}{\left(\prod_{i=1}^{n}{\left(1-{\left({\lambda }_{p\left({a}_{{t^{\prime}}\left(i\right)z\left(j\right)}\right)}^{l}\right)}^{2}\right)}^{{\partial }_{i}}\right)}^{{\Upsilon}_{j}}}},\\ \frac{\sqrt{\prod_{j=1}^{1}{\left(\prod_{i=1}^{n}{\left(1+{\left({\lambda }_{p\left({a}_{{t^{\prime}}\left(i\right)z\left(j\right)}\right)}^{\mathpzc{u}}\right)}^{2}\right)}^{{\partial }_{i}}\right)}^{{\Upsilon}_{j}}-\prod_{j=1}^{1}{\left(\prod_{i=1}^{n}{\left(1-{\left({\lambda }_{p\left({a}_{{t^{\prime}}\left(i\right)z\left(j\right)}\right)}^{\mathpzc{u}}\right)}^{2}\right)}^{{\partial }_{i}}\right)}^{{\Upsilon}_{j}}}}{\sqrt{\prod_{j=1}^{1}{\left(\prod_{i=1}^{n}{\left(1+{\left({\lambda }_{p\left({a}_{{t^{\prime}}\left(i\right)z\left(j\right)}\right)}^{\mathpzc{u}}\right)}^{2}\right)}^{{\partial }_{i}}\right)}^{{\Upsilon}_{j}}+\prod_{j=1}^{1}{\left(\prod_{i=1}^{n}{\left(1-{\left({\lambda }_{p\left({a}_{{t^{\prime}}\left(i\right)z\left(j\right)}\right)}^{\mathpzc{u}}\right)}^{2}\right)}^{{\partial }_{i}}\right)}^{{\Upsilon}_{j}}}}\end{array}\right], \\ \left[\begin{array}{c}\frac{\sqrt{2\prod_{j=1}^{1}{\left(\prod_{i=1}^{n}{\left({\left({\eta }_{p\left({a}_{{t^{\prime}}\left(i\right)z\left(j\right)}\right)}^{l}\right)}^{2}\right)}^{{\partial }_{i}}\right)}^{{\Upsilon}_{j}}}}{\sqrt{\prod_{j=1}^{1}{\left(\prod_{i=1}^{n}{\left(2-{\left({\eta }_{p\left({a}_{{t^{\prime}}\left(i\right)z\left(j\right)}\right)}^{l}\right)}^{2}\right)}^{{\partial }_{i}}\right)}^{{\Upsilon}_{j}}+\prod_{j=1}^{1}{\left(\prod_{i=1}^{n}{\left({\left({\eta }_{p\left({a}_{{t^{\prime}}\left(i\right)z\left(j\right)}\right)}^{l}\right)}^{2}\right)}^{{\partial }_{i}}\right)}^{{\Upsilon}_{j}}}},\\ \frac{\sqrt{2\prod_{j=1}^{1}{\left(\prod_{i=1}^{n}{\left({\left({\eta }_{p\left({a}_{{t^{\prime}}\left(i\right)z\left(j\right)}\right)}^{\mathpzc{u}}\right)}^{2}\right)}^{{\partial }_{i}}\right)}^{{\Upsilon}_{j}}}}{\sqrt{\prod_{j=1}^{1}{\left(\prod_{i=1}^{n}{\left(2-{\left({\eta }_{p\left({a}_{{t^{\prime}}\left(i\right)z\left(j\right)}\right)}^{\mathpzc{u}}\right)}^{2}\right)}^{{\partial }_{i}}\right)}^{{\Upsilon}_{j}}+\prod_{j=1}^{1}{\left(\prod_{i=1}^{n}{\left({\left({\eta }_{p\left({a}_{{t^{\prime}}\left(i\right)z\left(j\right)}\right)}^{\mathpzc{u}}\right)}^{2}\right)}^{{\partial }_{i}}\right)}^{{\Upsilon}_{j}}}}\end{array}\right]\end{array}\right)$$

This shows that for $n=1$ and $m=1$ Eq. 3 is satisfied.

For $$n=2$$ and $$m=1$$

$$IVPFSEOWA\left({\Delta }_{{a}_{11}}, {\Delta }_{{a}_{21}}\right)$$

$$={\Upsilon}_{1}\left({\partial }_{1}{\Delta }_{{a}_{11}}\right){\oplus }_{\varepsilon }{\Upsilon}_{1}\left({\partial }_{2}{\Delta }_{{a}_{21}}\right)$$

$${\Upsilon}_{1}\left({\partial }_{1}{\Delta }_{{a}_{11}}\right)$$

$$=\left(\begin{array}{c}\left[\begin{array}{c}\frac{\sqrt{{\left({\left(1+{\left({\lambda }_{{a}_{11}}^{l}\right)}^{2}\right)}^{{\partial }_{1}}\right)}^{{\Upsilon}_{1}}-{\left({\left(1-{\left({\lambda }_{{a}_{11}}^{l}\right)}^{2}\right)}^{{\partial }_{1}}\right)}^{{\Upsilon}_{1}}}}{\sqrt{{\left({\left(1+{\left({\lambda }_{{a}_{11}}^{l}\right)}^{2}\right)}^{{\partial }_{1}}\right)}^{{\Upsilon}_{1}}+{\left({\left(1-{\left({\lambda }_{{a}_{11}}^{l}\right)}^{2}\right)}^{{\partial }_{1}}\right)}^{{\Upsilon}_{1}}}},\frac{\sqrt{{\left({\left(1+{\left({\lambda }_{{a}_{11}}^{\mathpzc{u}}\right)}^{2}\right)}^{{\partial }_{1}}\right)}^{{\Upsilon}_{1}}-{\left({\left(1-{\left({\lambda }_{{a}_{11}}^{\mathpzc{u}}\right)}^{2}\right)}^{{\partial }_{1}}\right)}^{{\Upsilon}_{1}}}}{\sqrt{{\left({\left(1+{\left({\lambda }_{{a}_{11}}^{\mathpzc{u}}\right)}^{2}\right)}^{{\partial }_{1}}\right)}^{{\Upsilon}_{1}}+{\left({\left(1-{\left({\lambda }_{{a}_{11}}^{\mathpzc{u}}\right)}^{2}\right)}^{{\partial }_{1}}\right)}^{{\Upsilon}_{1}}}}\end{array}\right],\\ \left[\begin{array}{c}\frac{\sqrt{2{\left({\left({\left({\eta }_{{a}_{11}}^{l}\right)}^{2}\right)}^{{\partial }_{1}}\right)}^{{\Upsilon}_{1}}}}{\sqrt{{\left({\left(2-{\left({\eta }_{{a}_{11}}^{l}\right)}^{2}\right)}^{{\partial }_{1}}\right)}^{{\Upsilon}_{1}}+{\left({\left({\left({\eta }_{{a}_{11}}^{l}\right)}^{2}\right)}^{{\partial }_{1}}\right)}^{{\Upsilon}_{1}}}},\frac{\sqrt{2{\left({\left({\left({\eta }_{{a}_{11}}^{\mathpzc{u}}\right)}^{2}\right)}^{{\partial }_{1}}\right)}^{{\Upsilon}_{1}}}}{\sqrt{{\left({\left(2-{\left({\eta }_{{a}_{11}}^{\mathpzc{u}}\right)}^{2}\right)}^{{\partial }_{1}}\right)}^{{\Upsilon}_{1}}+{\left({\left({\left({\eta }_{{a}_{11}}^{\mathpzc{u}}\right)}^{2}\right)}^{{\partial }_{1}}\right)}^{{\Upsilon}_{1}}}}\end{array}\right]\end{array}\right)$$

$${\Upsilon}_{1}\left({\partial }_{2}{\Delta }_{{a}_{21}}\right)$$

$$=\left(\begin{array}{c}\left[\begin{array}{c}\frac{\sqrt{{\left({\left(1+{\left({\lambda }_{{a}_{21}}^{l}\right)}^{2}\right)}^{{\partial }_{2}}\right)}^{{\Upsilon}_{1}}-{\left({\left(1-{\left({\lambda }_{{a}_{21}}^{l}\right)}^{2}\right)}^{{\partial }_{2}}\right)}^{{\Upsilon}_{1}}}}{\sqrt{{\left({\left(1+{\left({\lambda }_{{a}_{21}}^{l}\right)}^{2}\right)}^{{\partial }_{2}}\right)}^{{\Upsilon}_{1}}+{\left({\left(1-{\left({\lambda }_{{a}_{21}}^{l}\right)}^{2}\right)}^{{\partial }_{2}}\right)}^{{\Upsilon}_{1}}}},\frac{\sqrt{{\left({\left(1+{\left({\lambda }_{{a}_{21}}^{\mathpzc{u}}\right)}^{2}\right)}^{{\partial }_{2}}\right)}^{{\Upsilon}_{1}}-{\left({\left(1-{\left({\lambda }_{{a}_{21}}^{\mathpzc{u}}\right)}^{2}\right)}^{{\partial }_{2}}\right)}^{{\Upsilon}_{1}}}}{\sqrt{{\left({\left(1+{\left({\lambda }_{{a}_{21}}^{\mathpzc{u}}\right)}^{2}\right)}^{{\partial }_{2}}\right)}^{{\Upsilon}_{1}}+{\left({\left(1-{\left({\lambda }_{{a}_{21}}^{\mathpzc{u}}\right)}^{2}\right)}^{{\partial }_{2}}\right)}^{{\Upsilon}_{1}}}}\end{array}\right],\\ \left[\begin{array}{c}\frac{\sqrt{2{\left({\left({\left({\eta }_{{a}_{21}}^{l}\right)}^{2}\right)}^{{\partial }_{2}}\right)}^{{\Upsilon}_{1}}}}{\sqrt{{\left({\left(2-{\left({\eta }_{{a}_{21}}^{l}\right)}^{2}\right)}^{{\partial }_{2}}\right)}^{{\Upsilon}_{1}}+{\left({\left({\left({\eta }_{{a}_{21}}^{l}\right)}^{2}\right)}^{{\partial }_{2}}\right)}^{{\Upsilon}_{1}}}},\frac{\sqrt{2{\left({\left({\left({\eta }_{{a}_{21}}^{\mathpzc{u}}\right)}^{2}\right)}^{{\partial }_{2}}\right)}^{{\Upsilon}_{1}}}}{\sqrt{{\left({\left(2-{\left({\eta }_{{a}_{21}}^{\mathpzc{u}}\right)}^{2}\right)}^{{\partial }_{2}}\right)}^{{\Upsilon}_{1}}+{\left({\left({\left({\eta }_{{a}_{21}}^{\mathpzc{u}}\right)}^{2}\right)}^{{\partial }_{2}}\right)}^{{\Upsilon}_{1}}}}\end{array}\right]\end{array}\right)$$

let.

$${\rho }_{1}={\left({\left(1+{\left({\lambda }_{{a}_{11}}^{l}\right)}^{2}\right)}^{{\partial }_{1}}\right)}^{{\Upsilon}_{1}}; {\varrho }_{1}={\left({\left(1-{\left({\lambda }_{{a}_{11}}^{l}\right)}^{2}\right)}^{{\partial }_{1}}\right)}^{{\Upsilon}_{1}};$$

$${\Gamma }_{1}={\left({\left(1+{\left({\lambda }_{{a}_{11}}^{\mathpzc{u}}\right)}^{2}\right)}^{{\partial }_{1}}\right)}^{{\Upsilon}_{1}}; {\xi }_{1}={\left({\left(1-{\left({\lambda }_{{a}_{11}}^{\mathpzc{u}}\right)}^{2}\right)}^{{\partial }_{1}}\right)}^{{\Upsilon}_{1}};$$

$${\rho }_{2}={\left({\left(1+{\left({\lambda }_{{a}_{21}}^{l}\right)}^{2}\right)}^{{\partial }_{2}}\right)}^{{\Upsilon}_{1}} {\varrho }_{2}={\left({\left(1-{\left({\lambda }_{{a}_{21}}^{l}\right)}^{2}\right)}^{{\partial }_{2}}\right)}^{{\Upsilon}_{1}}$$

$${\Gamma }_{2}={\left({\left(1+{\left({\lambda }_{{a}_{21}}^{\mathpzc{u}}\right)}^{2}\right)}^{{\partial }_{2}}\right)}^{{\Upsilon}_{1}}; {\xi }_{2}={\left({\left(1-{\left({\lambda }_{{a}_{21}}^{\mathpzc{u}}\right)}^{2}\right)}^{{\partial }_{2}}\right)}^{{\Upsilon}_{1}};$$

$$\widehat{{\rho }_{1}}={\left({\left({\left({\eta }_{{a}_{11}}^{l}\right)}^{2}\right)}^{{\partial }_{1}}\right)}^{{\Upsilon}_{1}}; \widehat{{\varrho }_{1}}={\left({\left(2-{\left({\eta }_{{a}_{11}}^{l}\right)}^{2}\right)}^{{\partial }_{1}}\right)}^{{\Upsilon}_{1}};$$

$$\widehat{{\Gamma }_{1}}={\left({\left({\left({\eta }_{{a}_{11}}^{\mathpzc{u}}\right)}^{2}\right)}^{{\partial }_{1}}\right)}^{{\Upsilon}_{1}}; \widehat{{\xi }_{1}}={\left({\left(2-{\left({\eta }_{{a}_{11}}^{\mathpzc{u}}\right)}^{2}\right)}^{{\partial }_{1}}\right)}^{{\Upsilon}_{1}};$$

$$\widehat{{\rho }_{2}}={\left({\left({\left({\eta }_{{a}_{21}}^{l}\right)}^{2}\right)}^{{\partial }_{2}}\right)}^{{\Upsilon}_{1}}; \widehat{{\varrho }_{2}}={\left({\left(2-{\left({\eta }_{{a}_{21}}^{l}\right)}^{2}\right)}^{{\partial }_{2}}\right)}^{{\Upsilon}_{1}};$$

$$\widehat{{\Gamma }_{2}}={\left({\left({\left({\eta }_{{a}_{21}}^{\mathpzc{u}}\right)}^{2}\right)}^{{\partial }_{2}}\right)}^{{\Upsilon}_{1}}$$; and $$\widehat{{\xi }_{2}}={\left({\left(2-{\left({\eta }_{{a}_{21}}^{\mathpzc{u}}\right)}^{2}\right)}^{{\partial }_{2}}\right)}^{{\Upsilon}_{1}}$$.

Then,

$${\Upsilon}_{1}\left({\partial }_{1}{\Delta }_{{a}_{11}}\right)=\left(\left[\begin{array}{c}\frac{\sqrt{{\rho }_{1}-{\varrho }_{1}}}{\sqrt{{\rho }_{1}+{\varrho }_{1}}},\frac{\sqrt{\widehat{{\Gamma }_{1}}-\widehat{{\xi }_{1}}}}{\sqrt{\widehat{{\Gamma }_{1}}+\widehat{{\xi }_{1}}}}\end{array}\right], \left[\begin{array}{c}\frac{\sqrt{2\widehat{{\rho }_{1}}}}{\sqrt{\widehat{{\rho }_{1}}+\widehat{{\varrho }_{1}}}},\frac{\sqrt{2\widehat{{\varrho }_{1}}}}{\sqrt{\widehat{{\rho }_{1}}+\widehat{{\varrho }_{1}}}}\end{array}\right]\right)$$

and $${\Upsilon}_{1}\left({\partial }_{2}{\Delta }_{{a}_{21}}\right)=\left(\left[\begin{array}{c}\frac{\sqrt{{\rho }_{2}-{\varrho }_{2}}}{\sqrt{{\rho }_{2}+{\varrho }_{2}}},\frac{\sqrt{\widehat{{\Gamma }_{2}}-\widehat{{\xi }_{2}}}}{\sqrt{\widehat{{\Gamma }_{2}}+\widehat{{\xi }_{2}}}}\end{array}\right], \left[\begin{array}{c}\frac{\sqrt{2\widehat{{\rho }_{2}}}}{\sqrt{\widehat{{\rho }_{2}}+\widehat{{\varrho }_{2}}}},\frac{\sqrt{2\widehat{{\varrho }_{2}}}}{\sqrt{\widehat{{\rho }_{2}}+\widehat{{\varrho }_{2}}}}\end{array}\right]\right)$$

So,

$$IVPFSEOWA\left({\Delta }_{{a}_{11}}, {\Delta }_{{a}_{21}}\right)$$

$$={\Upsilon}_{1}\left({\partial }_{1}{\Delta }_{{a}_{11}}\right){\oplus }_{\varepsilon }{\Upsilon}_{1}\left({\partial }_{2}{\Delta }_{{a}_{21}}\right)$$

$$=\left(\left[\begin{array}{c}\frac{\sqrt{{\rho }_{1}{\rho }_{2}-{\varrho }_{1}{\varrho }_{2}}}{\sqrt{{\rho }_{1}{\rho }_{2}+{\varrho }_{1}{\varrho }_{2}}},\end{array}\frac{\sqrt{\widehat{{\Gamma }_{1}}\widehat{{\Gamma }_{1}}-\widehat{{\xi }_{1}}\widehat{{\xi }_{2}}}}{\sqrt{\widehat{{\Gamma }_{1}}\widehat{{\Gamma }_{2}}+\widehat{{\xi }_{1}}\widehat{{\xi }_{2}}}}\right]\right),\left(\left[\begin{array}{c}\frac{\sqrt{2\widehat{{\rho }_{1}}\widehat{{\rho }_{2}}}}{\sqrt{\widehat{{\rho }_{1}}\widehat{{\rho }_{2}}+\widehat{{\varrho }_{1}}\widehat{{\varrho }_{2}}}},\frac{\sqrt{2\widehat{{\Gamma }_{1}}\widehat{{\Gamma }_{2}}}}{\sqrt{\widehat{{\Gamma }_{1}}\widehat{{\Gamma }_{2}}+\widehat{{\xi }_{1}}\widehat{{\xi }_{2}}}}\end{array}\right]\right)$$

$$=\left(\begin{array}{c}\left[\begin{array}{c}\frac{\sqrt{\prod_{j=1}^{1}{\left(\prod_{i=1}^{2}{\left(1+{\left({\lambda }_{p\left({a}_{{t^{\prime}}\left(i\right)z\left(j\right)}\right)}^{l}\right)}^{2}\right)}^{{\partial }_{i}}\right)}^{{\Upsilon}_{j}}-\prod_{j=1}^{1}{\left(\prod_{i=1}^{2}{\left(1-{\left({\lambda }_{p\left({a}_{{t^{\prime}}\left(i\right)z\left(j\right)}\right)}^{l}\right)}^{2}\right)}^{{\partial }_{i}}\right)}^{{\Upsilon}_{j}}}}{\sqrt{\prod_{j=1}^{1}{\left(\prod_{i=1}^{2}{\left(1+{\left({\lambda }_{p\left({a}_{{t^{\prime}}\left(i\right)z\left(j\right)}\right)}^{l}\right)}^{2}\right)}^{{\partial }_{i}}\right)}^{{\Upsilon}_{j}}+\prod_{j=1}^{1}{\left(\prod_{i=1}^{2}{\left(1-{\left({\lambda }_{p\left({a}_{{t^{\prime}}\left(i\right)z\left(j\right)}\right)}^{l}\right)}^{2}\right)}^{{\partial }_{i}}\right)}^{{\Upsilon}_{j}}}},\\ \frac{\sqrt{\prod_{j=1}^{1}{\left(\prod_{i=1}^{2}{\left(1+{\left({\lambda }_{p\left({a}_{{t^{\prime}}\left(i\right)z\left(j\right)}\right)}^{\mathpzc{u}}\right)}^{2}\right)}^{{\partial }_{i}}\right)}^{{\Upsilon}_{j}}-\prod_{j=1}^{1}{\left(\prod_{i=1}^{2}{\left(1-{\left({\lambda }_{p\left({a}_{{t^{\prime}}\left(i\right)z\left(j\right)}\right)}^{\mathpzc{u}}\right)}^{2}\right)}^{{\partial }_{i}}\right)}^{{\Upsilon}_{j}}}}{\sqrt{\prod_{j=1}^{1}{\left(\prod_{i=1}^{2}{\left(1+{\left({\lambda }_{p\left({a}_{{t^{\prime}}\left(i\right)z\left(j\right)}\right)}^{\mathpzc{u}}\right)}^{2}\right)}^{{\partial }_{i}}\right)}^{{\Upsilon}_{j}}+\prod_{j=1}^{1}{\left(\prod_{i=1}^{2}{\left(1-{\left({\lambda }_{p\left({a}_{{t^{\prime}}\left(i\right)z\left(j\right)}\right)}^{\mathpzc{u}}\right)}^{2}\right)}^{{\partial }_{i}}\right)}^{{\Upsilon}_{j}}}}\end{array}\right],\\ \left[\begin{array}{c}\frac{\sqrt{2\prod_{j=1}^{1}{\left(\prod_{i=1}^{2}{\left({\left({\eta }_{p\left({a}_{{t^{\prime}}\left(i\right)z\left(j\right)}\right)}^{l}\right)}^{2}\right)}^{{\partial }_{i}}\right)}^{{\Upsilon}_{j}}}}{\sqrt{\prod_{j=1}^{1}{\left(\prod_{i=1}^{2}{\left(2-{\left({\eta }_{p\left({a}_{{t^{\prime}}\left(i\right)z\left(j\right)}\right)}^{l}\right)}^{2}\right)}^{{\partial }_{i}}\right)}^{{\Upsilon}_{j}}+\prod_{j=1}^{1}{\left(\prod_{i=1}^{2}{\left({\left({\eta }_{p\left({a}_{{t^{\prime}}\left(i\right)z\left(j\right)}\right)}^{l}\right)}^{2}\right)}^{{\partial }_{i}}\right)}^{{\Upsilon}_{j}}}},\\ \frac{\sqrt{2\prod_{j=1}^{1}{\left(\prod_{i=1}^{2}{\left({\left({\eta }_{p\left({a}_{{t^{\prime}}\left(i\right)z\left(j\right)}\right)}^{\mathpzc{u}}\right)}^{2}\right)}^{{\partial }_{i}}\right)}^{{\Upsilon}_{j}}}}{\sqrt{\prod_{j=1}^{1}{\left(\prod_{i=1}^{2}{\left(2-{\left({\eta }_{p\left({a}_{{t^{\prime}}\left(i\right)z\left(j\right)}\right)}^{\mathpzc{u}}\right)}^{2}\right)}^{{\partial }_{i}}\right)}^{{\Upsilon}_{j}}+\prod_{j=1}^{1}{\left(\prod_{i=1}^{2}{\left({\left({\eta }_{p\left({a}_{{t^{\prime}}\left(i\right)z\left(j\right)}\right)}^{\mathpzc{u}}\right)}^{2}\right)}^{{\partial }_{i}}\right)}^{{\Upsilon}_{j}}}}\end{array}\right]\end{array}\right)$$

It is proved that Eq. 3 is held for $n=2$ and $m=1$. Now, we will assume that it is true for $n=k$ and $m={\ell}$. Then,

$$IVPFSEOWA\left({\Delta }_{{a}_{11}},{\Delta }_{{a}_{12}},\dots \dots {\Delta }_{{a}_{k{\ell}}}\right)$$

$$=\left(\begin{array}{c}\left[\begin{array}{c}\frac{\sqrt{\prod_{j=1}^{{\ell}}{\left(\prod_{i=1}^{k}{\left(1+{\left({\lambda }_{p\left({a}_{{t^{\prime}}\left(i\right)z\left(j\right)}\right)}^{l}\right)}^{2}\right)}^{{\partial }_{i}}\right)}^{{\Upsilon}_{j}}-\prod_{j=1}^{{\ell}}{\left(\prod_{i=1}^{k}{\left(1-{\left({\lambda }_{p\left({a}_{{t^{\prime}}\left(i\right)z\left(j\right)}\right)}^{l}\right)}^{2}\right)}^{{\partial }_{i}}\right)}^{{\Upsilon}_{j}}}}{\sqrt{\prod_{j=1}^{{\ell}}{\left(\prod_{i=1}^{k}{\left(1+{\left({\lambda }_{p\left({a}_{{t^{\prime}}\left(i\right)z\left(j\right)}\right)}^{l}\right)}^{2}\right)}^{{\partial }_{i}}\right)}^{{\Upsilon}_{j}}+\prod_{j=1}^{{\ell}}{\left(\prod_{i=1}^{k}{\left(1-{\left({\lambda }_{p\left({a}_{{t^{\prime}}\left(i\right)z\left(j\right)}\right)}^{l}\right)}^{2}\right)}^{{\partial }_{i}}\right)}^{{\Upsilon}_{j}}}},\\ \frac{\sqrt{\prod_{j=1}^{{\ell}}{\left(\prod_{i=1}^{k}{\left(1+{\left({\lambda }_{p\left({a}_{{t^{\prime}}\left(i\right)z\left(j\right)}\right)}^{\mathpzc{u}}\right)}^{2}\right)}^{{\partial }_{i}}\right)}^{{\Upsilon}_{j}}-\prod_{j=1}^{{\ell}}{\left(\prod_{i=1}^{k}{\left(1-{\left({\lambda }_{p\left({a}_{{t^{\prime}}\left(i\right)z\left(j\right)}\right)}^{\mathpzc{u}}\right)}^{2}\right)}^{{\partial }_{i}}\right)}^{{\Upsilon}_{j}}}}{\sqrt{\prod_{j=1}^{{\ell}}{\left(\prod_{i=1}^{k}{\left(1+{\left({\lambda }_{p\left({a}_{{t^{\prime}}\left(i\right)z\left(j\right)}\right)}^{\mathpzc{u}}\right)}^{2}\right)}^{{\partial }_{i}}\right)}^{{\Upsilon}_{j}}+\prod_{j=1}^{{\ell}}{\left(\prod_{i=1}^{k}{\left(1-{\left({\lambda }_{p\left({a}_{{t^{\prime}}\left(i\right)z\left(j\right)}\right)}^{\mathpzc{u}}\right)}^{2}\right)}^{{\partial }_{i}}\right)}^{{\Upsilon}_{j}}}}\end{array}\right],\\ \left[\begin{array}{c}\frac{\sqrt{2\prod_{j=1}^{{\ell}}{\left(\prod_{i=1}^{k}{\left({\left({\eta }_{p\left({a}_{{t^{\prime}}\left(i\right)z\left(j\right)}\right)}^{l}\right)}^{2}\right)}^{{\partial }_{i}}\right)}^{{\Upsilon}_{j}}}}{\sqrt{\prod_{j=1}^{{\ell}}{\left(\prod_{i=1}^{k}{\left(2-{\left({\eta }_{p\left({a}_{{t^{\prime}}\left(i\right)z\left(j\right)}\right)}^{l}\right)}^{2}\right)}^{{\partial }_{i}}\right)}^{{\Upsilon}_{j}}+\prod_{j=1}^{{\ell}}{\left(\prod_{i=1}^{k}{\left({\left({\eta }_{p\left({a}_{{t^{\prime}}\left(i\right)z\left(j\right)}\right)}^{l}\right)}^{2}\right)}^{{\partial }_{i}}\right)}^{{\Upsilon}_{j}}}},\\ \frac{\sqrt{2\prod_{j=1}^{{\ell}}{\left(\prod_{i=1}^{k}{\left({\left({\eta }_{p\left({a}_{{t^{\prime}}\left(i\right)z\left(j\right)}\right)}^{\mathpzc{u}}\right)}^{2}\right)}^{{\partial }_{i}}\right)}^{{\Upsilon}_{j}}}}{\sqrt{\prod_{j=1}^{{\ell}}{\left(\prod_{i=1}^{k}{\left(2-{\left({\eta }_{p\left({a}_{{t^{\prime}}\left(i\right)z\left(j\right)}\right)}^{\mathpzc{u}}\right)}^{2}\right)}^{{\partial }_{i}}\right)}^{{\Upsilon}_{j}}+\prod_{j=1}^{{\ell}}{\left(\prod_{i=1}^{k}{\left({\left({\eta }_{p\left({a}_{{t^{\prime}}\left(i\right)z\left(j\right)}\right)}^{\mathpzc{u}}\right)}^{2}\right)}^{{\partial }_{i}}\right)}^{{\Upsilon}_{j}}}}\end{array}\right]\end{array}\right)$$

for $$n=k+1$$ and $$m={\ell}+1$$.

$$IVPFSEOWA\left({\Delta }_{{a}_{11}},{\Delta }_{{a}_{12}},\dots \dots {\Delta }_{{a}_{\left(k+1\right)\left({\ell}+1\right)}}\right)$$

$$=IVPFSEOWA\left({\Delta }_{{a}_{11}},{\Delta }_{{a}_{12}},\dots \dots {\Delta }_{{a}_{\left(k+1\right)\left({\ell}+1\right)}}\right){\oplus }_{\varepsilon }{\Upsilon}_{\left({\ell}+1\right)}\left({\partial }_{\left(k+1\right)}{\Delta }_{{a}_{\left(k+1\right)\left({\ell}+1\right)}}\right)$$

$${\Upsilon}_{\left(l+1\right)}\left({\partial }_{\left(k+1\right)}{\Delta }_{{a}_{\left(k+1\right)\left({\ell}+1\right)}}\right)$$

$$=\left(\begin{array}{c}\left[\begin{array}{c}\frac{\sqrt{{\left({\left(1+{\left({\lambda }_{{a}_{\left(k+1\right)\left({\ell}+1\right)}}^{l}\right)}^{2}\right)}^{{\partial }_{k+1}}\right)}^{{\Upsilon}_{{\ell}+1}}-{\left({\left(1-{\left({\lambda }_{{a}_{\left(k+1\right)\left({\ell}+1\right)}}^{l}\right)}^{2}\right)}^{{\partial }_{k+1}}\right)}^{{\Upsilon}_{{\ell}+1}}}}{\sqrt{{\left({\left(1+{\left({\lambda }_{{a}_{\left(k+1\right)\left({\ell}+1\right)}}^{l}\right)}^{2}\right)}^{{\partial }_{k+1}}\right)}^{{\Upsilon}_{{\ell}+1}}+{\left({\left(1-{\left({\lambda }_{{a}_{\left(k+1\right)\left({\ell}+1\right)}}^{l}\right)}^{2}\right)}^{{\partial }_{k+1}}\right)}^{{\Upsilon}_{{\ell}+1}}}},\\ \frac{\sqrt{{\left({\left(1+{\left({\lambda }_{{a}_{\left(k+1\right)\left({\ell}+1\right)}}^{\mathpzc{u}}\right)}^{2}\right)}^{{\partial }_{k+1}}\right)}^{{\Upsilon}_{{\ell}+1}}-{\left({\left(1-{\left({\lambda }_{{a}_{\left(k+1\right)\left({\ell}+1\right)}}^{\mathpzc{u}}\right)}^{2}\right)}^{{\partial }_{k+1}}\right)}^{{\Upsilon}_{{\ell}+1}}}}{\sqrt{{\left({\left(1+{\left({\lambda }_{{a}_{\left(k+1\right)\left({\ell}+1\right)}}^{\mathpzc{u}}\right)}^{2}\right)}^{{\partial }_{k+1}}\right)}^{{\Upsilon}_{{\ell}+1}}+{\left({\left(1-{\left({\lambda }_{{a}_{\left(k+1\right)\left({\ell}+1\right)}}^{\mathpzc{u}}\right)}^{2}\right)}^{{\partial }_{k+1}}\right)}^{{\Upsilon}_{{\ell}+1}}}}\end{array}\right]\\ ,\left[\begin{array}{c}\frac{\sqrt{2{\left({\left({\left({\eta }_{{a}_{\left(k+1\right)\left({\ell}+1\right)}}^{l}\right)}^{2}\right)}^{{\partial }_{k+1}}\right)}^{{\Upsilon}_{{\ell}+1}}}}{\sqrt{{\left({\left(2-{\left({\eta }_{{a}_{\left(k+1\right)\left({\ell}+1\right)}}^{l}\right)}^{2}\right)}^{{\partial }_{k+1}}\right)}^{{\Upsilon}_{{\ell}+1}}+{\left({\left({\left({\eta }_{{a}_{\left(k+1\right)\left({\ell}+1\right)}}^{l}\right)}^{2}\right)}^{{\partial }_{k+1}}\right)}^{{\Upsilon}_{{\ell}+1}}}},\\ \frac{\sqrt{2{\left({\left({\left({\eta }_{{a}_{\left(k+1\right)\left({\ell}+1\right)}}^{\mathpzc{u}}\right)}^{2}\right)}^{{\partial }_{k+1}}\right)}^{{\Upsilon}_{{\ell}+1}}}}{\sqrt{{\left({\left(2-{\left({\eta }_{{a}_{\left(k+1\right)\left({\ell}+1\right)}}^{\mathpzc{u}}\right)}^{2}\right)}^{{\partial }_{k+1}}\right)}^{{\Upsilon}_{{\ell}+1}}+{\left({\left({\left({\eta }_{{a}_{\left(k+1\right)\left({\ell}+1\right)}}^{\mathpzc{u}}\right)}^{2}\right)}^{{\partial }_{k+1}}\right)}^{{\Upsilon}_{{\ell}+1}}}}\end{array}\right]\end{array}\right)$$

For Simplicity.

$${\rho }_{1}=\prod_{j=1}^{{\ell}}{\left(\prod_{i=1}^{k}{\left(1+{\left({\lambda }_{\mathrm{p}\left({a}_{{{t^{\prime}}}\left(i\right)\mathrm{z}\left(j\right)}\right)}^{l}\right)}^{2}\right)}^{{\partial }_{i}}\right)}^{{\Upsilon}_{j}};$$

$${\varrho }_{1}=\prod_{j=1}^{{\ell}}{\left(\prod_{i=1}^{k}{\left(1-{\left({\lambda }_{\mathrm{p}\left({a}_{{{t^{\prime}}}\left(i\right)\mathrm{z}\left(j\right)}\right)}^{l}\right)}^{2}\right)}^{{\partial }_{i}}\right)}^{{\Upsilon}_{j}};$$

$${\Gamma }_{1}=\prod_{j=1}^{{\ell}}{\left(\prod_{i=1}^{k}{\left(1+{\left({\lambda }_{\mathrm{p}\left({a}_{{{t^{\prime}}}\left(i\right)\mathrm{z}\left(j\right)}\right)}^{l}\right)}^{2}\right)}^{{\partial }_{i}}\right)}^{{\Upsilon}_{j}};$$

$${\xi }_{1}=\prod_{j=1}^{{\ell}}{\left(\prod_{i=1}^{k}{\left(1-{\left({\lambda }_{\mathrm{p}\left({a}_{{{t^{\prime}}}\left(i\right)\mathrm{z}\left(j\right)}\right)}^{l}\right)}^{2}\right)}^{{\partial }_{i}}\right)}^{{\Upsilon}_{j}};$$

$${\rho }_{2}={\left({\left(1+{\left({\lambda }_{{a}_{\left(k+1\right)\left({\ell}+1\right)}}^{l}\right)}^{2}\right)}^{{\partial }_{k+1}}\right)}^{{\Upsilon}_{{\ell}+1}};$$

$${\varrho }_{2}={\left({\left(1-{\left({\lambda }_{{a}_{\left(k+1\right)\left({\ell}+1\right)}}^{l}\right)}^{2}\right)}^{{\partial }_{k+1}}\right)}^{{\Upsilon}_{{\ell}+1}};$$

$${\Gamma }_{2}={\left(\left({\left(1+{\left({\lambda }_{{a}_{\left(k+1\right)\left({\ell}+1\right)}}^{\mathpzc{u}}\right)}^{2}\right)}^{{\partial }_{k+1}}\right)\right)}^{{\Upsilon}_{{\ell}+1}};$$

$${\xi }_{2}={\left(\left({\left(1-{\left({\lambda }_{{a}_{\left(k+1\right)\left({\ell}+1\right)}}^{\mathpzc{u}}\right)}^{2}\right)}^{{\partial }_{k+1}}\right)\right)}^{{\Upsilon}_{{\ell}+1}};$$

$$\widehat{{\rho }_{1}}=\prod_{j=1}^{{\ell}}{\left(\prod_{i=1}^{k}{\left({\left({\eta }_{\mathrm{p}\left({a}_{{{t^{\prime}}}\left(i\right)\mathrm{z}\left(j\right)}\right)}^{l}\right)}^{2}\right)}^{{\partial }_{i}}\right)}^{{\Upsilon}_{j}};$$

$$\widehat{{\varrho }_{1}}=\prod_{j=1}^{{\ell}}{\left(\prod_{i=1}^{k}{\left(2-{\left({\eta }_{\mathrm{p}\left({a}_{{{t^{\prime}}}\left(i\right)\mathrm{z}\left(j\right)}\right)}^{l}\right)}^{2}\right)}^{{\partial }_{i}}\right)}^{{\Upsilon}_{j}};$$

$$\widehat{{\Gamma }_{1}}=\prod_{j=1}^{{\ell}}{\left(\prod_{i=1}^{k}{\left({\left({\eta }_{\mathrm{p}\left({a}_{{{t^{\prime}}}\left(i\right)\mathrm{z}\left(j\right)}\right)}^{\mathpzc{u}}\right)}^{2}\right)}^{{\partial }_{i}}\right)}^{{\Upsilon}_{j}};$$

$$\widehat{{\xi }_{1}}=\prod_{j=1}^{{\ell}}{\left(\prod_{i=1}^{k}{\left(2-{\left({\eta }_{\mathrm{p}\left({a}_{{{t^{\prime}}}\left(i\right)\mathrm{z}\left(j\right)}\right)}^{\mathpzc{u}}\right)}^{2}\right)}^{{\partial }_{i}}\right)}^{{\Upsilon}_{j}};$$

$$\widehat{{\rho }_{2}}={\left({\left({\left({\eta }_{{a}_{\left(k+1\right)\left({\ell}+1\right)}}^{l}\right)}^{2}\right)}^{{\partial }_{k+1}}\right)}^{{\Upsilon}_{{\ell}+1}};$$

$$\widehat{{\varrho }_{2}}={\left({\left(2-{\left({\eta }_{{a}_{\left(k+1\right)\left({\ell}+1\right)}}^{l}\right)}^{2}\right)}^{{\partial }_{k+1}}\right)}^{{\Upsilon}_{{\ell}+1}};$$

$$\widehat{{\Gamma }_{2}}={\left(\left({\left({\left({\eta }_{{a}_{\left(k+1\right)\left({\ell}+1\right)}}^{\mathpzc{u}}\right)}^{2}\right)}^{{\partial }_{k+1}}\right)\right)}^{{\Upsilon}_{{\ell}+1}};$$

And $$\widehat{{\xi }_{2}}={\left({\left(2-{\left({\eta }_{{a}_{\left(k+1\right)\left({\ell}+1\right)}}^{\mathpzc{u}}\right)}^{2}\right)}^{{\partial }_{k+1}}\right)}^{{\Upsilon}_{{\ell}+1}}$$. So,

$$IVPFSEOWA\left({\Delta }_{{a}_{11}},{\Delta }_{{a}_{12}},\dots \dots {\Delta }_{{a}_{\left(k+1\right)\left({\ell}+1\right)}}\right)$$

$$= \left(\left[\begin{array}{c}\frac{\sqrt{{\rho }_{1}-{\varrho }_{1}}}{\sqrt{{\rho }_{1}+{\varrho }_{1}}},\frac{\sqrt{\widehat{{\Gamma }_{1}}-\widehat{{\xi }_{1}}}}{\sqrt{\widehat{{\Gamma }_{1}}-\widehat{{\xi }_{1}}}}\end{array}\right], \left[\begin{array}{c}\frac{\sqrt{2\widehat{{\rho }_{1}}}}{\sqrt{\widehat{{\rho }_{1}}+\widehat{{\varrho }_{1}}}},\frac{\sqrt{2\widehat{{\varrho }_{1}}}}{\sqrt{\widehat{{\rho }_{1}}+\widehat{{\varrho }_{1}}}}\end{array}\right]\right)$$, and

$${\Upsilon}_{\left({\ell}+1\right)}\left({\partial }_{\left(k+1\right)}{\Delta }_{{a}_{\left(k+1\right)\left({\ell}+1\right)}}\right)$$

$$=\left(\left[\begin{array}{c}\frac{\sqrt{{\rho }_{2}-{\varrho }_{2}}}{\sqrt{{\rho }_{2}+{\varrho }_{2}}},\frac{\sqrt{\widehat{{\Gamma }_{2}}-\widehat{{\xi }_{2}}}}{\sqrt{\widehat{{\Gamma }_{2}}-\widehat{{\xi }_{2}}}}\end{array}\right], \left[\begin{array}{c}\frac{\sqrt{2\widehat{{\rho }_{2}}}}{\sqrt{\widehat{{\rho }_{2}}+\widehat{{\varrho }_{2}}}},\frac{\sqrt{2\widehat{{\varrho }_{2}}}}{\sqrt{\widehat{{\rho }_{2}}+\widehat{{\varrho }_{2}}}}\end{array}\right]\right)$$

Thus,

$$IVPFSEOWA\left({\Delta }_{{a}_{11}},{\Delta }_{{a}_{12}},\dots \dots {\Delta }_{{a}_{\left(k+1\right)\left({\ell}+1\right)}}\right){\oplus }_{\varepsilon }{\Upsilon}_{\left({\ell}+1\right)}\left({\partial }_{\left(k+1\right)}{\Delta }_{{a}_{\left(k+1\right)\left({\ell}+1\right)}}\right)$$

$$=\left(\left[\begin{array}{c}\frac{\sqrt{{\rho }_{1}-{\varrho }_{1}}}{\sqrt{{\rho }_{1}+{\varrho }_{1}}},\frac{\sqrt{\widehat{{\Gamma }_{1}}-\widehat{{\xi }_{1}}}}{\sqrt{\widehat{{\Gamma }_{1}}-\widehat{{\xi }_{1}}}}\end{array}\right], \left[\begin{array}{c}\frac{\sqrt{2\widehat{{\rho }_{1}}}}{\sqrt{\widehat{{\rho }_{1}}+\widehat{{\varrho }_{1}}}},\frac{\sqrt{2\widehat{{\varrho }_{1}}}}{\sqrt{\widehat{{\rho }_{1}}+\widehat{{\varrho }_{1}}}}\end{array}\right]\right){\oplus }_{\varepsilon }\left(\left[\begin{array}{c}\frac{\sqrt{{\rho }_{2}-{\varrho }_{2}}}{\sqrt{{\rho }_{2}+{\varrho }_{2}}},\frac{\sqrt{\widehat{{\Gamma }_{2}}-\widehat{{\xi }_{2}}}}{\sqrt{\widehat{{\Gamma }_{2}}-\widehat{{\xi }_{2}}}}\end{array}\right], \left[\begin{array}{c}\frac{\sqrt{2\widehat{{\rho }_{2}}}}{\sqrt{\widehat{{\rho }_{2}}+\widehat{{\varrho }_{2}}}},\frac{\sqrt{2\widehat{{\varrho }_{2}}}}{\sqrt{\widehat{{\rho }_{2}}+\widehat{{\varrho }_{2}}}}\end{array}\right]\right)$$

$$=\left(\begin{array}{c}\left[\begin{array}{c}\frac{\sqrt{\prod_{j=1}^{{\ell}+1}{\left(\prod_{i=1}^{k+1}{\left(1+{\left({\lambda }_{p\left({a}_{{t^{\prime}}\left(i\right)z\left(j\right)}\right)}^{l}\right)}^{2}\right)}^{{\partial }_{i}}\right)}^{{\Upsilon}_{j}}-\prod_{j=1}^{{\ell}+1}{\left(\prod_{i=1}^{k+1}{\left(1-{\left({\lambda }_{p\left({a}_{{t^{\prime}}\left(i\right)z\left(j\right)}\right)}^{l}\right)}^{2}\right)}^{{\partial }_{i}}\right)}^{{\Upsilon}_{j}}}}{\sqrt{\prod_{j=1}^{{\ell}+1}{\left(\prod_{i=1}^{k+1}{\left(1+{\left({\lambda }_{p\left({a}_{{t^{\prime}}\left(i\right)z\left(j\right)}\right)}^{l}\right)}^{2}\right)}^{{\partial }_{i}}\right)}^{{\Upsilon}_{j}}+\prod_{j=1}^{{\ell}+1}{\left(\prod_{i=1}^{k+1}{\left(1-{\left({\lambda }_{p\left({a}_{{t^{\prime}}\left(i\right)z\left(j\right)}\right)}^{l}\right)}^{2}\right)}^{{\partial }_{i}}\right)}^{{\Upsilon}_{j}}}},\\ \frac{\sqrt{\prod_{j=1}^{{\ell}+1}{\left(\prod_{i=1}^{k+1}{\left(1+{\left({\lambda }_{p\left({a}_{{t^{\prime}}\left(i\right)z\left(j\right)}\right)}^{\mathpzc{u}}\right)}^{2}\right)}^{{\partial }_{i}}\right)}^{{\Upsilon}_{j}}-\prod_{j=1}^{{\ell}+1}{\left(\prod_{i=1}^{k+1}{\left(1-{\left({\lambda }_{p\left({a}_{{t^{\prime}}\left(i\right)z\left(j\right)}\right)}^{\mathpzc{u}}\right)}^{2}\right)}^{{\partial }_{i}}\right)}^{{\Upsilon}_{j}}}}{\sqrt{\prod_{j=1}^{{\ell}+1}{\left(\prod_{i=1}^{k+1}{\left(1+{\left({\lambda }_{p\left({a}_{{t^{\prime}}\left(i\right)z\left(j\right)}\right)}^{\mathpzc{u}}\right)}^{2}\right)}^{{\partial }_{i}}\right)}^{{\Upsilon}_{j}}+\prod_{j=1}^{{\ell}+1}{\left(\prod_{i=1}^{k+1}{\left(1-{\left({\lambda }_{p\left({a}_{{t^{\prime}}\left(i\right)z\left(j\right)}\right)}^{\mathpzc{u}}\right)}^{2}\right)}^{{\partial }_{i}}\right)}^{{\Upsilon}_{j}}}}\end{array}\right],\\ \left[\begin{array}{c}\frac{\sqrt{2\prod_{j=1}^{{\ell}+1}{\left(\prod_{i=1}^{k+1}{\left({\left({\eta }_{p\left({a}_{{t^{\prime}}\left(i\right)z\left(j\right)}\right)}^{l}\right)}^{2}\right)}^{{\partial }_{i}}\right)}^{{\Upsilon}_{j}}}}{\sqrt{\prod_{j=1}^{{\ell}+1}{\left(\prod_{i=1}^{k+1}{\left(2-{\left({\eta }_{p\left({a}_{{t^{\prime}}\left(i\right)z\left(j\right)}\right)}^{l}\right)}^{2}\right)}^{{\partial }_{i}}\right)}^{{\Upsilon}_{j}}+\prod_{j=1}^{{\ell}+1}{\left(\prod_{i=1}^{k+1}{\left({\left({\eta }_{p\left({a}_{{t^{\prime}}\left(i\right)z\left(j\right)}\right)}^{l}\right)}^{2}\right)}^{{\partial }_{i}}\right)}^{{\Upsilon}_{j}}}},\\ \frac{\sqrt{2\prod_{j=1}^{{\ell}+1}{\left(\prod_{i=1}^{k+1}{\left({\left({\eta }_{p\left({a}_{{t^{\prime}}\left(i\right)z\left(j\right)}\right)}^{\mathpzc{u}}\right)}^{2}\right)}^{{\partial }_{i}}\right)}^{{\Upsilon}_{j}}}}{\sqrt{\prod_{j=1}^{{\ell}+1}{\left(\prod_{i=1}^{k+1}{\left(2-{\left({\eta }_{p\left({a}_{{t^{\prime}}\left(i\right)z\left(j\right)}\right)}^{\mathpzc{u}}\right)}^{2}\right)}^{{\partial }_{i}}\right)}^{{\Upsilon}_{j}}+\prod_{j=1}^{{\ell}+1}{\left(\prod_{i=1}^{k+1}{\left({\left({\eta }_{p\left({a}_{{t^{\prime}}\left(i\right)z\left(j\right)}\right)}^{\mathpzc{u}}\right)}^{2}\right)}^{{\partial }_{i}}\right)}^{{\Upsilon}_{j}}}}\end{array}\right]\end{array}\right)$$

For $n=k+1$ and $m=l+1$, Eq. 3 holds. So, we can say that it will be held $\forall n, m>0$.

As stated in Theorem 4.3, the consequence after using the IVPFSEOWA operators is precisely an IVPFSN. The subsequent justification can be employed to verify this contention:

$\Delta { }_{{a}_{ij}}=\left(\left[{\lambda }_{{a}_{ij}}^{l},{\lambda }_{{a}_{ij}}^{\mathpzc{u}}\right],\left[{\eta }_{{a}_{ij}}^{l},{\eta }_{{a}_{ij}}^{\mathpzc{u}}\right]\right)$ is an IVPFSV, so $0\le {\lambda }_{{a}_{ij}}^{l},{\lambda }_{{a}_{ij}}^{\mathpzc{u}},{\eta }_{{a}_{ij}}^{l},{\eta }_{{a}_{ij}}^{\mathpzc{u}}\le 1 and {\left({\lambda }_{{a}_{ij}}^{\mathpzc{u}}\right)}^{2}+{\left({\eta }_{{a}_{ij}}^{\mathpzc{u}}\right)}^{2}\le 1$.

$$0\le \frac{\sqrt{\prod_{j=1}^{m}{\left(\prod_{i=1}^{n}{\left(1+{\left({\lambda }_{p\left({a}_{{t^{\prime}}\left(i\right)z\left(j\right)}\right)}^{l}\right)}^{2}\right)}^{{\partial }_{i}}\right)}^{{\Upsilon}_{j}}-\prod_{j=1}^{m}{\left(\prod_{i=1}^{n}{\left(1-{\left({\lambda }_{p\left({a}_{{t^{\prime}}\left(i\right)z\left(j\right)}\right)}^{l}\right)}^{2}\right)}^{{\partial }_{i}}\right)}^{{\Upsilon}_{j}}}}{\sqrt{\prod_{j=1}^{m}{\left(\prod_{i=1}^{n}{\left(1+{\left({\lambda }_{p\left({a}_{{t^{\prime}}\left(i\right)z\left(j\right)}\right)}^{l}\right)}^{2}\right)}^{{\partial }_{i}}\right)}^{{\Upsilon}_{j}}+\prod_{j=1}^{m}{\left(\prod_{i=1}^{n}{\left(1-{\left({\lambda }_{p\left({a}_{{t^{\prime}}\left(i\right)z\left(j\right)}\right)}^{l}\right)}^{2}\right)}^{{\partial }_{i}}\right)}^{{\Upsilon}_{j}}}}$$

$$,\frac{\sqrt{\prod_{j=1}^{m}{\left(\prod_{i=1}^{n}{\left(1+{\left({\lambda }_{\mathrm{p}\left({a}_{{{t^{\prime}}}\left(i\right)\mathrm{z}\left(j\right)}\right)}^{\mathpzc{u}}\right)}^{2}\right)}^{{\partial }_{i}}\right)}^{{\Upsilon}_{j}}-\prod_{j=1}^{m}{\left(\prod_{i=1}^{n}{\left(1-{\left({\lambda }_{\mathrm{p}\left({a}_{{{t^{\prime}}}\left(i\right)\mathrm{z}\left(j\right)}\right)}^{\mathpzc{u}}\right)}^{2}\right)}^{{\partial }_{i}}\right)}^{{\Upsilon}_{j}}}}{\sqrt{\prod_{j=1}^{m}{\left(\prod_{i=1}^{n}{\left(1+{\left({\lambda }_{\mathrm{p}\left({a}_{{{t^{\prime}}}\left(i\right)\mathrm{z}\left(j\right)}\right)}^{\mathpzc{u}}\right)}^{2}\right)}^{{\partial }_{i}}\right)}^{{\Upsilon}_{j}}+\prod_{j=1}^{m}{\left(\prod_{i=1}^{n}{\left(1-{\left({\lambda }_{\mathrm{p}\left({a}_{{{t^{\prime}}}\left(i\right)\mathrm{z}\left(j\right)}\right)}^{\mathpzc{u}}\right)}^{2}\right)}^{{\partial }_{i}}\right)}^{{\Upsilon}_{j}}}}\le 1.$$

Since,

$$0\le {\eta }_{{a}_{ij}}^{l}\le 1, 0\le {\left({\eta }_{{a}_{ij}}^{l}\right)}^{2}\le 1, 1\le 2-{\left({\eta }_{{a}_{ij}}^{l}\right)}^{2}\le 2 ,\text{ so},$$

$$0\le {\left({\eta }_{{a}_{ij}}^{l}\right)}^{2}\le 2-{\left({\eta }_{{a}_{ij}}^{l}\right)}^{2}\le 2.\text{ So},$$

$$0\le \frac{\sqrt{2\prod_{j=1}^{m}{\left(\prod_{i=1}^{n}{\left({\left({\eta }_{p\left({a}_{{t^{\prime}}\left(i\right)z\left(j\right)}\right)}^{l}\right)}^{2}\right)}^{{\partial }_{i}}\right)}^{{\Upsilon}_{j}}}}{\sqrt{\prod_{j=1}^{m}{\left(\prod_{i=1}^{n}{\left(2-{\left({\eta }_{p\left({a}_{{t^{\prime}}\left(i\right)z\left(j\right)}\right)}^{l}\right)}^{2}\right)}^{{\partial }_{i}}\right)}^{{\Upsilon}_{j}}+\prod_{j=1}^{m}{\left(\prod_{i=1}^{n}{\left({\left({\eta }_{p\left({a}_{{t^{\prime}}\left(i\right)z\left(j\right)}\right)}^{l}\right)}^{2}\right)}^{{\partial }_{i}}\right)}^{{\Upsilon}_{j}}}}$$

$$\le \begin{array}{c}\frac{\sqrt{\prod_{j=1}^{m}{\left(\prod_{i=1}^{n}{\left(2-{\left({\eta }_{\mathrm{p}\left({a}_{{{t^{\prime}}}\left(i\right)\mathrm{z}\left(j\right)}\right)}^{l}\right)}^{2}\right)}^{{\partial }_{i}}\right)}^{{\Upsilon}_{j}}+\prod_{j=1}^{m}{\left(\prod_{i=1}^{n}{\left({\left({\eta }_{\mathrm{p}\left({a}_{{{t^{\prime}}}\left(i\right)\mathrm{z}\left(j\right)}\right)}^{l}\right)}^{2}\right)}^{{\partial }_{i}}\right)}^{{\Upsilon}_{j}}}}{\sqrt{\prod_{j=1}^{m}{\left(\prod_{i=1}^{n}{\left(2-{\left({\eta }_{\mathrm{p}\left({a}_{{{t^{\prime}}}\left(i\right)\mathrm{z}\left(j\right)}\right)}^{l}\right)}^{2}\right)}^{{\partial }_{i}}\right)}^{{\Upsilon}_{j}}+\prod_{j=1}^{m}{\left(\prod_{i=1}^{n}{\left({\left({\eta }_{\mathrm{p}\left({a}_{{{t^{\prime}}}\left(i\right)\mathrm{z}\left(j\right)}\right)}^{l}\right)}^{2}\right)}^{{\partial }_{i}}\right)}^{{\Upsilon}_{j}}}}\end{array}=1.$$

Similarly,

$$0\le \frac{\sqrt{2\prod_{j=1}^{m}{\left(\prod_{i=1}^{n}{\left({\left({\eta }_{p\left({a}_{{t^{\prime}}\left(i\right)z\left(j\right)}\right)}^{\mathpzc{u}}\right)}^{2}\right)}^{{\partial }_{i}}\right)}^{{\Upsilon}_{j}}}}{\sqrt{\prod_{j=1}^{m}{\left(\prod_{i=1}^{n}{\left(2-{\left({\eta }_{p\left({a}_{{t^{\prime}}\left(i\right)z\left(j\right)}\right)}^{\mathpzc{u}}\right)}^{2}\right)}^{{\partial }_{i}}\right)}^{{\Upsilon}_{j}}+\prod_{j=1}^{m}{\left(\prod_{i=1}^{n}{\left({\left({\eta }_{p\left({a}_{{t^{\prime}}\left(i\right)z\left(j\right)}\right)}^{\mathpzc{u}}\right)}^{2}\right)}^{{\partial }_{i}}\right)}^{{\Upsilon}_{j}}}}$$

$$\le \begin{array}{c}\frac{\sqrt{\prod_{j=1}^{m}{\left(\prod_{i=1}^{n}{\left(2-{\left({\eta }_{\mathrm{p}\left({a}_{{{t^{\prime}}}\left(i\right)\mathrm{z}\left(j\right)}\right)}^{\mathpzc{u}}\right)}^{q}\right)}^{{\partial }_{i}}\right)}^{{\Upsilon}_{j}}+\prod_{j=1}^{m}{\left(\prod_{i=1}^{n}{\left({\left({\eta }_{\mathrm{p}\left({a}_{{{t^{\prime}}}\left(i\right)\mathrm{z}\left(j\right)}\right)}^{\mathpzc{u}}\right)}^{q}\right)}^{{\partial }_{i}}\right)}^{{\Upsilon}_{j}}}}{\sqrt{\prod_{j=1}^{m}{\left(\prod_{i=1}^{n}{\left(2-{\left({\eta }_{\mathrm{p}\left({a}_{{{t^{\prime}}}\left(i\right)\mathrm{z}\left(j\right)}\right)}^{\mathpzc{u}}\right)}^{q}\right)}^{{\partial }_{i}}\right)}^{{\Upsilon}_{j}}+\prod_{j=1}^{m}{\left(\prod_{i=1}^{n}{\left({\left({\eta }_{\mathrm{p}\left({a}_{{{t^{\prime}}}\left(i\right)\mathrm{z}\left(j\right)}\right)}^{\mathpzc{u}}\right)}^{q}\right)}^{{\partial }_{i}}\right)}^{{\Upsilon}_{j}}}}\end{array}=1.$$

Since $${\left({\lambda }_{p\left({a}_{{t^{\prime}}\left(i\right)z\left(j\right)}\right)}^{\mathpzc{u}}\right)}^{2}+{\left({\eta }_{p\left({a}_{{t^{\prime}}\left(i\right)z\left(j\right)}\right)}^{\mathpzc{u}}\right)}^{2}\le 1$$,

so, $$\prod_{j=1}^{m}{\left(\prod_{i=1}^{n}{\left({\left({\eta }_{p\left({a}_{{t^{\prime}}\left(i\right)z\left(j\right)}\right)}^{\mathpzc{u}}\right)}^{2}\right)}^{{\partial }_{i}}\right)}^{{\Upsilon}_{j}}\le \prod_{j=1}^{m}{\left(\prod_{i=1}^{n}{\left(1-{\left({\lambda }_{p\left({a}_{{t^{\prime}}\left(i\right)z\left(j\right)}\right)}^{\mathpzc{u}}\right)}^{2}\right)}^{{\partial }_{i}}\right)}^{{\Upsilon}_{j}},$$

$$\prod_{j=1}^{m}{\left(\prod_{i=1}^{n}{\left(1+{\left({\lambda }_{p\left({a}_{{t^{\prime}}\left(i\right)z\left(j\right)}\right)}^{\mathpzc{u}}\right)}^{2}\right)}^{{\partial }_{i}}\right)}^{{\Upsilon}_{j}}\le \prod_{j=1}^{m}{\left(\prod_{i=1}^{n}{\left({2-\left({\eta }_{p\left({a}_{{t^{\prime}}\left(i\right)z\left(j\right)}\right)}^{\mathpzc{u}}\right)}^{2}\right)}^{{\partial }_{i}}\right)}^{{\Upsilon}_{j}}$$. Then,

$${\left(\frac{\sqrt{\prod_{j=1}^{m}{\left(\prod_{i=1}^{n}{\left(1+{\left({\lambda }_{p\left({a}_{{t^{\prime}}\left(i\right)z\left(j\right)}\right)}^{\mathpzc{u}}\right)}^{2}\right)}^{{\partial }_{i}}\right)}^{{\Upsilon}_{j}}-\prod_{j=1}^{m}{\left(\prod_{i=1}^{n}{\left(1-{\left({\lambda }_{p\left({a}_{{t^{\prime}}\left(i\right)z\left(j\right)}\right)}^{\mathpzc{u}}\right)}^{2}\right)}^{{\partial }_{i}}\right)}^{{\Upsilon}_{j}}}}{\sqrt{\prod_{j=1}^{m}{\left(\prod_{i=1}^{n}{\left(1+{\left({\lambda }_{p\left({a}_{{t^{\prime}}\left(i\right)z\left(j\right)}\right)}^{\mathpzc{u}}\right)}^{2}\right)}^{{\partial }_{i}}\right)}^{{\Upsilon}_{j}}+\prod_{j=1}^{m}{\left(\prod_{i=1}^{n}{\left(1-{\left({\lambda }_{p\left({a}_{{t^{\prime}}\left(i\right)z\left(j\right)}\right)}^{\mathpzc{u}}\right)}^{2}\right)}^{{\partial }_{i}}\right)}^{{\Upsilon}_{j}}}}\right)}^{2}$$

$$+{\left(\frac{\sqrt{2\prod_{j=1}^{m}{\left(\prod_{i=1}^{n}{\left({\left({\eta }_{p\left({a}_{{t^{\prime}}\left(i\right)z\left(j\right)}\right)}^{\mathpzc{u}}\right)}^{2}\right)}^{{\partial }_{i}}\right)}^{{\Upsilon}_{j}}}}{\sqrt{\prod_{j=1}^{m}{\left(\prod_{i=1}^{n}{\left({2-\left({\eta }_{p\left({a}_{{t^{\prime}}\left(i\right)z\left(j\right)}\right)}^{\mathpzc{u}}\right)}^{2}\right)}^{{\partial }_{i}}\right)}^{{\Upsilon}_{j}}+\prod_{j=1}^{m}{\left(\prod_{i=1}^{n}{\left({\left({\eta }_{p\left({a}_{{t^{\prime}}\left(i\right)z\left(j\right)}\right)}^{\mathpzc{u}}\right)}^{2}\right)}^{{\partial }_{i}}\right)}^{{\Upsilon}_{j}}}}\right)}^{2}.$$

$$=\frac{\prod_{j=1}^{m}{\left(\prod_{i=1}^{n}{\left(1+{\left({\lambda }_{p\left({a}_{{t^{\prime}}\left(i\right)z\left(j\right)}\right)}^{\mathpzc{u}}\right)}^{2}\right)}^{{\partial }_{i}}\right)}^{{\Upsilon}_{j}}-\prod_{j=1}^{m}{\left(\prod_{i=1}^{n}{\left(1-{\left({\lambda }_{p\left({a}_{{t^{\prime}}\left(i\right)z\left(j\right)}\right)}^{\mathpzc{u}}\right)}^{2}\right)}^{{\partial }_{i}}\right)}^{{\Upsilon}_{j}}}{\prod_{j=1}^{m}{\left(\prod_{i=1}^{n}{\left(1+{\left({\lambda }_{p\left({a}_{{t^{\prime}}\left(i\right)z\left(j\right)}\right)}^{\mathpzc{u}}\right)}^{2}\right)}^{{\partial }_{i}}\right)}^{{\Upsilon}_{j}}+\prod_{j=1}^{m}{\left(\prod_{i=1}^{n}{\left(1-{\left({\lambda }_{p\left({a}_{{t^{\prime}}\left(i\right)z\left(j\right)}\right)}^{\mathpzc{u}}\right)}^{2}\right)}^{{\partial }_{i}}\right)}^{{\Upsilon}_{j}}}$$

$$+\frac{2\prod_{j=1}^{m}{\left(\prod_{i=1}^{n}{\left({\left({\eta }_{p\left({a}_{{t^{\prime}}\left(i\right)z\left(j\right)}\right)}^{\mathpzc{u}}\right)}^{\mathpzc{u}}\right)}^{{\partial }_{i}}\right)}^{{\Upsilon}_{j}}}{\prod_{j=1}^{m}{\left(\prod_{i=1}^{n}{\left({2-\left({\eta }_{p\left({a}_{{t^{\prime}}\left(i\right)z\left(j\right)}\right)}^{\mathpzc{u}}\right)}^{2}\right)}^{{\partial }_{i}}\right)}^{{\Upsilon}_{j}}+\prod_{j=1}^{m}{\left(\prod_{i=1}^{n}{\left({\left({\eta }_{p\left({a}_{{t^{\prime}}\left(i\right)z\left(j\right)}\right)}^{\mathpzc{u}}\right)}^{2}\right)}^{{\partial }_{i}}\right)}^{{\Upsilon}_{j}}}$$

$$\le \frac{\prod_{j=1}^{m}{\left(\prod_{i=1}^{n}{\left(1+{\left({\lambda }_{p\left({a}_{{t^{\prime}}\left(i\right)z\left(j\right)}\right)}^{\mathpzc{u}}\right)}^{2}\right)}^{{\partial }_{i}}\right)}^{{\Upsilon}_{j}}-\prod_{j=1}^{m}{\left(\prod_{i=1}^{n}{\left({\left({\eta }_{p\left({a}_{{t^{\prime}}\left(i\right)z\left(j\right)}\right)}^{\mathpzc{u}}\right)}^{2}\right)}^{{\partial }_{i}}\right)}^{{\Upsilon}_{j}}}{\prod_{j=1}^{m}{\left(\prod_{i=1}^{n}{\left(1+{\left({\lambda }_{p\left({a}_{{t^{\prime}}\left(i\right)z\left(j\right)}\right)}^{\mathpzc{u}}\right)}^{2}\right)}^{{\partial }_{i}}\right)}^{{\Upsilon}_{j}}+\prod_{j=1}^{m}{\left(\prod_{i=1}^{n}{\left({\left({\eta }_{p\left({a}_{{t^{\prime}}\left(i\right)z\left(j\right)}\right)}^{\mathpzc{u}}\right)}^{2}\right)}^{{\partial }_{i}}\right)}^{{\Upsilon}_{j}}}$$

$$+\frac{2\prod_{j=1}^{m}{\left(\prod_{i=1}^{n}{\left({\left({\eta }_{\mathrm{p}\left({a}_{{{t^{\prime}}}\left(i\right)\mathrm{z}\left(j\right)}\right)}^{\mathpzc{u}}\right)}^{2}\right)}^{{\partial }_{i}}\right)}^{{\Upsilon}_{j}}}{\prod_{j=1}^{m}{\left(\prod_{i=1}^{n}{\left(1+{\left({\lambda }_{\mathrm{p}\left({a}_{{{t^{\prime}}}\left(i\right)\mathrm{z}\left(j\right)}\right)}^{\mathpzc{u}}\right)}^{2}\right)}^{{\partial }_{i}}\right)}^{{\Upsilon}_{j}}+\prod_{j=1}^{m}{\left(\prod_{i=1}^{n}{\left({\left({\eta }_{\mathrm{p}\left({a}_{{{t^{\prime}}}\left(i\right)\mathrm{z}\left(j\right)}\right)}^{\mathpzc{u}}\right)}^{2}\right)}^{{\partial }_{i}}\right)}^{{\Upsilon}_{j}}}=1.$$

The cumulative outcome of Eq. 3 is still expressed as an IVPFSV.
